# Supplementary material for: The Effects and Costs of Personalized Budgets for People with Disabilities: A Systematic Review
Source: Int J Environ Res Public Health. 2022 Dec 4;19(23):16225. doi: 10.3390/ijerph192316225 (PMC9739011; doi:10.3390/ijerph192316225)
Supplement: Supplementary file 1 [file ijerph-19-16225-s001.zip › ijerph-2031563-supplementary.pdf]

# Supplementary Materials

Table S1. Search strings with PICOS framework

| | | Definition | Keywords |
|---|---|---|---|
| **P** | Population | People with disabilities | • Intellectual disability<br>• Learning disability<br>• Developmental disability<br>• Physical disability<br>• Sensory disability<br>• Mental health<br>• Autism |
| **I** | Intervention | Personal budget | • Personal(ised) budget/funding<br>• Individual(ised) budget/funding<br>• Self-directed support<br>• Self-directed care<br>• Self-directed budget<br>• Direct payment<br>• Personalization<br>• Patient held budget<br>• Person centered budget<br>• Personal health budget<br>• Managed budget<br>• Health budget<br>• Social budget<br>• Person centered care<br>• Consumer directed care<br>• Cash for care<br>• Cash and Counseling |
| **C** | Comparison | Traditional system that would allocate the budget to the institution (service provider) | • Services as usual<br>• Care as usual<br>• Traditional services |
| **O** | Outcome | Studies that report any quantitative findings from personal budget schemes in terms of cost or health-related effects | • Cost/effectiveness<br>• Cost benefit<br>• Cost analysis<br>• Costing<br>• Value for money<br>• Service use<br>• Service cost<br>• Satisfaction with care<br>• Quality of life |

| | | | |
|---|---|---|---|
| | | | • Wellbeing<br>• Health<br>• Choice and control |
| **S** | Study Design | Studies including quantitative components | • Experimental<br>• Survey<br>• Controlled trial<br>• Questionnaire<br>• Evaluation<br>• Assessment<br>• Before-after study<br>• Pre-post study |

Table S2. Psycinfo

Accessed with Ovid through Luxembourg Library (conducted by MR)

| | | **Search terms** (advanced search) | **Number of articles retrieved 21/08/2020** | **Number of articles retrieved 15/11/2022** |
|---|---|---|---|---|
| **P** | 1 | (disabil* or mental health or autism).mp | 403,020 | 481,836 |
| **I** | 2 | (cash and care).mp | 472 | 552 |
| | 3 | (cash and counsel *).mp | 116 | 126 |
| | 4 | (personal* adj (budget or funding)).mp | 29 | 35 |
| | 5 | (individual* adj (budget or funding)).mp | 62 | 74 |
| | 6 | (self directed adj2 (support or care or budget)).mp | 142 | 155 |
| | 7 | (consumer directed adj2 (support or care or budget)).mp | 102 | 117 |
| | 8 | direct payment.mp | 34 | 37 |
| | 9 | personali?ation.mp | 2,594 | 3,787 |
| | 10 | (budget adj1 (managed or social or patient held or personal health)).mp | 10 | 12 |
| | 11 | (person adj1 (center* or centr*) adj1 (care or support or budget)).mp | 1,217 | 1,768 |
| | 12 | 2 or 3 or 4 or 5 or 6 or 7 or 8 or 9 or 10 or 11 | 4,612 | 6,478 |
| | 13 | 1 and 12 | 748 | 1,058 |
| | 14 | limit 13 to yr="1985-2020" ("2020-2022") | 743 | 313 |

*indicates multiple suffixes

Table S3. Medline

Accessed with Ovid through Luxembourg Library (conducted by MR)

| | | **Search terms** (advanced search) | **Number of articles retrieved** | **Number of articles retrieved** |
|---|---|---|---|---|

| | | | 12/08/2020 | 15/11/2022 |
|---|---|---|---|---|
| **P** | 1 | (disabil* or mental health or autism).mp | 488,746 | 596,346 |
| **I** | 2 | (cash and care).mp | 1,439 | 1,652 |
| | 3 | (cash and counsel*).mp | 123 | 144 |
| | 4 | (personal* adj (budget or funding)).mp | 36 | 45 |
| | 5 | (individual* adj (budget or funding)).mp | 62 | 76 |
| | 6 | (self directed adj2 (support or care or budget)).mp | 120 | 145 |
| | 7 | (consumer directed adj2 (support or care or budget)).mp | 165 | 185 |
| | 8 | direct payment.mp | 93 | 101 |
| | 9 | personali?ation.mp | 2,462 | 3,957 |
| | 10 | (budget adj1 (managed or social or patient held or personal health)).mp | 29 | 37 |
| | 11 | (person adj1 (center* or centr*) adj1 (care or support or budget)).mp | 2,192 | 3,509 |
| | 12 | 2 or 3 or 4 or 5 or 6 or 7 or 8 or 9 or 10 or 11 | 6,571 | 9,671 |
| | 13 | 1 and 12 | 644 | 991 |
| | 14 | limit 13 to yr="1985-2020" ("2020-2022") | 639 | 404 |

*indicates multiple suffixes

Table S4. CINAHL

Accessed with EBSCO through Luxembourg Library (conducted by MR)

| | | **Search terms** (advanced search) | **Number of articles retrieved 21/08/2020** | **Number of articles retrieved 15/11/2022** |
|---|---|---|---|---|
| **P** | S1 | TI (disabil* OR "mental health" OR autism) OR AB(disabil* OR "mental health" OR autism) | 228,853 | 279,663 |
| **I** | S2 | TI ( "cash and care" ) OR AB ( "cash and care" ) | 44 | 50 |
| | S3 | TI ( "cash and counsel*" ) OR AB ( "cash and counsel*" ) | 48 | 51 |
| | S4 | TI ( personal W0 (budget OR funding) ) OR AB ( personal W0 (budget OR funding) ) | 113 | 160 |
| | S5 | TI ( individual W0 (budget OR funding) ) OR AB ( individual W0 (budget OR funding) ) | 82 | 109 |
| | S6 | TI ( "self directed" W2 (support OR care OR budget) ) OR AB ( "self directed" W2 (support OR care OR budget) ) | 169 | 182 |
| | S7 | TI ( "consumer directed" W2 (support OR care OR budget) ) OR AB ( "consumer directed" W2 (support OR care OR budget) ) | 153 | 177 |
| | S8 | TI "direct payment" OR AB "direct payment" | 64 | 67 |

| | | | | |
|---|---|---|---|---|
| | S9 | TI personali?ation OR AB personali?ation | 1,298 | 1,751 |
| | S10 | TI ( budget N1 (managed OR social OR patient held OR personal health) ) OR AB ( budget N1 (managed OR social OR patient held OR personal health) ) | 145 | 221 |
| | S11 | TI ( person W0 (center* or centr*) W1 (care OR support OR budget) ) OR AB ( person W0 (center* or centr*) W1 (care OR support OR budget) ) | 2,443 | 3,436 |
| | S12 | S2 OR S3 OR S4 OR S5 OR S6 OR S7 OR S8 OR S9 OR S10 OR S11 | 4,385 | 5,992 |
| | S13 | S1 AND S12 | 596 | 770 |
| | S14 | S1 AND S12 *Limiters - Published Date: 19850101-20201231 (20200901-20221115)* | 596 | 169 |

*indicates multiple suffixes

Table S5. Social care online

Accessed online through https://www.scie-socialcareonline.org.uk/, (conducted by MR)

*Note: Due to the limitation of exporting only 500 results the query had to be broken up to ensure the results were always below 500. Four separate search queries were conducted, detailed below, which lead to a greatly inflated number of duplicates.*

For the update search, year limiters are "2020-2022".

| | **Search terms** (advanced search) | **Number of articles retrieved 26/08/2020** | **Number of articles retrieved 15/11/2022** |
|---|---|---|---|
| Search 1 | S1 [ <br>- PublicationTitle:'disability' <br>- OR AbstractOmitNorms:'disability' <br>- OR PublicationTitle:'"mental health"' <br>- OR AbstractOmitNorms:'"mental health"' <br>- OR PublicationTitle:'autism' <br>- OR AbstractOmitNorms:'autism' <br>- AND PublicationYear:'1985 2020' <br>] <br><br>**AND** <br><br>(S2: <br><br>o S2a [ <br>- PublicationTitle:'"cash and care"' | 417 | 48 |

| | | | |
|---|---|---|---|
| | - OR AbstractOmitNorms:'"cash and care"'<br>- OR PublicationTitle:'"cash and counsel*"'<br>- OR AbstractOmitNorms:'"cash and counsel*"'<br>- AND PublicationYear:'1985 2020'<br>]<br>**OR**<br>o S2b [<br>- PublicationTitle:'"self directed support"'<br>- OR AbstractOmitNorms:'"self directed support"'<br>- OR PublicationTitle:'"self directed care"'<br>- OR AbstractOmitNorms:'"self directed care"'<br>- OR PublicationTitle:'"self directed budget"'<br>- OR AbstractOmitNorms:'"self directed budget"'<br>- AND PublicationYear:'1985 2020'<br>]<br>**OR**<br>o S2c [<br>- PublicationTitle:'"consumer directed support"'<br>- OR AbstractOmitNorms:'"consumer directed support"'<br>- OR PublicationTitle:'"consumer directed care"'<br>- OR AbstractOmitNorms:'"consumer directed care"'<br>- OR PublicationTitle:'"consumer directed budget"'<br>- OR AbstractOmitNorms:'"consumer directed budget"'<br>- AND PublicationYear:'1985 2020'<br>]<br>**OR**<br>o S2d [<br>- TitleExact:'managed budget'<br>- OR AbstractOmitNormsExact:'managed budget'<br>- OR TitleExact:'social budget'<br>- OR AbstractOmitNormsExact:'social budget'<br>- OR PublicationTitle:'"patient held budget"'<br>- OR AbstractOmitNorms:'"patient held budget"'<br>- OR TitleExact:'personal health budget'<br>- OR AbstractOmitNormsExact:'personal health budget'<br>- AND PublicationYear:'1985 2020' | | |

| | | | |
|---|---|---|---|
| | ]<br>**OR**<br>o S2e [<br>- PublicationTitle:'"person centred care"'<br>- OR AbstractOmitNorms:'"person centred care"'<br>- OR PublicationTitle:'"person centered care"'<br>- OR AbstractOmitNorms:'"person centered care"'<br>- OR PublicationTitle:'"person centred support"'<br>- OR AbstractOmitNorms:'"person centred support"'<br>- OR PublicationTitle:'"person centered support"'<br>- OR AbstractOmitNorms:'"person centered support"'<br>- OR PublicationTitle:'"person centred budget"'<br>- OR AbstractOmitNorms:'"person centred budget"'<br>- OR PublicationTitle:'"person centered budget"'<br>- OR AbstractOmitNorms:'"person centered budget"'<br>- AND PublicationYear:'1985 2020'<br>]<br><br>) | | |
| Search 2 | S1 [<br>- PublicationTitle:'disability'<br>- OR AbstractOmitNorms:'disability'<br>- OR PublicationTitle:'"mental health"'<br>- OR AbstractOmitNorms:'"mental health"'<br>- OR PublicationTitle:'autism'<br>- OR AbstractOmitNorms:'autism'<br>- AND PublicationYear:'1985 2020'<br>]<br><br>**AND**<br><br>S2 [<br>- PublicationTitle:'"personal* budget"'<br>- OR AbstractOmitNorms:'"personal* budget"'<br>- OR TitleExact:'"personal* funding"'<br>- OR AbstractOmitNormsExact:'"personal* funding"'<br>- OR PublicationTitle:'"individual* budget"' | 285 | 11 |

| | | | |
|---|---|---|---|
| | - OR AbstractOmitNorms:'"individual* budget"'<br>- OR PublicationTitle:'"individual* funding"'<br>- OR AbstractOmitNorms:'"individual* funding"'<br>- AND PublicationYear:'1985 2020'<br>] | | |
| Search 3 | S1 [<br>- PublicationTitle:'disability'<br>- OR AbstractOmitNorms:'disability'<br>- OR PublicationTitle:'"mental health"'<br>- OR AbstractOmitNorms:'"mental health"'<br>- OR PublicationTitle:'autism'<br>- OR AbstractOmitNorms:'autism'<br>- AND PublicationYear:'1985 2020'<br>]<br><br>**AND**<br><br>S2 [<br>- PublicationTitle:'"direct payment"'<br>- OR AbstractOmitNorms:'"direct payment"'<br>- AND PublicationYear:'1985 2020'<br>] | 280 | 9 |
| Search 4 | S1 [<br>- PublicationTitle:'disability'<br>- OR AbstractOmitNorms:'disability'<br>- OR PublicationTitle:'"mental health"'<br>- OR AbstractOmitNorms:'"mental health"'<br>- OR PublicationTitle:'autism'<br>- OR AbstractOmitNorms:'autism'<br>- AND PublicationYear:'1985 2020'<br>]<br><br>**AND**<br><br>(S2:<br>o S2a [<br>- TitleExact:'personalisation'<br>- OR AbstractOmitNormsExact:'personalisation' | 267 | 18 |

| | | | |
|---|---|---|---|
| | - AND PublicationYear:'1985 2020'<br>]<br>**OR**<br>o S2b [<br>- TitleExact:'personalization'<br>- OR AbstractOmitNormsExact:'personalization'<br>- AND PublicationYear:'1985 2020'<br>]<br><br>) | | |

*indicates multiple suffixes

Table S6. ASSIA

Accessed via Proquest through the University of York (conducted by MS)

| **Search terms** (advanced search) | **Number of articles retrieved 29/09/2020** | **Number of articles retrieved 15/11/2022 (yr limited = from Sep. 2020)** |
|---|---|---|
| (ab(Disabil*) OR ti(disabil*) OR ab(mental health) OR ti(mental health) OR ab(autism) OR ti(autism)) AND (ab(cash AND care) OR ab(cash AND counsel*) OR ab(personal budget) OR ab(personal funding) OR ab(individual budget) OR ab(individual funding) OR ab(self directed near/2 support) OR ab(self directed near/2 care) OR ab(self directed near/2 budget)) AND (ab(consumer directed near/2 (support OR care OR budget)) OR ab(direct payment) OR ab(personali?ation) OR ab((managed OR social OR patient held OR personal health) AND (budget)) OR ab((person cent*) AND (care OR support OR budget))) | 124 | 8 |

*indicates multiple suffixes

Table S7: Excluded articles

| Author (year) | Title | Publication title ( or publisher) | Status | Reason |
|---|---|---|---|---|
| Glendinning (2008) | The national evaluation of the individual budgets pilot programme | | Duplicate | |
| Cook (2008) | Economic grand rounds: a self-directed care model for mental health recovery. | Psychiatric Services, 59, no. 6 (2008): 600-602. | Duplicate | |
| San Antonio (2009) | Lessons from the Arkansas cash and counseling program: how the experiences of diverse older consumers and their caregivers address family policy concerns | Journal of Aging & Social Policy 22, no. 1 (2009): 1-17. | Duplicate | |
| Adelman (2012) | Change and inertia in the New York State Medicaid Personal Care Services Program: an institutional case study. | Journal of Aging & Social Policy, 24, no. 3 (2012): 309-327. | Exclude | This is a study providing a descriptive analysis of the New York Personal Care Services. There was no evaluation of service user's outcomes or perspectives, qualitative or quantitative. Furthermore, this article is not specifically related to personal budgets. |
| Agosta (2010) | Using Individual Budget Allocations to support people with intellectual and developmental disabilities | In book: Using Individual Budget Allocations to Support People with Intellectual and Developmental Disabilities (pp.1-13). Edition: 1st | Exclude | This is an article providing information on the implementation of personal budget systems and the main considerations that should be taken into account. |
| Alakeson (2016) | Self-Direction in Long-Term Services and Supports: International Differences and Current Challenges | Public Policy & Aging Report, Volume 26, Issue 4, 2016, Pages 143–148 | Exclude | Description of the self-direction systems in Australia, England, Germany, Netherlands and US. No data or evaluation results provided. |

| | | | | |
|---|---|---|---|---|
| Allen (2008) | Cash control. | Learning Disability Practice. Vol. 11 no 10 December 2008 | Exclude | News article about the individual budget pilot programme in the UK. |
| Anand (2012) | The Transition to Personal Budgets for People with Disabilities : A Review of Practice in Specified Jurisdictions | National Disability Authority, Ireland | Exclude | Systematic review of previous studies |
| Andermatt (2012) | Verfahren zur individuellen bedarfsabklärung und leistungsbemessung: Schlussbericht zum Auftrag „Instrumente und Verfahren für eine individuelle Bemessung der Leistungen der Behindertenhilfe" vom 26.3.2010 | | Exclude | This discusses the process and instruments for assessing the needs with a view to personal budgeting, in the Swiss context, but it is not an evaluation. |
| Arksey (2009) | Individual budgets: impacts and outcomes for carers | IBSEN, Individual Budgets Evaluation Network | Exclude | This is just a summary of a bigger study (Glendinning 2009). |
| Arnold (2015) | Does a Measure of Support Needs Predict Funding Need Better Than a Measure of Adaptive and Maladaptive Behavior?. | American journal on intellectual and developmental disabilities, 2015 Sep;120(5):375-94 | Exclude | Focus of the article is on predicting support needs and levels of individual funding. |
| Arntz (2011) | Crowding Out Informal Care? Evidence from a Field Experiment in Germany | Oxford Bulletin of Economics and Statistics, 73, no. 3 (2011): 398-427. | Exclude | This study only considered elderly people. |
| Ash (2017) | Social Determinants of Health in Managed Care Payment Formulas. | JAMA Internal Medicine, 2017 Oct 1;177(10):1424-1430 | Exclude | This is a modelling study using social determinants of health to predict how medical benefits can be better estimated. |

| | | | | |
|---|---|---|---|---|
| Barczyk (2010) | Cash and counseling: a model for self-directed care programs to empower individuals with serious mental illnesses. | Social Work in Mental Health, 8:3, 209-224 | Exclude | Focus groups and surveys with individuals with disabilities to examine their preferences for the Cash and Counseling programme.<br>Also summaries the outcomes of the programme evaluations presented in the final report. |
| Bennett (2009) | Investigation of Individualised Funding and Local Area Coordination- Type processes: A Literature Review . | Disability Policy Disability Support Services Group Ministry of Health | Exclude | Review of individual funding and local area coordination for the New Zealand government. No evaluation. |
| Bern Kanton (2020) | Autodétermination accrue pour les personnes en situation de handicap | Communiqué de presse; Direction de la santé, des affaires sociales et de l'intégration | Exclude | This outlines future plans for disabled people in Bern |
| Bogenschutz (2010) | Evaluation of consumer directed community supports for people with intellectual and developmental disabilities in Minnesota. | Dissertation Abstracts International Section A: Humanities and Social Sciences, Faculty of the Graduate School of the University of Minnesota | Exclude | Thesis looking at why users of consumer directed community supports in the US choose to stay or remain with the programme. The data comes from a mail-in survey of service users. |
| Bowers (2017) | Care coordination experiences of people with disabilities enrolled in Medicaid managed care. | Disability & Rehabilitation, 2017 Oct;39(21):2207-2214 | Exclude | This is a study on care coordination for disabled people. There is no form of personal budget paid. |
| Brooks (2016) | Personalisation, personal budgets and family carers. Whose assessment? Whose budget? | Journal of Social Work, 17, no. 2 (2017): 147-166. | Exclude | A qualitative survey to describe the role of carers in personalisation in the UK. |
| Brown (2018) | "It's Like Two Roles We're Playing": Parent Perspectives on Navigating Self-Directed Service Programs with Adult Children with | Journal of Policy & Practice in Intellectual Disabilities, 15(4), pp.350-358. | Exclude | Presents results of a qualitative study of parents opinions and experiences |

| | | | | |
|---|---|---|---|---|
| | Intellectual and/or Developmental Disabilities. | | | |
| Brown (2007) | The research design and methodological issues for the Cash and Counseling Evaluation. | Health services research, 42, no. 1p2 (2007): 414-445. | Exclude | This paper provides details of the methodology behind the evaluation of the cash and counseling program, it does not provide outcome results. |
| Büscher (2010) | Häusliche Pflegeberatung für Geldleistungsbezieher in der Pflegeversicherung. | Zeitschrift für Gerontologie und Geriatrie. 2010 Apr 1;43(2):103-10. | Exclude | This is not on people with disabilities specifically. It focuses on long-term care insurance. |
| Caldwell (2003) | Management of respite and personal assistance services in a consumer-directed family support programme. | Journal of Intellectual Disability Research, May-Jun 2003;47(Pt 4-5):352-66 | Exclude | A study on the management of personal assistance services, and the outcomes of having control. Looks at the statistics of hiring other relatives to provide care and the associated outcome variables. No before-after or control comparison. |
| Caldwell (2007) | Experiences of families with relatives with intellectual and developmental disabilities in a consumer-directed support program | Disability & Society, 22, no. 6 (2007): 549-562. | Exclude | Qualitative survey study on family experiences of a consumer directed program in the US. No quantitative data given |
| Caldwell (2006) | Consumer-directed supports: economic, health, and social outcomes for families. | Mental retardation,44, no. 6 (2006): 405-417. | Exclude | Controlled cross-sectional survey, superseded by the more comprehensive article by Caldwell (2007). |

| | | | | |
|---|---|---|---|---|
| Campbell (2011) | Independent living strategy: support planning and brokerage: final report from the support planning and brokerage demonstration project | London, UK: Office for Disability Issues and Norah Fry Research Centre | Exclude | The quantitative data presented is not relevant. It is limited to the following: characteristics of service users, service received, time to deliver support plan and cost of delivering the plan (e.g. staff costs and overheads). No quantitative health outcomes presented. |
| Carlson (2007) | Effects of Cash and Counseling on Personal Care and Well-Being | Health services research, 42, no. 1p2 (2007): 467-487. | Exclude | All of these results are presented for adults in Carlson (2005) and for children in the Foster (2004). |
| Carr (2009) | The implementation of individual budget schemes in adult social care | (2009).London: Social Care Institute for Excellence. | Exclude | A review of individual budget schemes in the UK. No data or analysis presented. |
| Carr (2012) | Personalisation : a rough guide | Social Care Institute for Excellence, (2010). London: Social Care Institute for Excellence. | Exclude | Overview of the personalisation agenda in the UK |
| Chesire Council (2010) | Findings from the Personal Budgets Survey | Chesire Weat & Chesire Council. Research, Intelligence and Consultation Team. | Exclude | Most data is irrelevant for this review. Data on user outcomes presented on bar charts with no way to distinguish elderly from others. |
| Cook (2010) | Participatory Action Research to Establish Self-Directed Care for Mental Health Recovery in Texas. | Psychiatric rehabilitation journal, 34, no. 2 (2010): 137. | Exclude | Only very preliminary data presented for 20 participants on what they purchased with their budget. |
| Coyle (2011) | Impact of person-centred thinking and personal budgets in mental health services: reporting a UK pilot. | Journal of Psychiatric and Mental Health Nursing 18, no. 9 (2011): 796-803. | Exclude | Presents a narrative analysis of individual recovery budgets for people with serious mental health problems. No quantitative data presented. |

| | | | | |
|---|---|---|---|---|
| Craston (2014) | Evaluation of the Special Educational Needs and Disability Pathfinder Programme: thematic report: collaborative working with health: research report | Understanding the comparative costs of delivering the EHC planning and SEN Statementing processes for newcomers to the SEN system (2014). | Exclude | Evaluation of the Special Educational Needs (SEN) and Disability Pathfinder Programme, very briefly touches on the topic of personal budgets and provides no evidence, qualitative or quantitative, on their outcomes or impacts. |
| Crisp (2009) | Developing and Implementing Programs and Policies: A Handbook | | Exclude | This is a guidance document to offer information to policymakers in the US who are responsible for implementing self-direction programmes. |
| Crosby (2011) | Personalisation: children, young people and families: briefing 3: evaluation and outcomes | In Control | Exclude | Data presented in bar charts only for 47 families, taking a retrospective approach. Some families received a one-off payment and others receive a monthly payment, it is not possible to distinguish these two groups from the charts. |
| Dale (2004) | Does Arkansas' Cash and Counseling Afect Service User and Public Costs? | Mathematica Policy Research Inc. | Exclude | More detailed costs data from the Arkansas Cash and Counseling demonstration site. All relevant data for this review is contained in the final report from Brown (2007). |
| Dale (2006) | Reducing nursing home use through consumer-directed personal care services | Medical Care, (2006): 760-767. | Exclude | This contains more details on nursing care facility use and expenditures for the Arkansas Cash and Counseling demonstration site. Data is not split by elderly-nonelderly, and the focus is on new-old PCS users. Costs and service use are presented in the report by Dale (2005) |

| | | | | |
|---|---|---|---|---|
| Dale (2007) | How does cash and counseling affect costs? | Health Services Research, 42, no. 1p2 (2007): 488-509. | Exclude | Journal article summarising the results in the Dale (2005) report for adults and the Dale (2004) report for children. The only data not already presented is the ratio of actual to expected costs, which is not relevant for our review. |
| Dale (2003) | The Effects of Cash and Counseling on Personal Care Services and Medicaid Costs in Arkansas | *Health Affairs* 22, no. Suppl1 (2003): W3-566. | Exclude | Contains data for the Arkansas Cash and Counseling demonstration. Data relevant for this review is already contained in the Dale (2005) costs report. |
| Dale (2003) | The effect of consumer direction on personal assistance received in Arkansas | Mathematica Policy Research Inc. | Exclude | All relevant data presented in previous reports |
| Dale (2003) | The experiences of workers hired under consumer direction in Arkansas | Mathematica Policy Research Inc. | Exclude | Outcomes for paid workers do not fall under the scope of this review |
| Dean (2016) | Learning from mental health PHBs in Stockport | All Together Positive | Exclude | A small-scale study providing limited data on the number of visits to the emergency room for 10 people recruited through emergency departments or a local charity. |
| Dew (2013) | Carer and service providers' experiences of individual funding models for children with a disability in rural and remote areas. | Health & Social Care in the Community, 21, no. 4 (2013): 432-441. | Exclude | Qualitative study on service users experiences in Australia, based on interviews with carers and service providers. No quantitative data provided. |
| Dickey (1997) | The cost and outcomes of community-based care for the seriously mentally ill | Health Services Research, 32, no. 5 (1997): 599. | Exclude | Comparison of mental health care systems in 3 regions of Massachusetts. No personal budget payments involved in community care option. |

| | | | | |
|---|---|---|---|---|
| Dickinson (2017) | Managing care integration during the implementation of large-scale reforms: Managing Community Care | Journal of Integrated Care | Exclude | The article is the result of a survey (semi-structured interviews) of policy makers in Australia about the boundaries between the NDIS and other services. |
| Dickinson (2008) | Briefing Paper 30 The personalisation agenda : implications for the third sector | OTS Barrow Cadbury Third Sector Research Centre | Exclude | The article is an opinion piece on the larger personalisation agenda |
| Dickinson (2017) | Individual funding systems: What works? | Evidence Base 2017, no. 3 (2017): 1-18. | Exclude | Review of existing personal budget systems. |
| Dickson (2013) | The Development of a Tailored Support Needs Assessment Tool for UnitingCare Disability ' s Flexible Options Program : Life Visioning and Support Needs Assessment Tool | Prepared for Uniting Care Disability by Pathways to Leadership and Rachel Dickson Consulting | Exclude | Report on the development of a needs assessment tool. |
| Dotty (1999) | A Comparison of Client-Directed and Professional Management Models of Service Delivery | | Exclude | Non-technical companion to Benjamin (2000). |
| Doty (2007) | Designing the Cash and Counseling Demonstration and Evaluation. | Health services research, 2007 Feb; 42(1 Pt 2): 378–396. | Exclude | Provides the background information on how the Cash and Counselling program was designed, including the establishment of the demonstration sites. |
| Doyle (1995) | Disability: use of an independent living fund in south east London and users' views about the system of cash versus care provision. | Journal of Epidemiology & Community Health, 49, no. 1 (1995): 43-47. | Exclude | Interviews with 95 fund recipients, details on their opinions, purchases, and perceived advantages and disadvantages. Includes people over the age of 65, with age-related conditions such as Alzheimer's disease. No comparison with traditional care. |
| ECDP (2011) | Briefing paper 2: understanding demand: findings from the second round of a three-year longitudinal study in Essex | Office for Public Management , London. | Exclude | Linked to 4 other excluded reports |

| | | | | |
|---|---|---|---|---|
| ECDP (2011) | Briefing paper 4: service users' attitudes to risk in using personal budgets: findings from the second round of a three-year longitudinal study in Essex | Office for Public Management , London. | Exclude | Linked to 4 other excluded reports |
| ECDP (2011) | Briefing paper 1: positive impacts of cash payments for service users and their families: findings from the second round of a three-year longitudinal study in Essex | Office for Public Management , London. | Exclude | Output from a qualitative study. No details given on methodology |
| ECDP (2011) | Briefing paper 5: 'in our own words': the impact of cash payments on service users and their families: findings from the second round of a three-year longitudinal study in Essex | Office for Public Management , London. | Exclude | Linked to 4 other excluded reports |
| ECDP (2011) | Briefing paper 3: developing the service provider market to best meet the needs of holders of personal budgets: findings from the second round of a three-year longitudinal study in Essex | Office for Public Management , London. | Exclude | Linked to 4 other excluded reports |
| Edwards (2009) | It's your life: take control: the implementation of self-directed support in Hertfordshire | In Control | Exclude | Limited data presented only in bar charts (no figures) where users rated their outcomes on a 3-point scale of better/worse/same. |
| Ellis (2014) | Better lives: an evaluation of personalisation in Southwark | The Centre for Welfare Reform | Exclude | Data provided for 70 people, age up to 80. No way to remove older people. Data only presented in bar chart form (no actual figures) where carers rated outcomes on a 5-point scale on behalf of the budget recipient. |

| | | | | |
|---|---|---|---|---|
| Eost-Telling (2010) | Stockport Self Directed Support Pilot in Mental Health Final Report of the Evaluation of the Self Directed Support Pilot | University of Chester | Exclude | Qualitative data from interaction with users, carers and brokers. No relevant quantitative data. |
| Eriksson (2014) | The need for self-determination and imagination: personal budgeting and the management of disability services in Finland | Journal of Policy and Practice in Intellectual Disabilities, 11, no. 2 (2014): 137-148. | Exclude | A discussion article about the pilot project in Finland. No data or outcomes provided. |
| Eriksson (2014) | Personal Budgeting in Municipal Disability Services: The First Experiment in Finland | The Finnish Association on Intellectual and Developm ental Disabilities Center of research and Development | Exclude | Report on a trial personal budget system in Finland. Only contains qualitative results, quotes from service users, etc. |
| Fernandez (2007) | Direct payments in England: factors linked to variations in local provision | Journal of Social Policy, 36, no. 1 (2007): 97-121. | Exclude | Presents an analysis of national UK data on personal budgets, focusing on local variations. Not on user experiences or other outcomes. |
| Field (2015) | MIC Individualised Funding Analysis Report for Manawanui InCharge | Manawanui In Charge, Auckland | Exclude | Total costs to the country presented, and average per user derived. The study doesn't look at a fixed sample of individuals, for example costs could change because a person's situation changes, costs of services go up/down, etc. It is stated as a limitation that changes in costs cannot be ascribed specifically to individual funding. |
| Fisher (2010) | Effectiveness of individual funding for disability support | Social Policy Research Centre Newsletter, 105 (2010): 4-7. | Exclude | Newsletter related to the Fischer report from Australia. |
| Fisher (2010) | Effectiveness of Individual Funding Approaches for Disability Support | Australian Government | Exclude | The only comparison provided is on one outcome of "satisfaction compared to previous support": previous support is variable amongst the people included and the comparison is with the Australian population as a whole, or a small subset of people with intellectual disabilities. Plus |

| | | | | |
|---|---|---|---|---|
| | | | | this is a retrospective comparison with no baseline measurements performed. |
| Fleming (2019) | Individualised funding interventions to improve health and social care outcomes for people with a disability: a mixed-methods systematic review | Campbell Systematic Reviews, 3, 2019 | Exclude | Systematic Review |
| Fleming (2016) | The successes and challenges of implementing individualised funding and supports for disabled people: An Irish perspective. | Disability & Society, 31, no. 10 (2016): 1369-1384. | Exclude | Presents results of a study of 4 individualised funding pilot programmes funded by an NGO in Ireland. Results of qualitative interviews with service users are presented, no quantitative data. |
| Fleming (2016) | How personal budgets are working in Ireland | Genio | Exclude | Presents evidence on the practicality of introducing a personal budget system. It provides a narrative description of successes and challenges for 4 small-scale pilot programs in Ireland funded by an NGO. No outcome data provided. |
| Foster (2003) | Does Consumer Direction Affect the Quality of Medicaid Personal Assistance in Arkansas? | Mathematica Policy Research Inc. | Exclude | Present detailed information for the Arkansas Cash and Counseling demonstration site. The data relevant for this review are already contained in Carlson (2005) |
| Foster (2005) | Easing the burden of caregiving: the impact of consumer direction on primary informal caregivers in Arkansas. | The Gerontologist, 45, no. 4 (2005): 474-485. | Exclude | The data relevant for this review are already contained in the Cash and Counseling demonstration report by Foster (2003). This is a journal paper summarising those results. |
| Foster (2003) | Improving the quality of Medicaid personal assistance through consumer direction. | Health affairs, 22, no. Suppl1 (2003): W3-162. | Exclude | Journal article contains results already reported in the Cash and Counseling demonstration report by Carlson (2005) |
| Foster (2007) | How caregivers and workers fared in Cash and Counseling. | Health services research, 42, no. 1p2 (2007): 510-532. | Exclude | More data on the Cash and Counseling demonstration. Data relevant for this |

| | | | | |
|---|---|---|---|---|
| | | | | review are already contained in the final report by Brown (2007) |
| Foster (2005) | Consumer and consultant experiences in the New Jersey personal preference program | Mathematica Policy Research Inc. | Exclude | This report only considered the experiences of the treatment group in the New Jersey Cash and Counseling demonstration site, not the control group. |
| Foster (2004) | Consumer and consultant experiences in the Florida consumer directed care program | Mathematica Policy Research Inc. | Exclude | Presents data for Florida on the processes and logistics of the pilot, only for the treatment group |
| Foster (2005) | The effects of cash and counseling on the primary informal caregivers of children with developmental disabilities | Mathematica Policy Research Inc. | Exclude | Relevant data in the final report by Brown (2007) |
| Foster (2005) | Assessing the appeal of the cash and conseling demonstration in Arkansas, Florida and New Jersey. | Mathematica Policy Research Inc. | Exclude | Presents data on why people chose to participate in the Cash and Counseling demonstration. Not relevant for this review |
| Fox (2004) | Evaluating a Medicaid home and community-based physical disability waiver. | Family & Community Health, 27, no. 1 (2004): 37-51. | Exclude | This studies the same disability waiver program as the Kim (2006) paper, but does not consider consumer-directed care. |
| Gadsby (2013) | Personal Budgets and Health : a review of the evidence | Centre for Health Services Studies, University of Kent | Exclude | Literature review of existing personal budget systems |
| Gadsby (2013) | Personal Budgets, Choice and Health: A review of international evidence from 11 OECD countries | International Journal of Public and Private Healthcare Management and Economics, (IJPPHME) 3, no. 3 (2013): 15-28. | Exclude | Provides a description of personal budget systems in 11 countries, including a review on outcomes |
| Glasby (2009) | A healthy choice? Direct payments and healthcare in the English NHS | Policy and Politics, 37, no. 4 (2009): 481-497. | Exclude | This article presents a review of the status of personal budgets in the UK, It presents no analysis or evaluation data. |

| | | | | |
|---|---|---|---|---|
| Glendinning (2008) | The national evaluation of the individual budgets pilot programme: experiences and implications for care coordinators and managers | The Individual Budgets Evaluation Network (IBSEN) | Exclude | 4 page summary of outcomes from the IBSEN evaluation, Glendinning (2008) |
| Grey (2019) | Darlington Change project: devolved budgets. Interim report | | Exclude | This is a pilot study aimed at children, not necessarily with disabilities. The purpose of these budgets is to try to avoid children entering care. |
| Hamilton (2016) | Power, Choice and Control: How Do Personal Budgets Affect the Experiences of People with Mental Health Problems and Their Relationships with Social Workers and Other Practitioners? | British Journal of Social Work, 46, no. 3 (2016): 719-736. | Exclude | Qualitative results from interviews with personal budget users and service provides to examine the relationship between them. |
| Harkes (2014) | Self Directed Support and people with learning disabilities: a review of the published research evidence. | British Journal of Learning Disabilities, 42, no. 2 (2014): 87-101. | Exclude | Literature review of self-directed support in the UK. |
| Harry (2017) | Long-Term Experiences in Cash and Counseling for Young Adults with Intellectual Disabilities: Familial Programme Representative Descriptions. | Journal of Applied Research in Intellectual Disabilities, 30, no. 4 (2017): 573-583. | Exclude | Only provides a qualitative analysis of young adult outcomes from the Cash and Counseling Demonstration. |
| Harry (2017) | The Cash and Counseling model of self-directed long-term care: Effectiveness with young adults with disabilities. | Disability and health journal, 10, no. 4 (2017): 492-501. | Exclude | This is a secondary study using data from the Cash and Counseling demonstration, considering a subset of the larger study. It specifically focuses on young adults aged 18-35. These results are implicitly included in the larger study. |
| Harry (2017) | The effectiveness of participant-directed home and community-based services for young adults | Dissertation Abstracts International Section A: Humanities and Social Sciences | Exclude | Dissertation linked to the 2017 journal article by Harry. |

| | | | | |
|---|---|---|---|---|
| | with long-term care disabilities: Analysis of a randomized control trial. | | | |
| Hatton (2011) | The national personal budget survey | London: Think Local Act Personal | Exclude | Data on outcomes such as control, wellbeing and health given in the form of bar charts (no figures) with ratings on a 5-point scale. More than 43% of people are >65 with no way to remove them. No control group. |
| Hatton (2013) | The Second Poet Survey of Personal Health Budget Holders and Carers 2013 | In Control | Exclude | Uncontrolled cross-sectional survey. Users rate outcomes based on how they feel their life has improved, with no baseline measurement. |
| Hatton (2008) | A report on in Control ' s Second Phase A report on Second Phase | In Control | Exclude | No control group and no baseline comparison. |
| Health Foundation (2010) | Personal health budgets: | Health Foundation | Exclude | Review of existing evidence on personal budgets |
| Heller (2005) | Brief Research Report Impact of a Consumer-Directed Family Support Program on Reduced Out-of-Home Institutional Placement. | Journal of Policy and Practice in Intellectual Disabilities, 2, no. 1 (2005): 63-65. | Exclude | Presents results on out-of-home placement versus institutional placement. No information on patient outcomes. |
| Heller (2012) | Self-directed Support: Impact of Hiring Practices on Adults with Intellectual and Developmental Disabilities and Families. | American Journal on Intellectual & Developmental Disabilities, 117, no. 6 (2012): 464-477. | Exclude | This study compares outcomes based on who is giving care (e.g. family member, agency worker, friend). Does not evaluate outcome of personal budget. |
| Heller (1999) | Impact of a consumer-directed family support program on adults with developmental disabilities and their family caregivers. | Family Relations (1999): 419-427. | Exclude | Mid-point (year 4) of a study reported in Caldwell (2007) |
| Highland (2020) | Impact of Behavioral Health Homes on Cost and Utilization Outcomes. | Psychiatric Services, 71, no. 8 (2020): 796-802. | Exclude | Study about self-directed support in "behavioural health homes". No |

| | | | | |
|---|---|---|---|---|
| | | | | payment/budget paid to those in the self-directed cohort. |
| Hoolahan (2012) | IFS in action: personalising block contracts: as research report | The Centre for Welfare Reform | Exclude | Does not include any results, only plans and predicted total savings |
| Howard (2015) | 'I'm not really sure but I hope it's better': Early thoughts of parents and carers in a regional trial site for the Australian National Disability Insurance Scheme. | Disability & Society, 30, no. 9 (2015): 1365-1381. | Exclude | Qualitative results from interviews with users of NDIS in Australia. |
| HSRI (2019) | Making Self direction a reality: Using Individual Budgets to Promote Choice, Control, and Equity | Human Services Research Institute | Exclude | Overview of community-based services across the US. No data. |
| Iezzoni (2018) | Description of YESHealth: A consumer-directed intervention in a randomized trial of methods to improve quality of care for persons with disability. | Disability and health journal, 11, no. 4 (2018): 545-554. | Exclude | Description of a consumer-directed health intervention with no individualised payments (except a EUR10 recruitment bonus) |
| In Control (2010) | Your support, your way: the story so far of self directed support in the London Borough of Richmond upon Thames | | Exclude | A small scale evaluation of 19 personal budget holders in London (some additional QOL survey outcomes for 90 people). Data presented graphically on bar charts, no control for comparison). No possibility to separate older people from younger people. |
| Ismail (2017) | Do Personal Budgets Increase the Risk of Abuse? Evidence from English National Data | Journal of Social Policy, 46, no. 2 (2017): 291-311. | Exclude | UK study on an association between personal budget use and a referral for safeguarding due to possible abuse. The data presented is not relevant for this review. |
| JAG (2006) | Ten years with personal assistance | JAG | Exclude | Qualitative results from survey in Sweden |

| | | | | |
|---|---|---|---|---|
| JAG (2013) | The price of freedom of choice, self-dtermination and integrity | JAG | Exclude | The data provided is for 4 group homes with 4-5 residents, and presents the amount of personal assistance that would be possible to purchase for the current costs. |
| Jenkins (2010) | Journeying towards personalisation: from pilot to implementation: the learning and experiences of introducing self-directed support in Cambridgeshire | Journal of Care Services Management, 4, no. 3 (2010): 236-249. | Exclude | A somewhat personalised description of the implementation of personal budgets in Cambridge. No original results are provided. |
| Jones (2018) | Personal health budgets: Targeting of support and the service provider landscape | Working Paper 2948. Canterbury: Personal Social Services Research Unit, University of Kent, 2018. | Exclude | Describes the opinions of service providers on how personal budgets are faring in the UK. |
| Jones (2011) | The Cost of Implementing Personal Health Budgets | Department of Health | Exclude | This is an interim report, reporting on implementation costs. It is not broken down by health condition (only the mental health cohort is eligible for this review). |
| Jones (2012) | The impact of individual budgets on the targeting of support: findings from a national evaluation of pilot projects in England | Public Money and Management, 32, no. 6 (2012): 417-424. | Exclude | Reports and discusses the costs data from the Glendinning (2008) report. Nothing extra that is relevant for our review. |
| Jones (2013) | Personalization in the health care system: Do personal health budgets have an impact on outcomes and cost? | Journal of Health Services Research & Policy, 18, no. 2_suppl (2013): 59-67. | Exclude | Reports on findings already published in the Forder (2012) Personal Health Budgets report. |
| Jones (2010) | The costs of change: A case study of the process of implementing individual budgets across pilot local authorities in England | Health and Social Care in the Community, 18, no. 1 (2010): 51-58. | Exclude | Presents some estimates at the national level that are not relevant for our review |
| Jones (2018) | Personal Health Budgets : Implementation following the | | Exclude | 5 page summary document of the Personal Health Budget program reported in Forder (2012) |

| | | | | |
|---|---|---|---|---|
| | national pilot programme ; overall project summary | | | |
| Junne (2014) | The risk of users' choice: exploring the case of direct payments in German social care. | Health, Risk & Society, 16, no. 7-8 (2014): 631-648. | Exclude | Results from qualitative interviews with service users. |
| Kelly (2020) | Emergent Issues in Directly-Funded Care: Canadian Perspectives | Journal of Aging and Social Policy, (2020): 1-21. | Exclude | Qualitative study of problems with personal budgtes in Canada. |
| Kelly (2010) | Making a big difference: introducing individual budgets and self-directed support to disabled children and their families in Middlesbrough | In Control | Exclude | Some basic data presented on bar charts (no figures) on families' experience with personal budgets. |
| Kendrick (2017) | Australia's national disability insurance scheme: looking back to shape the future | Disability and Society, 32, no. 9 (2017): 1333-1350. | Exclude | Presents reflections on how disability policy has changed in Australia with the introduction of the NDIS |
| Keogh (2018) | Independent living: an evaluation of the Aiseanna Tacaiochta model of direct payments | Centre for Disability Law & Policy, Institute for Lifecourse & Society, National University of Ireland, Galway, Ireland | Exclude | No individual level data, no details on gender, age, type of disability of the 18 participants. |
| Kettunen (2019) | Feasibility of economic evaluation of personal budgets in Finland and preliminary evaluation plan | Diaconia University of Applied Sciences | Exclude | This outlines plans for an economic evaluation of the Finnish personal budget system. It does not present any findings to date. |
| Knapp (2014) | Investing in recovery: making the business case for effective interventions for people with schizophrenia and psychosis | The London School of Economics and Political Science, Centre for Mental Health | Exclude | General discussion on the topic of interventions for psychosis, with limited discussion on personal budgets. |
| KPMG (2012) | Evaluation of the consumer - directed care initiative Final Report | Department of Health and Ageing | Exclude | Initiative for older people only |
| KPMG (2012) | Evaluation of the consumer-directed care initiative – Appendices | Department of Health and Ageing | Exclude | Appendices to excluded article above |

| | | | | |
|---|---|---|---|---|
| Kremer (2006) | Consumers in charge of care: The Dutch personal budget and its impact on the market, professionals and the family | European Societies, 8, no. 3 (2006): 385-401. | Exclude | Overview of the Dutch personal budget system |
| Lakhani (2016) | Perspectives of self-direction: a systematic review of key areas contributing to service users' engagement and choice-making in self-directed disability services and supports | Health & Social Care in the Community, 26, no. 3 (2018): 295-313. | Exclude | Literature review on self-direction |
| Lancaster University (2017) | Personal Outcomes Evaluation Tool (POET) for adults in receipt of social care support: 2017 report | In Control | Exclude | Uncontrolled cross-sectional survey. No baseline data to compare with, just people's own perception of if things have improved or not. |
| Laragy (2015) | Australia's Individualised Disability Funding Packages: When Do They Provide Greater Choice and Opportunity? | Asian Social Work and Policy Review, 9, no. 3 (2015): 282-292. | Exclude | Qualitative results of interviews with people involved in individualised funding in Australia. |
| Laragy (2011) | Towards a framework for implementing individual funding based on an Australian case study. | Journal of Policy and Practice in Intellectual Disabilities, 8, no. 1 (2011): 18-27. | Exclude | Qualitative results from a case study of a single disability agency in Australia using individual funding. |
| Larsen (2013) | Implementing personalisation for people with mental health problems: A comparative case study of four local authorities in England | Journal of Mental Health, 22, no. 2 (2013): 174-182. | Exclude | Qualitative results of a survey of 4 English local authorities involved in personalisation. |
| Larsen (2015) | Outcomes from personal budgets in mental health: service users' experiences in three English local authorities. | Journal of Mental Health, 24, no. 4 (2015): 219-224. | Exclude | Qualitative results from interviews with 47 budget holders in 3 English local authority regions. |

| | | | | |
|---|---|---|---|---|
| Lawson (2010) | Finding our way | In Control | Exclude | Data provider on bar charts for user outcomes, sample of 100, but some data from an earlier study. Data include people aged 18-97 with no possibility to remove older people. Outcomes measured as improved/same/worse |
| Leece (2006) | Direct payments: creating a two-tiered system in social care? | British Journal of Social Work,36, no. 8 (2006): 1379-1393. | Exclude | The study uses administrative data to examine the financial situation of 480 people - 80 direct payment recipients, 13 over the age of 60. The study only looks at how their financial situation (income and savings) varies and not on health or cost outcomes. |
| Leece (2000) | It's a matter of choice: making direct payments work in Staffordshire | Practice: Social Work in Action, 12, no. 4 (2000): 37-48. | Exclude | Qualitative results from a survey of direct payment users |
| Leece (2003) | Money matters: an evaluation of the direct payment pilot project for parents of disabled children in Staffordshire | Journal of Integrated Care | Exclude | Qualitative results from a survey of parents of children receiving a direct payment |
| Leece (2010) | Developing new understandings of independence and autonomy in the personalised relationship | British Journal of Social Work, 40, no. 6 (2010): 1847-1865. | Exclude | Qualitative results from a study of relationships between employers and support providers |
| Lenehan (2008) | Individual budgets and direct payments: Issues, challenges and future implications for the strategic management of SEN: Policy paper 3, 6th series, April 2008: Chapter 2: Individual budgets and direct payments for children, young people and families. | Journal of Research in Special Educational Needs, 8, no. 3 (2008): 169-171. | Exclude | A descriptive study of what young people want from personal budgets |

| | | | | |
|---|---|---|---|---|
| Lindeman (2009) | Emerging tensions in the use of assessment tools in home and community care | Practice Reflexions Vol. 4 No. 1 2009 41 | Exclude | Discussion of assessment tools. Not directly personal budget related |
| Lombe (2008) | Exploring effects of institutional characteristics on saving outcome: the case of the cash and counseling program. | Journal of Policy Practice, 7, no. 4 (2008): 260-279. | Exclude | Study on how access to the cash and counseling programme allowed users to save for future purchases (no outcomes on how their lives were improve), an unintended characteristic of the programme. |
| Lombe (2016) | Understanding Effects of Flexible Spending Accounts on People with Disabilities: The Case of a Consumer-Directed Care Program. | Journal of Social Work in Disability & Rehabilitation, 15, no. 1 (2016): 62-75. | Exclude | Study on how access to the cash and counseling programme allowed users to save for future purchases (no outcomes on how their lives were improve), an unintended characteristic of the programme |
| Lord (2008) | Individualized funding in Ontario: Report of a Provincial Study. | Journal on Developmental Disabilities, 14, no. 2 (2008): 44. | Exclude | Only provides qualitative data on peoples outcomes and experiences. |
| Loughlin (2004) | Preferences for a cash option versus traditional services for Florida children and adolescents with developmental disabilities. | Journal of Disability Policy Studies, 14, no. 4 (2004): 229-240. | Exclude | Study on the level of interest in cash payments, and also preferences for they system characteristics |
| Lysaght (2015) | International Employment Statistics for People With Intellectual Disability-The Case for Common Metrics | Journal of Policy and Practice in Intellectual Disabilities, 12, no. 2 (2015): 112-119. | Exclude | Nothing to do with individual budgets |
| Maglajlic (2000) | Making direct payments a choice: a report on the research findings | Disability and Society, (2000): 99-113. | Exclude | A study on people preferences for personal budgets and their uses. |
| Mahoney (2004) | Determining Personal Care Consumers' Prefrences for a Consumer-Directed Cash and Counseling Option: Survey Results from Arkansas, Florida, New | Health Services Research, 39, no. 3 (2004): 643-664. | Exclude | A study to gauge interest in cash payment options |

| | | | | |
|---|---|---|---|---|
| | Jersey, and New York Elders and Adults with Physical Disabilities | | | |
| Mahoney (2006) | Cash and counseling: a promising option for consumer direction of home- and community- based services and supports. | Care Management Journals, 7, no. 4 (2006): 199-204. | Exclude | Descriptive overview of personal budget programs in the US |
| Mahoney (2002) | Consumer preferences for a cash option versus traditional services: telephone survey results from New Jersey elders and adults. | Journal of Disability Policy Studies, 13, no. 2 (2002): 75-87. | Exclude | Results of a survey on users' preferences and interest in the cash and counseling program in the US. |
| Manthorpe (2011) | Keeping it in the family? People with learning disabilities and families employing their own care and support workers: findings from a scoping review of the literature. | Journal of Intellectual Disabilities, 15, no. 3 (2011): 195-207. | Exclude | literature review |
| Manthorpe (2015) | Embarking on self-directed support in Scotland: a focused scoping review of the literature | European Journal of Social Work, 18, no. 1 (2015): 36-50. | Exclude | literature review |
| Marcenko (1994) | How families of children with severe disabilities choose to allocate a cash subsidy. | Journal of Mental Health Administration, 21, no. 3 (1994): 253-261. | Exclude | The subsidy doesn't replace the existing supports that families have. It investigates if receipt of the subsidy results in a reduction in the use of traditional services. . |
| Mavromaras (2018) | Evaluation of the NDIS | | Exclude | Evaluation of the Australian NDIS. Study population not clearly defined in terms of disability profiles, age, gender. Timeline of survey varied significantly (Wave one ran from end 2014, to second half of 2016.) Results presented in table and/or bar chart form, oftentimes quoting unpublished results. |

| | | | | |
|---|---|---|---|---|
| McDermid (2015) | Evaluation of the made to measure pilot: pooling personal budgets: final evaluation report | Loughborough University | Exclude | Study on the pooling of personal budgets received by family with disabled children. No quantitative outcome measures given. |
| McNeill (2017) | Use of Direct Payments in Providing Care and Support to Children with Disabilities: Opportunities and Concerns | British Journal of Social Work, 47, no. 7 (2017): 1903-1922. | Exclude | Study on the pooling of personal budgets received by family with disabled children. No quantitative outcome measures given. |
| Meng (2009) | Impact of a disease management-health promotion nurse intervention on personal assistance use and expenditures. | Home Health Care Services Quarterly, 28, no. 4 (2009): 113-129. | Exclude | No personal budget is paid to study participants, the intervention is provided by a nurse |
| Meng (2006) | Effect of a voucher benefit on the demand for paid personal assistance. | The Gerontologist, 46, no. 2 (2006): 183-192. | Exclude | The study includes people over 64 with a function disability. |
| Meng (2010) | Choice of personal assistance services providers by medicare beneficiaries using a consumer-directed benefit: rural-urban differences. | The Journal of rural health, 26, no. 4 (2010): 392-401. | Exclude | It looks at the impact of rural/urban on the choice of an agency or independent provider, it doesn't look at actual outcomes. |
| Mental Health Foundation (2015) | Your way: an evaluation of a model of community mental health support developed by Together for Mental Wellbeing | Mental Health Foundation | Exclude | This article presents a personalised support service for people with mental health conditions. The service can be used by people with individual budgets, but not exclusively. |
| Meyer (2005) | Das personengebundene Budget bei Pflegebedürftigkeit: Systemwechsel eröffnet Chancen für Anbieter und Nachfrager. | Kohlhammer; 2005. | Exclude | This is an interesting overview article, providing a discussion of experiences with personal budgeting in Germany and the potential and actual challenges it may pose. It is not a proper evaluation though, hence exclude. |
| Miller (2017) | Person-centered planning in mental health: A transatlantic collaboration to tackle implementation barriers. | American Journal of Psychiatric Rehabilitation, 20, no. 3 (2017): 251-267. | Exclude | Review/discussion article on person centred approaches |

| | | | | |
|---|---|---|---|---|
| Mitchell (2012) | Self-directed support and disabled young people in transition (part 2) | Journal of Integrated Care | Exclude | Review of self-directed support for young adults in Scotland |
| Mitchell (2012) | Self-directed support and disabled young people in transition (part 1) | Journal of Integrated Care | Exclude | Review of self-directed support for young adults in Scotland |
| Moran (2012) | Personalisation and carers: whose rights? Whose benefits? | British Journal of Social Work, 42, no. 3 (2012): 461-479. | Exclude | Reports findings from the carers evaluation in Glendinning (2009). No new data. |
| Moseley (2005) | Individual budgeting in state-financed developmental disabilities services in the United States | Journal of Intellectual and Developmental Disability, 30, no. 3 (2005): 165-170. | Exclude | Overview of individual budgets in different US states, no evaluations or outcome results |
| National Disability Insurance Scheme. (2018) | NDIS Participant Outcomes | Australian Government | Exclude | Uncontrolled cross-sectional survey to assess progress of participants. No comparison with a control group, or before entry into NDIS |
| National Disability Authority (2011) | The Introduction of Individual Budgets as a Resource Allocation System for Disability Services in Ireland | National Dsiability Authority, Ireland | Exclude | Discussion paper on the potential introduction of personal budgets in Ireland |
| National Disability Authority (2018) | Towards Personalised Budgets for People with a Disability in Ireland | Department of Health, Ireland | Exclude | A report from the task force on personal budgets in Ireland. It sets out proposed models for implementation. Provides some overviews of systems in other countries. |
| NDIS (2016) | WA NDIS Trials Evaluation: Final Report | NDIS | Exclude | Survey of 21 people involved in the trial, outcomes data sparse, only 1 page devoted to reporting participants outcomes. |
| Needhan (2018) | 'Any one of us could be among that number': Comparing the Policy Narratives for Individualized Disability Funding in Australia and England | Social Policy and Administration,52, no. 3 (2018): 731-749. | Exclude | Comparing the personal budgeting systems in the UK and Australia |

| | | | | |
|---|---|---|---|---|
| Netten (2012) | Personalisation through individual budgets: Does it work and for whom? | British Journal of Social Work, 42, no. 8 (2012): 1556-1573. | Exclude | Reports and discusses the outcomes data from the Glendinning (2008) final report. No new data presented. |
| Norrie (2014) | Early experiences in extending personal budgets in one local authority | Working With Older People | Exclude | Qualitative results from interviews with older people and those with mental health problems. |
| O'Brien (2005) | Person centered funding: using vouchers and personal budgets to support recovery and employment for people with psychiatric disabilities. | Journal of Vocational Rehabilitation, 23, no. 2 (2005): 71-79. | Exclude | This is an article discussing the implementation of two schemes for vouchers and personal budgets in the US, no data provided. |
| Orion (2007) | Evaluation of disability support program pilot project | Orion Marketing Research | Exclude | The focus of the study is on the development of personalised plans for disabled adults using existing service structures, not on the delivery and use of a personal budget. |
| Ottmann (2009) | Experiences of disability consumer-directed care users in Australia: results from a longitudinal qualitative study. | Health & Social Care in the Community, 17, no. 5 (2009): 466-475. | Exclude | Qualitative results from a study of 12 families receiving consumer directed care. |
| Peak (2009) | My budget my choice: implementing self-directed support in the City of London, October 2008 | In Control | Exclude | Data presented on bar charts for basic questions regarding satisfaction with services. No data on the 10 participants regarding age or disability. |
| Perkins (2014) | Circles of support and personalisation: exploring the economic case | Journal of Intellectual disabilities | Exclude | A study of circles of support, where a "circle" of carers and family help a disable person to plan their care. Not specifically focused on personal budgets. |
| Phillips (2007) | Commonalities and Variations in the Cash and Counseling Programs | Health Services Research, 42, no. 1p2 (2007): 397-413. | Exclude | Only describes the programmes, provides no outcomes. |

| | | | | |
|---|---|---|---|---|
| | across the Three Demonstration States. | | | |
| Phillips (2002) | Moving to independent choices: the implementation of the Cash and Counseling demonstration in Arkansas | Mathematica Policy Research Inc. | Exclude | Linked to the Cash and Counseling demonstration but provides no outcomes data. |
| Phillips (2003) | Enabling personal preference: the implementation of the Cash and Counseling Demonstration in New Jersey: final report | Mathematica Policy Research Inc. | Exclude | No outcomes data, only implementation details |
| Phillips (2003) | Lessons from the implementation of cash and couseling in Arkansas, New Jersey and Florida | Mathematica Policy Research Inc. | Exclude | No outcomes data, only implementation details |
| Phillips (2004) | Changing to consumer directed care: the implementation of the cash and counseling demonstration in Florida | Mathematica Policy Research Inc. | Exclude | No outcomes data, only implementation details |
| Pike (2016) | Individualised budgeting for social care services for people with a disability: International approaches and evidence on financial sustainability | Health Research Board, Dublin | Exclude | Review of systems in other countries |
| Pitts (2008) | Doing it your way: the story of self-directed support in Worcestershire | In Control | Exclude | Some basic data presented on bar charts on staff experiences and what users spent their money on. |
| Prabhakar (2008) | Individual budgets for families with disabled children: scoping study: final case study report | | Exclude | Provides details on the pilot sites, before the pilot started, for the pilot of individual budgets for families with disabled children reported in Johnson (2011) |
| Prabhakar (2008) | Individual budgets for families with disabled children: scoping study: literature review report | | Exclude | Background discussion to the pilot of individual budgets for families with disabled children reported in Johnson (2011) |

| | | | | |
|---|---|---|---|---|
| Prabhakar (2008) | Individual budgets for families with disabled children: scoping study | | Exclude | Background discussion to the pilot of individual budgets for families with disabled children reported in Johnson (2011) |
| Prabhakar (2012) | Evaluation of the extended individual budget programme for families with disabled children: the extended packages | Department for Education | Exclude | Details the organisational and logistic details of extending the programme for 12 months. No outcomes data. Outcomes of the extended packages are reported in Johnson (2012) |
| Prabhakar (2010) | Individual budgets for families with disabled children: interim report 2010 | Department for Education | Exclude | Reports baseline data and participant characteristics. Final data reported in Johnson (2011) |
| Priestley (2007) | Direct payments and disabled people in the UK: supply, demand and devolution | British Journal of Social Work, 37, no. 7 (2007): 1189-1204. | Exclude | Overview of personal budgets in England, Scotland and Wales |
| Productivity Commission (2011) | Disability Care and Support | Productivity Commission Inquiry Report Volume 1, No. 54, 31 July 2011 | Exclude | This report provides estimates on the future cost of the NDIS, but no actual evaluation data as it was written before the rollout. Vol 1 and Vol 2 |
| Productivity Commission (2017) | Productivity Commission Study Report National Disability Insurance Scheme (NDIS) Costs | Australian Government | Exclude | Provides cost estimates and projects future costs. Provides costs of individual packages of support, but no comparison to a traditional system and no clear details on sample sizes, ages, disabilities etc. |
| Purcal (2016) | Direct Funding Trial : Final evaluation report | | Exclude | A very small study reporting for both intervention and treatment groups. But no direct comparison is made between groups and no standard deviations are reported to allow for comparison. |
| Quach (2010) | Supporting people with disabilities in managing individual budgets: the role of support brokers. | Professional Case Management, 15, no. 1 (2010): 29-37. | Exclude | Provides a description of the role of support brokers during the Massachusetts trial of individual budgets. No details on outcomes for service users. |

| | | | | |
|---|---|---|---|---|
| Rabiee (2009) | Individual Budgets: Lessons from Early Users' Experiences | British Journal of Social Work, 39, no. 5 (2009): 918-935. | Exclude | Qualitative results from interviews with 14 personal budget users |
| Richards (2008) | This time it's personal: making self-directed support a reality for people with learning disabilities in Northamptonshire | Northamptonshire County Council | Exclude | A small scale study of 12 budget holders, data presented on a single bar chart where user rates their outcomes as better/same. |
| Riddell (2006) | Disabled people and direct payments: a UK comparative study | *ESRC Award RES-000-23* 263 (2006). | Exclude | Comparison of personal budget systems in the UK |
| Ridley (2011) | Evaluation of self-directed support test sites in Scotland | The Scottish Government, 2011. | Exclude | Evaluation of Scottish self-direction, only qualitative study undertaken |
| Ridley (2003) | Direct what? The untapped potential of direct payments to mental health service users. | Disability & Society, 18, no. 5 (2003): 643-658. | Exclude | Qualitative study of direct payment in Scotland |
| Riley (2015) | Cumulative expenditures under the DI, SSI, Medicare, and Medicaid programs for a cohort of disabled working-age adults. | Health services research, 50, no. 2 (2015): 514-536. | Exclude | Not related to personal budgets |
| Robertson (2005) | The impact of person centred planning | Institute for Health Research, Lancaster University | Exclude | Focus on person centred planning, not personal budgets or payment |
| Rosenberg (2005) | Consumer Directed Support : Lessons Learned from Wisconsin ' s Family Care Program | Department of Health and Family Services/Pathways to Independence | Exclude | Qualitative results from interviews with staff and managers at family care institutes for older people |
| San Antonio (2010) | Lessons from the Arkansas Cash and Counseling program: how the experiences of diverse older consumers and their caregivers address family policy concerns. | Journal of Aging & Social Policy, 22, no. 1 (2009): 1-17. | Exclude | Qualitative results from the cash and counselling evaluation |
| Schore (2007) | Consumer Enrollment and Experiences in the Cash and Counseling Program. | Health Services Research, 42, no. 1p2 (2007): 446-466. | Exclude | Contains data on how budgets were used and how long users waited to receive their payment. Only discussion included (with references) on user satisfaction. |

| | | | | |
|---|---|---|---|---|
| Schore (2004) | Consumer and counselor experiences in the Arkansas independent choices program | Mathematica Policy Research Inc. | Exclude | Reports on the Cash and Counseling demonstration. Only presents data for the treatment group on process issues and counsellors/consultants |
| Scourfield (2005) | Implementing the Community Care (Direct Payments) Act: will the supply of personal assistants meet the demand and at what price? | Journal of Social Policy, 34, no. 3 (2005): 469-488. | Exclude | Discussion focused on the logistics of implementing direct payments |
| Shen (2008) | Does mental illness affect consumer direction of community-based care? Lessons from the Arkansas Cash and Counseling program. | The Gerontologist,48, no. 1 (2008): 93-104. | Exclude | The focus is solely on elderly people in the Cash and Counseling demonstration |
| Sikma (2003) | Nurse Delegation in Washington State: A Case Study of Concurrent Policy Implementation and Evaluation | Policy, Politics, & Nursing Practice, 4, no. 1 (2003): 53-61. | Exclude | Discussion on polices for changing nursing practices |
| Sim (2016) | Future evaluation of the Integrated Personal Commissioning programme: Mapping the logic and assessing evaluability | RAND, Europe | Exclude | Present a plan to evaluation the IPC programme, but no outputs. |
| Simon-Rusinowitz (1997) | Determining consumer preferences for a cash option: Arkansas survey results. | Health care financing review, 19, no. 2 (1997): 73. | Exclude | Study undertaken in advance of the Cash and Counseling demonstration to gauge potential interest of service users. |
| Simon-Rusinowitz (2001) | Consumer and surrogate preferences for a cash option versus traditional services: Florida adults with developmental disabilities. | Mental retardation, 39, no. 2 (2001): 87-103. | Exclude | Survey to gauge interest in a cash option |
| Simon-Rusinowitz (2005) | Paying family caregivers: An effective policy option in the Arkansas Cash and Counseling Demonstration and Evaluation. | Marriage & Family Review 37, no. 1-2 (2005): 83-105. | Exclude | The majority of participants (64%) are over 65, with no way to remove their results. This study doesn't compare a personal budget to either a control group or a pre-budget time. |

| | | | | |
|---|---|---|---|---|
| Sims (2014) | A scoping review of personalisation in the UK: approaches to social work and people with learning disabilities. | Health & social care in the community, 22, no. 1 (2014): 13-21. | Exclude | A review of personalisation in the UK |
| Slasberg (2012) | Can personal budgets really deliver better outcome for all at no cost? Reviewing the evidence, costs and quality | Disability and Society, 27, no. 7 (2012): 1029-1034. | Exclude | Review of the costs of personal budgets |
| Smith (2014) | Evaluation of the Special Educational Needs and Disability (SEND) Pathfinder Programme: impact research report: qualitative research with families (second cohort): research report | Department for Education | Exclude | Qualitative evaluation of the pathfinder programme |
| Snethen (2016) | Exploring Personal Medicine as Part of Self-Directed Care: Expanding Perspectives on Medical Necessity. | Psychiatric Services, 67, no. 8 (2016): 883-889. | Exclude | Study describing the services/objects requested by self-direction users. |
| Spalding (2006) | Self Managed Care Programs in Canada : A Report to Health Canada Self Managed Care Programs in Canada | A report to Health Canada. Health Care Policy Directorate, Health Canada, 2006. | Exclude | A descriptive review of self-managed home care programs in Canada |
| Spall (2005) | Fixing the system? The experience of service users of the quasi-market in disability services in Australia. | Health & social care in the community, 13, no. 1 (2005): 56-63. | Exclude | Qualitative results from individual funding in Australia |
| Spandler (2004) | Direct payments, independent living and mental health: an evaluation | HASCAS | Exclude | Report on direct payments for people with mental health problems, only presents qualitative data. |
| Spandler (2005) | Enabling access to direct payments: an exploration of care co-ordinators decision-making practices | Journal of Mental Health, 14, no. 2 (2005): 145-155. | Exclude | Describes care-coordinators experiences with direct payments |

| | | | | |
|---|---|---|---|---|
| Spandler (2006) | Opportunities for independent living using direct payments in mental health | Health and Social Care in the Community, 14, no. 2 (2006): 107-115. | Exclude | Qualitative results from interviews with 27 direct payment recipients. |
| Spaulding-Givens (2019) | Money matters: participants' purchasing experiences in a budget authority model of self-directed care. | Social Work in Mental Health, 17, no. 3 (2019): 323-343. | Exclude | Qualitative study of 17 self-directed care participants and their purchases |
| Spaulding-Givens (2011) | Florida self-directed care: An exploratory study of participants' characteristics, goals, service utilization, and outcomes. | Dissertation Abstracts International Section A: Humanities and Social Sciences | Exclude | This study provides data on outcomes and expenditure for self-directing care participants. But it does not compare the outcomes to a no-budget care (control or pre-SDC) |
| Spaulding-Givens (2015) | Self-directed care: Participants' service utilization and outcomes. | Psychiatric Rehabilitation Journal | Exclude | This study provides data on outcomes and expenditure for self-directing care participants. But it does not compare the outcomes to a no-budget care (control or pre-SDC) (paper linked to Dissertation in 2012) |
| Spivack (2014) | Evaluation of the Special Educational Needs and Disability Pathfinder Programme: thematic report: collaborative working with social care: research report | | Exclude | Overview of the pathfinder evaluation, no outcomes data presented |
| Squillace (2002) | Personal assistance service choice and decision-making among persons with disabilities and surrogate representatives. | Journal of Mental Health and Aging, | Exclude | Study comparing service users opinions on their carer's performance with that of their surrogate/representative |
| Stainton (2004) | 'I have got my life back': Users' experience of direct payments. | Disability & Society, 19, no. 5 (2004): 443-454. | Exclude | Qualitative results of users experiences with direct payments |
| Stainton (2013) | A Comparison of Cost and Service Utilization Across Individualized and Traditional Funding Options | CENTRE FOR INCLUSION AND CITIZENSHIP | Exclude | There is a comparison with the total cost of traditional block funded contracts, but the groups are not strictly comparable, with |

| | | | | |
|---|---|---|---|---|
| | Through Community Living British Columbia | | | disability type, age, etc varying so this could account for differences. |
| Stainton (2009) | Independence pays: A cost and resource analysis of direct payments in two local authorities | Disability and Society, Disability & Society 24, no. 2 (2009): 161-172. | Exclude | This presents 4 examples of user costs based on estimates taken from making averages from the overall costs. |
| Stanhope (2019) | A Mixed Methods Study of Organizational Readiness for Change and Leadership During a Training Initiative Within Community Mental Health Clinics. | Administration and policy in mental health, 46, no. 5 (2019): 678-687. | Exclude | Results from interviews with staff from person centred care planning clinics, no direct evidence for personal budgets |
| Stanhope (2015) | Person-centered care planning and service engagement: a study protocol for a randomized controlled trial. | Trials, 16, no. 1 (2015): 1-11. | Exclude | Study on the effectiveness of person-centred planning, but no direct involvement of personal budgets |
| Stevens (2013) | Jobs First evaluation: final report | King College London | Exclude | Qualitative study of how personal budgets could help people to find a job |
| Stevens (2017) | Social work support for employment of people with learning disabilities: Findings from the English Jobs First demonstration sites. | Journal of Social Work, 17, no. 2 (2017): 167-185. | Exclude | Study of how personal budgets could help people to find a job |
| Steyn (2002) | The role of Disability Living Allowance in the management of attention-deficit/hyperactivity disorder. | Child: Care, Health & Development, 28, no. 6 (2002): 523-527. | Exclude | The allowance is for purchasing articles that make life easier, not for arranging or paying for care. The only data provided is on what was purchased with the allowance |
| Stone (2001) | Providing long-term care benefits in cash: moving to a disability model. | Health affairs, 20, no. 6 (2001): 96-108. | Exclude | Discussion paper on disability policies in the US |
| Synergia (2011) | Evaluation of Individualised Funding Following the expansion to new Host Providers | New Zealand Ministry of Health | Exclude | User outcomes collected through qualitative interviews. Includes financial analysis for "select users" of individual funding but there is no precise definition |

| | | | | |
|---|---|---|---|---|
| | | | | of the population being studied (age, gender, etc). |
| Tew (2015) | 'And the stuff that I'm able to achieve now is really amazing': The potential of personal budgets as a mechanism for supporting recovery in mental health. | British Journal of Social Work, 45, no. suppl_1 (2015): i79-i97. | Exclude | Qualitative interviews with 53 budget users. |
| Thom (2011) | Individual budgets for families with disabled children: resource allocation thematic | | Exclude | Discussion on resource allocation for families with disabled children |
| Thomas (2018) | Associations between the peer support relationship, service satisfaction and recovery-oriented outcomes: a correlational study. | Journal of Mental Health, 27, no. 4 (2018): 352-358. | Exclude | Study of the association between self-directed care outcomes and peer relationships with service providers. |
| Thomas (2014) | The Medicaid medically improved group: losing disability status and growing earnings. | Medicare & Medicaid research review, 4, no. 1 (2014). | Exclude | Study of employment outcomes, not personal budget specific |
| Timonen (2006) | Care revolutions in the making? A comparison of cash-for-care programmes in four European countries | Ageing and Society, 26 (2006): 455. | Exclude | Review of programmes aimed at the elderly |
| Tu (2013) | Evaluation of the Right to Control Trailblazers: synthesis report | | Exclude | Report from the Right to Control project in the UK. Not directly a project about personal budgets. It looks into how users can control their care betters, including aspects such as housing, employment, etc. |
| Ungerson (1999) | Personal assistants and disabled people: an examination of a hybrid form of work and care | Work Employment and Society, 13, no. 4 (1999): 583-600. | Exclude | Qualitative reports on the relationship between personal assistants and disabled people |
| Van Ginneken (2012) | Personal healthcare budgets: What can England learn from the Netherlands? | BMJ (Online), 344 (2012). | Exclude | A discussion on personal budgets in England |

| | | | | |
|---|---|---|---|---|
| Wallack (2002) | Short- and intermediate-term trends affecting Medicaid policy for persons with disability, chronic illness, and special needs. | Journal of Disability Policy Studies, 12, no. 4 (2002): 236-242. | Exclude | Discussion of policy trends in the US |
| Waters (2014) | Third National Personal Budget Survey | In Control | Exclude | Uncontrolled cross-sectional survey. No control or before-after comparison. |
| Waters (2009) | Steering my own course: the introduction of self-directed support in Cambridgeshire | In Control | Exclude | Basic data provided on bar charts, regarding what budgets were used for and rating health and control as better/same/worse |
| Watts (2014) | The use of personal budgets for employment support | Bath: National Development Team for Inclusion (2014). | Exclude | A study on how people use personal budgets to gain employment |
| Weathers (2010) | Expanding access to health care for Social Security Disability Insurance beneficiaries: early findings from the accelerated benefits demonstration. | Social security bulletin, 70 (2010): 25. | Exclude | Study on providing medical benefits to disabled people while they wait for their social benefits package |
| Webber (2014) | The effectiveness of personal budgets for people with mental health problems: a systematic review. | Journal of Mental Health, 23, no. 3 (2014): 146-155. | Exclude | Review of personal budgets |
| Whitaker (2015) | Personalisation in children's social work: an ethnographic study of practice in England | CASCADE<br>Research Briefing | Exclude | A short overview of the individual budgets for families programme |
| Whitaker (2015) | Personalisation in children's social work: from family support to "the child's budget". | Journal of Integrated Care, | Exclude | Qualitative findings from study of individual funding for children |
| Williams (2013) | An evaluation of a person-centred care programme for long-term care facilities | Ageing and Society, 35, no. 3 (2015): 457. | Exclude | An evaluation of person-centred care, no direct payment of budget involved. |
| Williams (2011) | Your life, your choice: qualitative research carried out as part of the | Office for Disability Issues, 2011. | Exclude | Qualitative study on support planning from user led organisations. |

| | | | | |
|---|---|---|---|---|
| | 'Support planning and brokerage' initiative | | | |
| Williams (2014) | Your life, your choice: Support planning led by disabled people's organisations. | British Journal of Social Work, 44, no. 5 (2014): 1197-1215. | Exclude | Qualitative study on support planning from user led organisations. |
| WinKlusion (2019) | Formation inclusive - de facilitateur et facilitatrice en planification personnalisee de l'avenir | | Exclude | No quantitative data |
| Witcher (2000) | Direct payments: the impact on choice and control for disabled people | Scottish Executive Central Research Unit | Exclude | Interviews with local authorities in Scotland on the implementation of direct payments |
| Woittiez (2018) | An international comparison of care for people with intellectual disabilities. An exploration | The Netherlands Institute for Social Research. | Exclude | A comparison of the Dutch system with Flanders, England and Ontario for services for people with intellectual disabilities |
| Woolham (2016) | Do direct payments improve outcomes for older people who receive social care? Differences in outcome between people aged 75+ who have a managed personal budget or a direct payment | Ageing and Society, 37, no. 5 (2017): 961-984. | Exclude | A study of people over 75 receiving a personal budget |
| Woolham (2019) | The employment conditions of social care personal assistants in England | Journal of Adult Protection | Exclude | Description of the employment condition of personal assistants |
| Young (2003) | Self-Directed Care: An Evaluation | Policy, Politics & Nursing Practice, 4, no. 3 (2003): 185-195. | Exclude | Qualitative evaluation of self-directed care in the US |
| Cook (2008) | A self-directed care model for mental health recovery. | Psychiatric Services | failed quality assessment | |
| Fisher (2008) | Attendant Care Program Direct Funding Pilot Evaluation | Final Report for the NSW Department of Ageing, Disability and Home | failed quality assessment | |

| | | | | |
|---|---|---|---|---|
| Johnson (2012) | Evaluation of the extended individual budget programme for families with disabled children: the family journey one year on | | failed quality assessment | |
| Johnson (2011) | Individual budgets for families with disabled children: final evaluation report: the family journey | | failed quality assessment | |
| Kim (2006) | Comparing outcomes of persons choosing consumer-directed or agency-directed personal assistance services. | Journal of Rehabilitation | failed quality assessment | |
| Prabhakar (2012) | Extended evaluation of the individual budget programme for families with disabled children: technical annex | | failed quality assessment | |
| Prabhakar (2011) | Individual budgets for families with disabled children: final evaluation report: the IB process | | failed quality assessment | |
| Thom (2019) | Evaluation of the Integrated Personal Commissioning (IPC) Programme: final report | | failed quality assessment | |

| | | | | |
|---|---|---|---|---|
| Thom (2011) | Individual budgets for families with disabled children: final evaluation report: recommendations and implications | | failed quality assessment | Third of 3 reports from the evaluation of individual budgets for children with disabilities |
| Vinton (2010) | Caregivers' perceptions of a consumer-directed care program for adults with developmental disabilities. | Journal of Family Social Work | failed quality assessment | |
| Fleming-Castaldy (2008) | Consumer-directed personal care assistance and quality of life for persons with physical disabilities. | New York University, 2008. | no full text | This is a doctoral dissertation, which "examined the relationships between consumer-directed self-management of PCA, perceived control of and satisfaction with PCA service delivery, and QoL". |
| Fortune (2005) | Individual Budgets According to Individual Needs: The Wyoming DOORS System. | Journal of Intellectual Disability Research 48, no. 4-5 (2004). | no full text | This is a chapter from a book, "blending original research with policy analysis, critical reviews of existing knowledge, and examples of cutting-edge programs and policies, this book demonstrates what works and helps readers make sound decisions about how to allocate resources" |
| Henerson (2011) | Individual budgets for families with disabled children: final case study report: Gateshead pilot site | | no full text | Presents results from one pilot site of the individual budgets for families with disabled children program. |
| Herman (1991) | Use and impact of a cash subsidy program. | Mental retardation 29, no. 5 (1991): 253. | no full text | This is a study of a cash subsidy received by families with disabled children. Based on the abstract, this seems more related to what families used their cash subsidy for rather than relevant outcomes. |
| Hurstfield (2011) | Individual budgets for families with disabled children: final case study report: Coventry pilot site | | no full text | Presents results from one pilot site of the individual budgets for families with disabled children program. |

| | | | | |
|---|---|---|---|---|
| Leece (2008) | Direct payments and the experience of personal assistants | Community Care, 27.11.08, 2008, pp.32-33. | no full text | The abstract indicates that this small study reporting on personal assistant's outcomes, not users or unpaid caregivers. |
| McCrindle (2011) | Individual budgets for families with disabled children: final case study report: Newcastle pilot site | | no full text | Presents results from one pilot site of the individual budgets for families with disabled children program. |
| Mickel (2009) | Where to start with individual budgets? | Community Care, 8.1.09, 2009, pp.32-33 | no full text | This study looks at the experiences of staff and families involved in a small pilot of individual budgets for 8 people. |
| Moseley (2005) | Having it Your Way: A National Study of Individual Budgeting Practices within the States. | Costs and outcomes of community services for people with intellectual disabilities, (2005): 263-288. | no full text | Another chapter of the same book as Fortune (2005) above. |
| Office for Public Management (2010) | Delivering personal budgets for adult social care: reflections from Essex | | no full text | Reports on interviews with 46 older and disabled people in receipt of personal budgets. It is clear from the abstract that no comparison is provided with before-budget or a control group. |
| Parashar (2011) | Individual budgets for families with disabled children: final case study report: Derbyshire pilot site | | no full text | Presents results from one pilot site of the individual budgets for families with disabled children program. |
| Prabhakar (2011) | Individual budgets for families with disabled children: final technical report | | no full text | Technical report from the individual budgets for families with disabled children program. |
| Prabhakar (2011) | Individual budgets for families with disabled children: final case study report: Gloucestershire pilot site | | no full text | Presents results from one pilot site of the individual budgets for families with disabled children program. |
| Suther (1995) | The CLASS program: self-directed care. | Caring: National Association for Home Care magazine 14, no. 4 (1995): 72-75. | no full text | Reports on "issues surround self-directed care, and individuals who need habilitation services" |
| Turner (2011) | Individual budgets for families with disabled children: final case study report: Essex pilot site | | no full text | Presents results from one pilot site of the individual budgets for families with disabled children program. |

| | | | | |
|---|---|---|---|---|
| Weinbach (2010) | "We got utterly efficient encouragement to be able to succeed..." Criteria for the organisation of effective counselling on personal budget matters. | "Die haben uns sehr bestärkt in der Sache, dass wir das schaffen" Kriterien für die Gestaltung von guten Beratungsangeboten zum Persönlichen Budget." *Vierteljahresschrift für Heilpädagogik und ihre Nachbargebiete* 3 (2010): 212-223. | no full text | Reports on "a model project on budget assistance. The outcomes of the empirical research allow to define more precisely the function of this kind of counselling and support in the process of application and utilisation of personal assistance." |

Table S8. Quality assessment results by domain

| | | **Reporting (11)** | **External validity (3)** | **Internal validity (7)** | **Confounding / selection bias (6)** | **Power (1)** | **Score (28)** | **Quaity grading** |
|---|---|---|---|---|---|---|---|---|
| 1 | Carlson (2005) | 9 | 3 | 5 | 6 | 1 | 24 | Excellent |
| 2 | Brown (2007) | 8 | 3 | 5 | 6 | 1 | 23 | Good |
| 3 | Cook (2019) | 9 | 2 | 5 | 6 | 0 | 22 | Good |
| 4 | Dale (2005) | 8 | 1 | 5 | 6 | 1 | 21 | Good |
| 5 | Caldwell (2007) | 10 | 3 | 4 | 4 | 0 | 21 | Good |
| 6 | Glendinning (2008) | 8 | 1 | 5 | 6 | 1 | 21 | Good |
| 7 | Glendinning (2009) | 9 | 1 | 4 | 6 | 0 | 20 | Good |
| 8 | Forder (2012) | 9 | 1 | 5 | 4 | 1 | 20 | Good |
| 9 | Foster (2004) | 8 | 0 | 5 | 6 | 1 | 20 | Good |
| 10 | Foster (2003) | 7 | 1 | 5 | 6 | 1 | 20 | Good |
| 11 | Dale (2004) | 8 | 0 | 5 | 6 | 0 | 19 | Good |
| 12 | Croft (2019) | 7 | 3 | 5 | 3 | 0 | 18 | Fair |
| 13 | Pelizza et al. (2022) | 7 | 2 | 5 | 4 | 0 | 18 | Fair |
| 14 | Hagglund (2004) | 7 | 3 | 4 | 3 | 0 | 17 | Fair |
| 15 | Leuci et al. (2021) | 8 | 0 | 5 | 4 | 0 | 17 | Fair |
| 16 | Shen (2008) | 7 | 1 | 5 | 4 | 0 | 17 | Fair |
| 17 | Beatty (1998) | 7 | 2 | 4 | 3 | 0 | 16 | Fair |
| 18 | Benjamin (2000) | 8 | 2 | 4 | 2 | 0 | 16 | Fair |

| | | | | | | | | |
|---|---|---|---|---|---|---|---|---|
| 19 | Croft (2020) | 7 | 1 | 5 | 3 | 0 | 16 | Fair |
| 20 | Fontecedro (2020) | 7 | 2 | 4 | 2 | 0 | 15 | Fair |
| 21 | Woolham (2013) | 8 | 1 | 4 | 2 | 0 | 15 | Fair |
| 22 | Conroy (2002) | 6 | 2 | 4 | 2 | 0 | 14 | Fair |
| 23 | Wierner (2007) | 6 | 1 | 4 | 3 | 0 | 14 | Fair |
| 24 | Kim (2006) | 6 | 1 | 3 | 3 | 0 | 13 | Poor |
| 25 | Thom (2019) | 8 | 1 | 4 | 0 | 0 | 13 | Poor |
| 26 | Vinton (2010) | 7 | 1 | 4 | 1 | 0 | 13 | Poor |
| 27 | Jonhson (2011) | 5 | 1 | 3 | 3 | 0 | 12 | Poor |
| 28 | Cook (2008) | 5 | 1 | 4 | 2 | 0 | 12 | Poor |
| 29 | Fisher (2008) | 5 | 1 | 5 | 1 | 0 | 12 | Poor |
| 30 | Johnson (2012) | 4 | 1 | 3 | 2 | 0 | 10 | Poor |
| 31 | Prabhakar (2011) | 3 | 1 | 2 | 2 | 0 | 8 | Poor |
| 32 | Prabhakar (2012) | 3 | 1 | 2 | 2 | 0 | 8 | Poor |
| 33 | Thom (2011) | 4 | 1 | 2 | 2 | 9 | 9 | Poor |

Table S9. Detailed quality assessment using the Downs and Black Checklist

Note: *Item number 27 on the checklist was simplified to determine if a power analysis had been performed to determine sample size or not (Yes = 0, No = 0).*

| **Author (year):** Beatty (1998)<br>**Study Title:** Personal assistance for people with physical disabilities: consumer-direction and satisfaction with services<br>**Study Type:** controlled cross-sectional study | | | | | |
|---|---|---|---|---|---|
| | | Score | | | |
| | | 0 | 1 | 2 | Comment |
| **Reporting** | | | | | |
| 1. | Is the hypothesis/aim/objective of the study clearly described? | | Yes | | Objective is clearly stated at the start |
| 2. | Are the main outcomes to be measured clearly described in the Introduction or Methods sections? | | Yes | | Described in Abstract and Methods sections |
| 3. | Are the characteristics of the patients included in the study clearly described? | | Yes | | Age, gender, civil status, and race |
| 4. | Are the interventions of interest clearly described? | | Yes | | The PAS program and the situation of the control group are detailed in the Data section |
| 5. | Are the distributions of principal confounders in each group of subjects to be compared clearly described? | No | | | No specific mention of confounders. |
| 6. | Are the main findings of the study clearly described? | | Yes | | Clearly described in Results section and Table 2 |
| 7. | Does the study provide estimates of the random variability in the data for the main outcomes? | | Yes | | Standard deviation of main outcome score provided. |
| 8. | Have all important adverse events that may be a consequence of the intervention been reported? | No | | | No adverse effects mentioned |
| 9. | Have the characteristics of the patients lost to follow-up been described? | No | | | Not reported, or not relevant as this is a cross-sectional study |

| | | | | | |
|---|---|---|---|---|---|
| 10. | Have actual probability values been reported (e.g., 0.035 rather than <0.05) for the main outcomes except where the probability value is less than 0.001? | | Yes | | |
| | | | | | **Total reporting score: 7/11** |
| **External Validity** | | | | | |
| 11. | Were the subjects asked to participate in the study representative of the entire population from which they were recruited? | | Yes | | |
| 12. | Were those subjects who were prepared to participate representative of the entire population from which they were recruited? | Unable to determine | | | No details provided on the people who chose not to participate (they didn't return the consent form) ...no response rate given |
| 13. | Were the staff, places, and facilities where the patients were treated, representative of the treatment the majority of patients receive? | | Yes | | |
| | | | | | **Total external validity score: 2/3** |
| **Internal Validity – Bias** | | | | | |
| 14. | Was an attempt made to blind study participants to the intervention they have received? | No | | | |
| 15. | Was an attempt made to blind those measuring outcomes of the intervention? | No | | | |
| 16. | If any of the results of the study were based on "data dredging", was this made clear? | | Yes | | |
| 17. | In trials and cohort studies, do the analyses adjust for different lengths of follow-up of patients, or in case control studies, is the time period between the intervention and outcomes the same for cases and controls? | No | | | There is no information about the duration of participation in consumer-directed care |

| | | | | | |
|---|---|---|---|---|---|
| 18. | Were the statistical tests used to assess the main outcomes appropriate? | | Yes | | $\chi^2$ and *t* tests conducted |
| 19. | Was compliance with the intervention/s reliable? | | Yes | | No non-compliance reported |
| 20. | Were the main outcome measures used accurate (valid and reliable)? | | Yes | | Cronbach's alpha of 0.88 |
| | | | | | **Total bias score: 4/7** |
| **Internal Validity – Confounding (Selection bias)** | | | | | |
| 21. | Were the patients in different intervention groups (trials and cohort studies) or were the cases and controls (case-control studies) recruited from the same population? | | Yes | | |
| 22. | Were study subjects in different intervention groups (trial and cohort studies) or were the cases and controls (case-control studies) recruited over the same period of time? | Unable to determine | | | Study conducted over a 3 year period but there is no information on when participants were recruited |
| 23. | Were study subjects randomised to intervention groups? | No | | | |
| 24. | Was the randomised intervention assignment concealed from both patients and health care staff until recruitment was compete and irrevocable? | No | | | |
| 25. | Was there adequate adjustment for confounding in the analyses from which the main findings were drawn? | | Yes | | A separate analysis on satisfaction was conducted when controlling for costs |
| 26. | Were losses of patients to follow-up taken into account? | | Yes | | No follow up as this was a single survey |
| | | | | | **Total confounding score: 3/6** |
| **Power** | | | | | |
| 27. | *Did the study conduct a power analysis to determine the sample size needed to detect a significant* | | | | |

| | | | | | |
|---|---|---|---|---|---|
| | *difference in effect size for one or more outcome measures?* | | | | |
| **Total power score: 0/1** | | | | | |
| | | | | | |
| **Total quality score: 16/28** | | | | | |

------------------------------------------------------------------------

| **Author (year):** Benjamin (2000)<br>**Study Title:** Comparing consumer-directed and agency models for providing supportive services at home<br>**Study Type:** Controlled cross-sectional study | | | | | |
|---|---|---|---|---|---|
| | | Score | | | |
| | | 0 | 1 | 2 | Comment |
| **Reporting** | | | | | |
| 1. | Is the hypothesis/aim/objective of the study clearly described? | | Yes | | Objective clearly state at the beginning of the article |
| 2. | Are the main outcomes to be measured clearly described in the Introduction or Methods sections? | | Yes | | Described in the Methods section |
| 3. | Are the characteristics of the patients included in the study clearly described? | | Yes | | All relevant sociodemographic characteristics stated in Table 1 |
| 4. | Are the interventions of interest clearly described? | | Yes | | The program, including both treatment and control groups, is outlined clearly |
| 5. | Are the distributions of principal confounders in each group of subjects to be compared clearly described? | No | | | No mention of confounders, although many participant characteristics were controlled for in the regression |
| 6. | Are the main findings of the study clearly described? | | Yes | | |
| 7. | Does the study provide estimates of the random variability in the data for the main outcomes? | | Yes | | Standard deviation for all outcomes |

| | | | | | |
|---|---|---|---|---|---|
| 8. | Have all important adverse events that may be a consequence of the intervention been reported? | | Yes | | Unmet needs, physical and psychological risk |
| 9. | Have the characteristics of the patients lost to follow-up been described? | No | | | No follow up as this is a cross-sectional survey at a single time point |
| 10. | Have actual probability values been reported (e.g. 0.035 rather than <0.05) for the main outcomes except where the probability value is less than 0.001? | | Yes | | |
| | | | | | **Total reporting score: 8/11** |
| **External Validity** | | | | | |
| 11. | Were the subjects asked to participate in the study representative of the entire population from which they were recruited? | | Yes | | Sample was randomly drawn from a listing of all program recipients, stratified by service model, age and severity. |
| 12. | Were those subjects who were prepared to participate representative of the entire population from which they were recruited? | Unable to determine | | | Response rate stated (77.8%), but characteristics of non-responders not given |
| 13. | Were the staff, places, and facilities where the patients were treated, representative of the treatment the majority of patients receive? | | Yes | | |
| | | | | | **Total external validity score: 2/3** |
| **Internal Validity – Bias** | | | | | |
| 14. | Was an attempt made to blind study participants to the intervention they have received? | No | | | |
| 15. | Was an attempt made to blind those measuring outcomes of the intervention? | No | | | |
| 16. | If any of the results of the study were based on "data dredging", was this made clear? | | Yes | | No unplanned analysis reported |
| 17. | In trials and cohort studies, do the analyses adjust for different lengths of follow-up of patients, or in | Unable to determine | | | No information on how long participants have been receiving care |

| | | | | | |
|---|---|---|---|---|---|
| | case control studies, is the time period between the intervention and outcomes the same for cases and controls? | | | | |
| 18. | Were the statistical tests used to assess the main outcomes appropriate? | | Yes | | |
| 19. | Was compliance with the intervention/s reliable? | | Yes | | No reported non-compliance |
| 20. | Were the main outcome measures used accurate (valid and reliable)? | | Yes | | Refers to established measures from other published work |
| | | | | | **Total bias score: 4/7** |
| **Internal Validity – Confounding (Selection bias)** | | | | | |
| 21. | Were the patients in different intervention groups (trials and cohort studies) or were the cases and controls (case-control studies) recruited from the same population? | | Yes | | All recruited from the same program in California |
| 22. | Were study subjects in different intervention groups (trial and cohort studies) or were the cases and controls (case-control studies) recruited over the same period of time? | | Yes | | All interviews between October 1996 and March 1997 |
| 23. | Were study subjects randomised to intervention groups? | No | | | This is an observational study; participants were in their pre-existing groups |
| 24. | Was the randomised intervention assignment concealed from both patients and health care staff until recruitment was compete and irrevocable? | No | | | |
| 25. | Was there adequate adjustment for confounding in the analyses from which the main findings were drawn? | No | | | No adjustments for confounding in the main outcomes |
| 26. | Were losses of patients to follow-up taken into account? | Unable to determine | | | Not relevant for cross-sectional study, no follow up |
| | | | | | **Total confounding score: 2/6** |

| | | | | | |
|---|---|---|---|---|---|
| **Power** | | | | | |
| 27. | *Did the study conduct a power analysis to determine the sample size needed to detect a significant difference in effect size for one or more outcome measures?* | No | | | |
| | | | | | **Total power score: 0/1** |
| | | | | | |
| | | | | | **Total quality score: 16/28** |

---------------------------------------------------------------------------

**Author (year):** Brown (2007)
**Study Title:** Cash and Counseling: improving the lives of Medicaid beneficiaries who need personal care or home- and community-based services. Final report.
**Study Type:** Randomised controlled trial

| | | Score | | | |
|---|---|---|---|---|---|
| | | 0 | 1 | 2 | Comment |
| **Reporting** | | | | | |
| 1. | Is the hypothesis/aim/objective of the study clearly described? | | Yes | | Aims clearly outlined in the introduction: effect on consumers, caregivers (paid/unpaid) and costs. |
| 2. | Are the main outcomes to be measured clearly described in the Introduction or Methods sections? | | Yes | | Outcomes to be measured are listed in Section II: The Evaluation Design, Data and Methodology |
| 3. | Are the characteristics of the patients included in the study clearly described? | | Yes | | Characteristics for all sites and both groups (treatment and control) are provided in Appendix A |

| | | | | | |
|---|---|---|---|---|---|
| 4. | Are the interventions of interest clearly described? | | Yes | | Circumstances for both the treatment are control groups are described on pg. 9 |
| 5. | Are the distributions of principal confounders in each group of subjects to be compared clearly described? | No | | | No list of confounders is provided. There is a list of control variables used to control for differences between baseline characteristics. |
| 6. | Are the main findings of the study clearly described? | | Yes | | |
| 7. | Does the study provide estimates of the random variability in the data for the main outcomes? | No | | | No standard deviations, errors, or confidence intervals reported – all measures are based on binary outcomes |
| 8. | Have all important adverse events that may be a consequence of the intervention been reported? | | Yes | | Unmet needs, care-related health problems |
| 9. | Have the characteristics of the patients lost to follow-up been described? | | Yes | | Number of patients lost to follow up in each site/intervention-group and their age group reported. |
| 10. | Have actual probability values been reported (e.g. 0.035 rather than <0.05) for the main outcomes except where the probability value is less than 0.001? | | Yes | | |
| | | | | | **Total reporting score: 8/11** |
| **External Validity** | | | | | |
| 11. | Were the subjects asked to participate in the study representative of the entire population from which they were recruited? | | Yes | | All eligible beneficiaries were invited to participate |
| 12. | Were those subjects who were prepared to participate representative of the entire population from which they were recruited? | | Yes | | |

| | | | | | |
|---|---|---|---|---|---|
| 13. | Were the staff, places, and facilities where the patients were treated, representative of the treatment the majority of patients receive? | | Yes | | |
| | | | | | **Total external validity score: 3/3** |
| **Internal Validity – Bias** | | | | | |
| 14. | Was an attempt made to blind study participants to the intervention they have received? | No | | | |
| 15. | Was an attempt made to blind those measuring outcomes of the intervention? | No | | | |
| 16. | If any of the results of the study were based on “data dredging”, was this made clear? | | Yes | | |
| 17. | In trials and cohort studies, do the analyses adjust for different lengths of follow-up of patients, or in case control studies, is the time period between the intervention and outcomes the same for cases and controls? | | Yes | | Time period of enrolment controlled for in analysis |
| 18. | Were the statistical tests used to assess the main outcomes appropriate? | | Yes | | Multivariate logistic regression models |
| 19. | Was compliance with the intervention/s reliable? | | Yes | | No evidence of non-compliance |
| 20. | Were the main outcome measures used accurate (valid and reliable)? | | Yes | | No reliability factors reported, but how the outcomes are measured is clearly described |
| | | | | | **Total bias score: 5/7** |
| **Internal Validity – Confounding (Selection bias)** | | | | | |
| 21. | Were the patients in different intervention groups (trials and cohort studies) or were the cases and controls (case-control studies) recruited from the same population? | | Yes | | |
| 22. | Were study subjects in different intervention groups (trial and cohort studies) or were the cases and | | Yes | | Although this varied by site, but site results are reported separately |

| | | | | | |
|---|---|---|---|---|---|
| | controls (case-control studies) recruited over the same period of time? | | | | |
| 23. | Were study subjects randomised to intervention groups? | | Yes | | |
| 24. | Was the randomised intervention assignment concealed from both patients and health care staff until recruitment was compete and irrevocable? | | Yes | | Baseline interview was conducted before group assignment |
| 25. | Was there adequate adjustment for confounding in the analyses from which the main findings were drawn? | | Yes | | Baseline differences between groups controlled for in analysis |
| 26. | Were losses of patients to follow-up taken into account? | | Yes | | |
| | | | | | **Total confounding score: 6/6** |
| **Power** | | | | | |
| 27. | *Did the study conduct a power analysis to determine the sample size needed to detect a significant difference in effect size for one or more outcome measures?* | | Yes | | Reported for cost outcomes (pg. 19) and caregiver's outcomes (pg. 21). See Dale (2005) for statistical power by site and age group |
| | | | | | **Total power score: 1/1** |
| | | | | | |
| | | | | | **Total quality score: 23/28** |

---------------------------------------------------------------------------

---------------------------------------------------------------------------

**Author (year):** Caldwell (2007)
**Study Title:** Longitudinal outcomes of a consumer-directed program supporting adults with developmental disabilities and their families

| | | | | | |
|---|---|---|---|---|---|
| **Study Type:** Controlled before-after study | | | | | |
| | | Score | | | |
| | | 0 | 1 | 2 | Comment |
| **Reporting** | | | | | |
| 1. | Is the hypothesis/aim/objective of the study clearly described? | | Yes | | Study hypothesis clearly stated in the introduction |
| 2. | Are the main outcomes to be measured clearly described in the Introduction or Methods sections? | | Yes | | Details provided clearly in the Methods section |
| 3. | Are the characteristics of the patients included in the study clearly described? | | Yes | | Characteristics of treatment and control groups in Table 2 |
| 4. | Are the interventions of interest clearly described? | | Yes | | Services received by both treatment and control groups are clearly described in the Methods section |
| 5. | Are the distributions of principal confounders in each group of subjects to be compared clearly described? | | | Yes | Mean and SD of family caregiver's age given. |
| 6. | Are the main findings of the study clearly described? | | Yes | | In Tables and discussions in the text |
| 7. | Does the study provide estimates of the random variability in the data for the main outcomes? | | Yes | | Standard deviations provided |
| 8. | Have all important adverse events that may be a consequence of the intervention been reported? | | Yes | | Unmet needs, burden |
| 9. | Have the characteristics of the patients lost to follow-up been described? | | Yes | | Families lost due to attrition are documented in Table 1 |
| 10. | Have actual probability values been reported (e.g. 0.035 rather than <0.05) for the main outcomes except where the probability value is less than 0.001? | No | | | |
| | | | | | **Total reporting score: 10/11** |
| **External Validity** | | | | | |

| | | | | | |
|---|---|---|---|---|---|
| 11. | Were the subjects asked to participate in the study representative of the entire population from which they were recruited? | | Yes | | Random sample from waiting list |
| 12. | Were those subjects who were prepared to participate representative of the entire population from which they were recruited? | | Yes | | Details provided on % of respondents and the characteristics of both groups compared |
| 13. | Were the staff, places, and facilities where the patients were treated, representative of the treatment the majority of patients receive? | | Yes | | |
| | | | | | **Total external validity score: 3/3** |
| **Internal Validity – Bias** | | | | | |
| 14. | Was an attempt made to blind study participants to the intervention they have received? | No | | | |
| 15. | Was an attempt made to blind those measuring outcomes of the intervention? | No | | | |
| 16. | If any of the results of the study were based on "data dredging", was this made clear? | | Yes | | |
| 17. | In trials and cohort studies, do the analyses adjust for different lengths of follow-up of patients, or in case control studies, is the time period between the intervention and outcomes the same for cases and controls? | | Yes | | All families surveyed were in the program for 9 years. |
| 18. | Were the statistical tests used to assess the main outcomes appropriate? | | Yes | | Repeated measures analysis of variance and analysis of covariance |
| 19. | Was compliance with the intervention/s reliable? | Unable to determine | | | The control group may have been receiving significant services out-of-pocket. This is not considered in the analysis. |
| 20. | Were the main outcome measures used accurate (valid and reliable)? | | Yes | | Alpha reliability scores given for 3 out of 4 outcomes |

| | | | | | |
|---|---|---|---|---|---|
| **Total bias score: 4/7** | | | | | |
| **Internal Validity – Confounding (Selection bias)** | | | | | |
| 21. | Were the patients in different intervention groups (trials and cohort studies) or were the cases and controls (case-control studies) recruited from the same population? | | Yes | | All recruited from waiting list |
| 22. | Were study subjects in different intervention groups (trial and cohort studies) or were the cases and controls (case-control studies) recruited over the same period of time? | No | | | The control group was recruited at time 3 (9 years) the treatment groups at time 1. |
| 23. | Were study subjects randomised to intervention groups? | | Yes | | Treatment group selected by random lottery. Control group randomly selected from waiting list |
| 24. | Was the randomised intervention assignment concealed from both patients and health care staff until recruitment was complete and irrevocable? | No | | | |
| 25. | Was there adequate adjustment for confounding in the analyses from which the main findings were drawn? | | Yes | | Caregiver age was controlled for as this was found to be significantly different between groups |
| 26. | Were losses of patients to follow-up taken into account? | | Yes | | Only patients present for 9 years were surveyed |
| **Total confounding score: 4/6** | | | | | |
| **Power** | | | | | |
| 27. | *Did the study conduct a power analysis to determine the sample size needed to detect a significant difference in effect size for one or more outcome measures?* | No | | | |
| **Total power score: 0/1** | | | | | |
| | | | | | |
| **Total quality score: 21/28** | | | | | |

------------------------------------------------------------------------

| **Author (year):** Conroy (2002)<br>**Study Title:** Independent evaluation of California's self-determination pilot projects<br>**Study Type:** Controlled before-after study | | | | | |
|---|---|---|---|---|---|
| | | Score | | | |
| | | 0 | 1 | 2 | Comment |
| **Reporting** | | | | | |
| 1. | Is the hypothesis/aim/objective of the study clearly described? | | Yes | | The primary intent of the evaluation is outlined at the beginning |
| 2. | Are the main outcomes to be measured clearly described in the Introduction or Methods sections? | | Yes | | Outcomes, methodologies, and instruments described in the Methods section |
| 3. | Are the characteristics of the patients included in the study clearly described? | | Yes | | Participant characteristics detailed (age, gender, ethnic breakdown, level of mental retardation, living situation, ...) |
| 4. | Are the interventions of interest clearly described? | | Yes | | Both treatment and control group described |
| 5. | Are the distributions of principal confounders in each group of subjects to be compared clearly described? | No | | | No confounders listed |
| 6. | Are the main findings of the study clearly described? | | Yes | | |
| 7. | Does the study provide estimates of the random variability in the data for the main outcomes? | No | | | |
| 8. | Have all important adverse events that may be a consequence of the intervention been reported? | No | | | No adverse effects discussed |

| | | | | | |
|---|---|---|---|---|---|
| 9. | Have the characteristics of the patients lost to follow-up been described? | | Yes | | Characteristics of all participants provides, although those lost were not presented separately |
| 10. | Have actual probability values been reported (e.g. 0.035 rather than <0.05) for the main outcomes except where the probability value is less than 0.001? | No | | | |
| | | | | | **Total reporting score: 6/11** |
| **External Validity** | | | | | |
| 11. | Were the subjects asked to participate in the study representative of the entire population from which they were recruited? | | Yes | | Source population for each site identified. Participants selected randomly |
| 12. | Were those subjects who were prepared to participate representative of the entire population from which they were recruited? | Unable to determine | | | No information on response rates to request to participate |
| 13. | Were the staff, places, and facilities where the patients were treated, representative of the treatment the majority of patients receive? | | Yes | | |
| | | | | | **Total external validity score: 2/3** |
| **Internal Validity – Bias** | | | | | |
| 14. | Was an attempt made to blind study participants to the intervention they have received? | No | | | |
| 15. | Was an attempt made to blind those measuring outcomes of the intervention? | No | | | |
| 16. | If any of the results of the study were based on "data dredging", was this made clear? | | Yes | | Analysis of costs was not performed as anticipated at the outset, but this is clearly discussed in the evaluation |
| 17. | In trials and cohort studies, do the analyses adjust for different lengths of follow-up of patients, or in case control studies, is the time period between the | | Yes | | Analysis of costs had to adjust for this based on very different start dates |

| | | | | | |
|---|---|---|---|---|---|
| | intervention and outcomes the same for cases and controls? | | | | |
| 18. | Were the statistical tests used to assess the main outcomes appropriate? | Unable to determine | | | Very little information provided |
| 19. | Was compliance with the intervention/s reliable? | | Yes | | No non-compliance reported |
| 20. | Were the main outcome measures used accurate (valid and reliable)? | | Yes | | Reference to Personal Life Quality Protocol which has been "submitted to multiple tests of reliability" |
| | | | | | **Total bias score: 4/7** |
| **Internal Validity – Confounding (Selection bias)** | | | | | |
| 21. | Were the patients in different intervention groups (trials and cohort studies) or were the cases and controls (case-control studies) recruited from the same population? | No | | | 3 intervention groups from 3 different site, with differences in recruitment strategies. Control group only comparable with 1 site. |
| 22. | Were study subjects in different intervention groups (trial and cohort studies) or were the cases and controls (case-control studies) recruited over the same period of time? | No | | | Recruitment (start dates) were spread over a 2 year period |
| 23. | Were study subjects randomised to intervention groups? | | Yes | | It is started that subjects were randomly selected from the identified source populations |
| 24. | Was the randomised intervention assignment concealed from both patients and health care staff until recruitment was compete and irrevocable? | Unable to determine | | | No details on this provided |
| 25. | Was there adequate adjustment for confounding in the analyses from which the main findings were drawn? | No | | | No confounding factors were controlled for |
| 26. | Were losses of patients to follow-up taken into account? | | Yes | | Comparison only made for people with before and after observations |
| | | | | | **Total confounding score: 2/6** |

| | | | | | |
|---|---|---|---|---|---|
| **Power** | | | | | |
| 27. | *Did the study conduct a power analysis to determine the sample size needed to detect a significant difference in effect size for one or more outcome measures?* | No | | | |
| | | | | | **Total power score: 0/1** |
| | | | | | |
| | | | | | **Total quality score: 14/28** |

------------------------------------------------------------------------

**Author (year):** Cook (2008)
**Study Title:** A self-directed care model for mental health recovery
**Study Type:** Uncontrolled before-after study

| | | Score | | | |
|---|---|---|---|---|---|
| | | 0 | 1 | 2 | Comment |
| **Reporting** | | | | | |
| 1. | Is the hypothesis/aim/objective of the study clearly described? | No | | | There is an overview of the program followed by results, but no clear study objective identified |
| 2. | Are the main outcomes to be measured clearly described in the Introduction or Methods sections? | No | | | Main outcomes not described until the Program Evaluation and Outcomes section |
| 3. | Are the characteristics of the patients included in the study clearly described? | | Yes | | Reports age, gender, ethnicity, length of participation, civil status, living arrangements, education, disability |

| | | | | | |
|---|---|---|---|---|---|
| 4. | Are the interventions of interest clearly described? | | Yes | | The program is clearly described |
| 5. | Are the distributions of principal confounders in each group of subjects to be compared clearly described? | No | | | No confounders considered |
| 6. | Are the main findings of the study clearly described? | | Yes | | |
| 7. | Does the study provide estimates of the random variability in the data for the main outcomes? | | Yes | | Standard deviation reported |
| 8. | Have all important adverse events that may be a consequence of the intervention been reported? | No | | | No adverse effects reported |
| 9. | Have the characteristics of the patients lost to follow-up been described? | | Yes | | No losses to follow-up reported |
| 10. | Have actual probability values been reported (e.g. 0.035 rather than <0.05) for the main outcomes except where the probability value is less than 0.001? | No | | | |
| | | | | | **Total reporting score: 5/11** |
| **External Validity** | | | | | |
| 11. | Were the subjects asked to participate in the study representative of the entire population from which they were recruited? | Unable to determine | | | No information on how participants were chosen |
| 12. | Were those subjects who were prepared to participate representative of the entire population from which they were recruited? | Unable to determine | | | No information on how participants were recruited |

| | | | | | |
|---|---|---|---|---|---|
| 13. | Were the staff, places, and facilities where the patients were treated, representative of the treatment the majority of patients receive? | | Yes | | |
| | | | | | **Total external validity score: 1/3** |
| **Internal Validity – Bias** | | | | | |
| 14. | Was an attempt made to blind study participants to the intervention they have received? | No | | | |
| 15. | Was an attempt made to blind those measuring outcomes of the intervention? | No | | | |
| 16. | If any of the results of the study were based on "data dredging", was this made clear? | | Yes | | No unplanned analysis reported |
| 17. | In trials and cohort studies, do the analyses adjust for different lengths of follow-up of patients, or in case control studies, is the time period between the intervention and outcomes the same for cases and controls? | No | | | Length of time enrolled varied from 3 to 19 months, no adjusts made to reflect this |
| 18. | Were the statistical tests used to assess the main outcomes appropriate? | | Yes | | Paired t tests to compare outcomes before and after participation |
| 19. | Was compliance with the intervention/s reliable? | | Yes | | No non-compliance reported |
| 20. | Were the main outcome measures used accurate (valid and reliable)? | | Yes | | Global assessment of functioning scale cited |
| | | | | | **Total bias score: 4/7** |
| **Internal Validity – Confounding (Selection bias)** | | | | | |
| 21. | Were the patients in different intervention groups (trials and cohort studies) or were the cases and controls (case-control studies) recruited from the same population? | | Yes | | |

| | | | | | |
|---|---|---|---|---|---|
| 22. | Were study subjects in different intervention groups (trial and cohort studies) or were the cases and controls (case-control studies) recruited over the same period of time? | Unable to determine | | | Participants were followed from Nov 2002 to June 2004, but no information on when interviews/surveys were conducted and length of time enrolled varied from 3 to 19 months |
| 23. | Were study subjects randomised to intervention groups? | No | | | |
| 24. | Was the randomised intervention assignment concealed from both patients and health care staff until recruitment was compete and irrevocable? | No | | | |
| 25. | Was there adequate adjustment for confounding in the analyses from which the main findings were drawn? | No | | | No adjustments |
| 26. | Were losses of patients to follow-up taken into account? | | Yes | | No losses to follow up reported |
| | | | | | **Total confounding score: 2/6** |
| **Power** | | | | | |
| 27. | *Did the study conduct a power analysis to determine the sample size needed to detect a significant difference in effect size for one or more outcome measures?* | No | | | |
| | | | | | **Total power score: 0/1** |
| | | | | | |
| | | | | | **Total quality score: 12/28** |

--------------------------------------------------------------------------------

| **Author (year):** Cook (2019)<br>**Study Title:** Mental health self-directed care financing: efficacy in improving outcomes and controlling costs for adults with serious mental illness<br>**Study Type:** Randomised controlled trial | | | | | |
|---|---|---|---|---|---|
| | | Score | | | |
| | | 0 | 1 | 2 | Comment |
| **Reporting** | | | | | |
| 1. | Is the hypothesis/aim/objective of the study clearly described? | | Yes | | Stated at the outset |
| 2. | Are the main outcomes to be measured clearly described in the Introduction or Methods sections? | | Yes | | Detailed in Measures section |
| 3. | Are the characteristics of the patients included in the study clearly described? | | Yes | | Characteristics of treatment and control groups detailed in Table 1 |
| 4. | Are the interventions of interest clearly described? | | Yes | | |
| 5. | Are the distributions of principal confounders in each group of subjects to be compared clearly described? | | Partially | | No list of potential confounders provider, however participants were compared at baseline and statistical equivalence was shown. |
| 6. | Are the main findings of the study clearly described? | | Yes | | |
| 7. | Does the study provide estimates of the random variability in the data for the main outcomes? | | Yes | | Standard error reported |
| 8. | Have all important adverse events that may be a consequence of the intervention been reported? | No | | | No adverse effects reported |
| 9. | Have the characteristics of the patients lost to follow-up been described? | | Yes | | Characteristics of all baseline sample provided, losses to mortality reported |

| | | | | | |
|---|---|---|---|---|---|
| 10. | Have actual probability values been reported (e.g. 0.035 rather than <0.05) for the main outcomes except where the probability value is less than 0.001? | | Yes | | |
| | | | | | **Total reporting score: 9/11** |
| **External Validity** | | | | | |
| 11. | Were the subjects asked to participate in the study representative of the entire population from which they were recruited? | | Yes | | Potential participants were all recruited at community mental health agencies |
| 12. | Were those subjects who were prepared to participate representative of the entire population from which they were recruited? | Unable to determine | | | The portion asked and who agreed is not reported |
| 13. | Were the staff, places, and facilities where the patients were treated, representative of the treatment the majority of patients receive? | | Yes | | |
| | | | | | **Total external validity score: 2/3** |
| **Internal Validity – Bias** | | | | | |
| 14. | Was an attempt made to blind study participants to the intervention they have received? | No | | | |
| 15. | Was an attempt made to blind those measuring outcomes of the intervention? | No | | | |
| 16. | If any of the results of the study were based on "data dredging", was this made clear? | | Yes | | No unplanned analysis reported |
| 17. | In trials and cohort studies, do the analyses adjust for different lengths of follow-up of patients, or in case control studies, is the time period between the intervention and outcomes the same for cases and controls? | | Yes | | Follow up was the same for all participants |
| 18. | Were the statistical tests used to assess the main outcomes appropriate? | | Yes | | |

| | | | | | |
|---|---|---|---|---|---|
| 19. | Was compliance with the intervention/s reliable? | | Yes | | No reported non-compliance, fidelity checks were conducted |
| 20. | Were the main outcome measures used accurate (valid and reliable)? | | Yes | | Cronbach's alpha reported for all outcome measures |
| | | | | | **Total bias score: 5/7** |
| **Internal Validity – Confounding (Selection bias)** | | | | | |
| 21. | Were the patients in different intervention groups (trials and cohort studies) or were the cases and controls (case-control studies) recruited from the same population? | | Yes | | |
| 22. | Were study subjects in different intervention groups (trial and cohort studies) or were the cases and controls (case-control studies) recruited over the same period of time? | | Yes | | |
| 23. | Were study subjects randomised to intervention groups? | | Yes | | Randomly generated allocation sequence used |
| 24. | Was the randomised intervention assignment concealed from both patients and health care staff until recruitment was compete and irrevocable? | | Yes | | Complete allocation concealment until after assignment occurred |
| 25. | Was there adequate adjustment for confounding in the analyses from which the main findings were drawn? | | Yes | | |
| 26. | Were losses of patients to follow-up taken into account? | | Yes | | |
| | | | | | **Total confounding score: 6/6** |
| **Power** | | | | | |
| 27. | *Did the study conduct a power analysis to determine the sample size needed to detect a significant difference in effect size for one or more outcome measures?* | No | | | |

| |
|---|
| **Total power score: 0/1** |
| |
| **Total quality score: 22/28** |

------------------------------------------------------------------------

| **Author (year):** Croft (2019)<br>**Study Title:** Service costs and mental health self-direction: findings from consumer recovery investment fund self-directed care<br>**Study Type:** Controlled before-after study | | | | | |
|---|---|---|---|---|---|
| | | Score | | | |
| | | 0 | 1 | 2 | Comment |
| **Reporting** | | | | | |
| 1. | Is the hypothesis/aim/objective of the study clearly described? | | Yes | | Stated at outset |
| 2. | Are the main outcomes to be measured clearly described in the Introduction or Methods sections? | | Yes | | In Methods section |
| 3. | Are the characteristics of the patients included in the study clearly described? | | Yes | | Gender, age, and ethnicity reported |
| 4. | Are the interventions of interest clearly described? | | Yes | | Treatment program described |
| 5. | Are the distributions of principal confounders in each group of subjects to be compared clearly described? | No | | | No list of confounders reported. |
| 6. | Are the main findings of the study clearly described? | | Yes | | |
| 7. | Does the study provide estimates of the random variability in the data for the main outcomes? | | Yes | | Standard deviation reported |
| 8. | Have all important adverse events that may be a consequence of the intervention been reported? | No | | | No adverse effects reported |

| | | | | | |
|---|---|---|---|---|---|
| 9. | Have the characteristics of the patients lost to follow-up been described? | | Yes | | They are included in the reported sample as they participated for at least 1 year |
| 10. | Have actual probability values been reported (e.g. 0.035 rather than <0.05) for the main outcomes except where the probability value is less than 0.001? | No | | | |
| | | | | | **Total reporting score: 7/11** |
| **External Validity** | | | | | |
| 11. | Were the subjects asked to participate in the study representative of the entire population from which they were recruited? | | Yes | | Participants are from a previous trial that was an RCT |
| 12. | Were those subjects who were prepared to participate representative of the entire population from which they were recruited? | | Yes | | Participants from a previous study asked to take part and portion agreeing is reported |
| 13. | Were the staff, places, and facilities where the patients were treated, representative of the treatment the majority of patients receive? | | Yes | | |
| | | | | | **Total external validity score: 3/3** |
| **Internal Validity – Bias** | | | | | |
| 14. | Was an attempt made to blind study participants to the intervention they have received? | No | | | |
| 15. | Was an attempt made to blind those measuring outcomes of the intervention? | No | | | |
| 16. | If any of the results of the study were based on “data dredging”, was this made clear? | | Yes | | No unplanned analysis reported |
| 17. | In trials and cohort studies, do the analyses adjust for different lengths of follow-up of patients, or in case control studies, is the time period between the | | Yes | | Results were adjusted for the number of months for which data was available |

| | | | | | |
|---|---|---|---|---|---|
| | intervention and outcomes the case for cases and controls? | | | | |
| 18. | Were the statistical tests used to assess the main outcomes appropriate? | | Yes | | |
| 19. | Was compliance with the intervention/s reliable? | | Yes | | No reported non-compliance |
| 20. | Were the main outcome measures used accurate (valid and reliable)? | | Yes | | |
| | | | | | **Total bias score: 5/7** |
| **Internal Validity – Confounding (Selection bias)** | | | | | |
| 21. | Were the patients in different intervention groups (trials and cohort studies) or were the cases and controls (case-control studies) recruited from the same population? | | Yes | | |
| 22. | Were study subjects in different intervention groups (trial and cohort studies) or were the cases and controls (case-control studies) recruited over the same period of time? | | Yes | | No stated differently |
| 23. | Were study subjects randomised to intervention groups? | No | | | |
| 24. | Was the randomised intervention assignment concealed from both patients and health care staff until recruitment was compete and irrevocable? | No | | | |
| 25. | Was there adequate adjustment for confounding in the analyses from which the main findings were drawn? | No | | | |
| 26. | Were losses of patients to follow-up taken into account? | | Yes | | Results were adjusted to reflect less months of participation by people who left the program |
| | | | | | **Total confounding score: 3/6** |
| **Power** | | | | | |

| | | | | | |
|---|---|---|---|---|---|
| 27. | *Did the study conduct a power analysis to determine the sample size needed to detect a significant difference in effect size for one or more outcome measures?* | No | | | |
| | | | | | **Total power score: 0/1** |
| | | | | | |
| | | | | | **Total quality score: 18/28** |

-----------------------------------------------------------------------

| **Author (year):** Croft (2020)<br>**Study Title:** Service Utilization before and after self-direction: a quasi-experimental difference-in-difference analysis of Utah's Mental Health Access to Recovery program.<br>**Study Type:** Controlled before-after study | | | | | |
|---|---|---|---|---|---|
| | | Score | | | |
| | | 0 | 1 | 2 | Comment |
| **Reporting** | | | | | |
| 1. | Is the hypothesis/aim/objective of the study clearly described? | | Yes | | Clearly stated objective of examining service utilization in four categories |
| 2. | Are the main outcomes to be measured clearly described in the Introduction or Methods sections? | | Yes | | Detailed in methods section |
| 3. | Are the characteristics of the patients included in the study clearly described? | | Yes | | Characteristics before and after matching given, demographics, functioning, service use, etc provided at first observation |
| 4. | Are the interventions of interest clearly described? | | Yes | | Clear description of program and comparison given |
| 5. | Are the distributions of principal confounders in each group of subjects to be compared clearly described? | No | | | No specific mention of confounding factors. But mean and standard |

| | | | | | |
|---|---|---|---|---|---|
| | | | | | deviations of all relevant covariates provided. |
| 6. | Are the main findings of the study clearly described? | | Yes | | Clearly described in the Results section and in Table |
| 7. | Does the study provide estimates of the random variability in the data for the main outcomes? | | Yes | | Standard errors reported |
| 8. | Have all important adverse events that may be a consequence of the intervention been reported? | No | | | No adverse effects reported (report uses administrative, not personal, data |
| 9. | Have the characteristics of the patients lost to follow-up been described? | No | | | Participants dropped from study due to lack of follow-up data, their characteristics are not described |
| 10. | Have actual probability values been reported (e.g. 0.035 rather than <0.05) for the main outcomes except where the probability value is less than 0.001? | | Yes | | |
| | | | | | **Total reporting score: 7/11** |
| **External Validity** | | | | | |
| 11. | Were the subjects asked to participate in the study representative of the entire population from which they were recruited? | Unable to determine | | | Participation in the MHATR program was voluntary |
| 12. | Were those subjects who were prepared to participate representative of the entire population from which they were recruited? | Unable to determine | | | |
| 13. | Were the staff, places, and facilities where the patients were treated, representative of the treatment the majority of patients receive? | | Yes | | Services purchases through budget were representative of what was available to the general population. |
| | | | | | **Total external validity score: 1/3** |
| **Internal Validity – Bias** | | | | | |
| 14. | Was an attempt made to blind study participants to the intervention they have received? | No | | | |

| | | | | | |
|---|---|---|---|---|---|
| 15. | Was an attempt made to blind those measuring outcomes of the intervention? | No | | | |
| 16. | If any of the results of the study were based on “data dredging”, was this made clear? | | Yes | | |
| 17. | In trials and cohort studies, do the analyses adjust for different lengths of follow-up of patients, or in case control studies, is the time period between the intervention and outcomes the same for cases and controls? | | Yes | | Baseline and follow-up observations were assigned based on enrolment year. Sensitivity analysis was conducted to see effect of shorter observation periods. |
| 18. | Were the statistical tests used to assess the main outcomes appropriate? | | Yes | | |
| 19. | Was compliance with the intervention/s reliable? | | Yes | | No reported non-compliance |
| 20. | Were the main outcome measures used accurate (valid and reliable)? | | Yes | | Measurement methodologies clearly described |
| | | | | | **Total bias score: 5/7** |
| **Internal Validity – Confounding (Selection bias)** | | | | | |
| 21. | Were the patients in different intervention groups (trials and cohort studies) or were the cases and controls (case-control studies) recruited from the same population? | | Yes | | Both MHATR and comparison group individuals were adult public health service users in Utah, with the same eligibility criteria |
| 22. | Were study subjects in different intervention groups (trial and cohort studies) or were the cases and controls (case-control studies) recruited over the same period of time? | Unable to determine | | | People were enrolled at various times from 2013 to 2015, for an average of 199 days. |
| 23. | Were study subjects randomised to intervention groups? | No | | | |
| 24. | Was the randomised intervention assignment concealed from both patients and health care staff until recruitment was compete and irrevocable? | No | | | |

| | | | | | |
|---|---|---|---|---|---|
| 25. | Was there adequate adjustment for confounding in the analyses from which the main findings were drawn? | | Yes | | Relevant covariates were controlled for in regression analysis |
| 26. | Were losses of patients to follow-up taken into account? | | Yes | | Only a small percentage did not have 2 data observations |
| | | | | | **Total confounding score: 3/6** |
| **Power** | | | | | |
| 27. | *Did the study conduct a power analysis to determine the sample size needed to detect a significant difference in effect size for one or more outcome measures?*<br>*Note: question modified* | No | | | |
| | | | | | **Total power score: 0/1** |
| | | | | | |
| | | | | | **Total quality score: 16/28** |

---------------------------------------------------------------------------

**Author (year):** Fisher (2008)
**Study Title:** Attendant care program. Direct funding pilot evaluation. Final report
**Study Type:** Controlled before-after study

| | | Score | | | |
|---|---|---|---|---|---|
| | | 0 | 1 | 2 | Comment |
| **Reporting** | | | | | |
| 1. | Is the hypothesis/aim/objective of the study clearly described? | | Yes | | The intention of the study is described |

| | | | | | |
|---|---|---|---|---|---|
| 2. | Are the main outcomes to be measured clearly described in the Introduction or Methods sections? | No | | | Outcomes to be measured and their results are presented together |
| 3. | Are the characteristics of the patients included in the study clearly described? | | Yes | | Reports age, gender, diversity, type of disability, location, support, and employment |
| 4. | Are the interventions of interest clearly described? | | Yes | | Treatment and control groups described |
| 5. | Are the distributions of principal confounders in each group of subjects to be compared clearly described? | No | | | |
| 6. | Are the main findings of the study clearly described? | | Yes | | |
| 7. | Does the study provide estimates of the random variability in the data for the main outcomes? | No | | | |
| 8. | Have all important adverse events that may be a consequence of the intervention been reported? | No | | | No adverse health effects reported, only time responsibility considered |
| 9. | Have the characteristics of the patients lost to follow-up been described? | | Yes | | No losses to follow up reported |
| 10. | Have actual probability values been reported (e.g. 0.035 rather than <0.05) for the main outcomes except where the probability value is less than 0.001? | No | | | |
| | | | | | **Total reporting score: 5/11** |
| **External Validity** | | | | | |

| | | | | | |
|---|---|---|---|---|---|
| 11. | Were the subjects asked to participate in the study representative of the entire population from which they were recruited? | Unable to determine | | | No information provided on how people were selected |
| 12. | Were those subjects who were prepared to participate representative of the entire population from which they were recruited? | Unable to determine | | | No information provided on how people were selected |
| 13. | Were the staff, places, and facilities where the patients were treated, representative of the treatment the majority of patients receive? | | Yes | | |
| | | | | | **Total external validity score: 1/3** |
| **Internal Validity – Bias** | | | | | |
| 14. | Was an attempt made to blind study participants to the intervention they have received? | No | | | |
| 15. | Was an attempt made to blind those measuring outcomes of the intervention? | No | | | |
| 16. | If any of the results of the study were based on "data dredging", was this made clear? | | Yes | | No unplanned analysis reported |
| 17. | In trials and cohort studies, do the analyses adjust for different lengths of follow-up of patients, or in case control studies, is the time period between the intervention and outcomes the same for cases and controls? | | Yes | | Time period is the same |
| 18. | Were the statistical tests used to assess the main outcomes appropriate? | | Yes | | Chi squared test |
| 19. | Was compliance with the intervention/s reliable? | | Yes | | No reported non-compliance |
| 20. | Were the main outcome measures used accurate (valid and reliable)? | | Yes | | Personal Wellbeing Index- stated it is internationally validated |

| | | | | | |
|---|---|---|---|---|---|
| | | | | | **Total bias score: 5/7** |
| **Internal Validity – Confounding (Selection bias)** | | | | | |
| 21. | Were the patients in different intervention groups (trials and cohort studies) or were the cases and controls (case-control studies) recruited from the same population? | Unable to determine | | | Very little information on recruitment provided |
| 22. | Were study subjects in different intervention groups (trial and cohort studies) or were the cases and controls (case-control studies) recruited over the same period of time? | Unable to determine | | | |
| 23. | Were study subjects randomised to intervention groups? | No | | | |
| 24. | Was the randomised intervention assignment concealed from both patients and health care staff until recruitment was compete and irrevocable? | No | | | |
| 25. | Was there adequate adjustment for confounding in the analyses from which the main findings were drawn? | No | | | |
| 26. | Were losses of patients to follow-up taken into account? | | Yes | | No losses to follow up reported |
| | | | | | **Total confounding score: 1/6** |
| **Power** | | | | | |
| 27. | *Did the study conduct a power analysis to determine the sample size needed to detect a significant difference in effect size for one or more outcome measures?* | No | | | |

| |
|---:|
| **Total power score: 0/1** |
| |
| **Total quality score: 12/28** |

------------------------------------------------------------------------

| **Author (year):** Fontecedro (2020)<br>**Study Title:** Individual health budgets in mental health: results of its implementation in the Friuli Venezia Giulia Region, Ital.<br>**Study Type:** Controlled cross-sectional study | | | | | |
|---|---|---|---|---|---|
| | | Score | | | |
| | | 0 | 1 | 2 | Comment |
| **Reporting** | | | | | |
| 1. | Is the hypothesis/aim/objective of the study clearly described? | | Yes | | The aim is clearly outlined at the end of the introduction |
| 2. | Are the main outcomes to be measured clearly described in the Introduction or Methods sections? | | Yes | | Described in Methods section |
| 3. | Are the characteristics of the patients included in the study clearly described? | | Yes | | All sociodemographic and clinical characteristics provided for both treatment and comparison groups |
| 4. | Are the interventions of interest clearly described? | | Yes | | IHB described, comparison group services as maintained "as usual" |
| 5. | Are the distributions of principal confounders in each group of subjects to be compared clearly described? | No | | | No explicit list of confounders given |
| 6. | Are the main findings of the study clearly described? | | Yes | | Results provided in tables and discussion in the text |

| | | | | | |
|---|---|---|---|---|---|
| 7. | Does the study provide estimates of the random variability in the data for the main outcomes? | | Yes | | Standard deviation reported |
| 8. | Have all important adverse events that may be a consequence of the intervention been reported? | | Yes | | HoONS scales measures a variety of problems |
| 9. | Have the characteristics of the patients lost to follow-up been described? | No | | | No follow-up, this is a cross-sectional design |
| 10. | Have actual probability values been reported (e.g. 0.035 rather than <0.05) for the main outcomes except where the probability value is less than 0.001? | No | | | No $p>0.01$ reported |
| | | | | | **Total reporting score: 7/11** |
| **External Validity** | | | | | |
| 11. | Were the subjects asked to participate in the study representative of the entire population from which they were recruited? | | Yes | | Participants, both treatment and control, were randomly selected from all patients in the 4 community health centres in the region of interest |
| 12. | Were those subjects who were prepared to participate representative of the entire population from which they were recruited? | No | | | Number of non respondents reported, but no validation that the final sample was representative of the source population |
| 13. | Were the staff, places, and facilities where the patients were treated, representative of the treatment the majority of patients receive? | | Yes | | |
| | | | | | **Total external validity score: 2/3** |
| **Internal Validity – Bias** | | | | | |
| 14. | Was an attempt made to blind study participants to the intervention they have received? | No | | | |
| 15. | Was an attempt made to blind those measuring outcomes of the intervention? | No | | | |

| | | | | | |
|---|---|---|---|---|---|
| 16. | If any of the results of the study were based on "data dredging", was this made clear? | | Yes | | No unforeseen analysis reported |
| 17. | In trials and cohort studies, do the analyses adjust for different lengths of follow-up of patients, or in case control studies, is the time period between the intervention and outcomes the same for cases and controls? | Unable to determine | | | It is not reported how long people have been receiving IHB, only that they were in receipt of one on 31 December 2019. |
| 18. | Were the statistical tests used to assess the main outcomes appropriate? | | Yes | | |
| 19. | Was compliance with the intervention/s reliable? | | Yes | | No non-compliance reported |
| 20. | Were the main outcome measures used accurate (valid and reliable)? | | Yes | | HoNOS scale is an established scale |
| | | | | | **Total bias score: 4/7** |
| **Internal Validity – Confounding (Selection bias)** | | | | | |
| 21. | Were the patients in different intervention groups (trials and cohort studies) or were the cases and controls (case-control studies) recruited from the same population? | | Yes | | All recruited from the 4 community health centres in Trieste |
| 22. | Were study subjects in different intervention groups (trial and cohort studies) or were the cases and controls (case-control studies) recruited over the same period of time? | | Yes | | |
| 23. | Were study subjects randomised to intervention groups? | No | | | The researchers did not assign people to groups, people were already in receipt of the treatment. |
| 24. | Was the randomised intervention assignment concealed from both patients and health care staff until recruitment was compete and irrevocable? | No | | | Not relevant |

| | | | | | |
|---|---|---|---|---|---|
| 25. | Was there adequate adjustment for confounding in the analyses from which the main findings were drawn? | No | | | IN the outcome of interest, nothing was controlled for |
| 26. | Were losses of patients to follow-up taken into account? | No | | | Not applicable for cross-sectional study |
| | | | | | **Total confounding score: 2/6** |
| **Power** | | | | | |
| 27. | *Did the study conduct a power analysis to determine the sample size needed to detect a significant difference in effect size for one or more outcome measures?* | No | | | |
| | | | | | **Total power score: 0/1** |
| | | | | | |
| | | | | | **Total quality score: 15/28** |

-----------------------------------------------------------------------

**Author (year):** Forder (2012)
**Study Title:** Evaluation of the personal health budget pilot programme
**Study Type:** Non-randomised controlled trial

| | | Score | | | |
|---|---|---|---|---|---|
| | | 0 | 1 | 2 | Comment |
| **Reporting** | | | | | |
| 1. | Is the hypothesis/aim/objective of the study clearly described? | | Yes | | Aim of the evaluation outlined in the Introduction |
| 2. | Are the main outcomes to be measured clearly described in the Introduction or Methods sections? | | Yes | | Described in Methods section |

| | | | | | |
|---|---|---|---|---|---|
| 3. | Are the characteristics of the patients included in the study clearly described? | | Yes | | Reports age, gender, ethnicity, sexual orientation, living arrangements, level of needs, etc. |
| 4. | Are the interventions of interest clearly described? | | Yes | | |
| 5. | Are the distributions of principal confounders in each group of subjects to be compared clearly described? | | Partially | | Confounding variables discuss but distributions not stated (only means) |
| 6. | Are the main findings of the study clearly described? | | Yes | | |
| 7. | Does the study provide estimates of the random variability in the data for the main outcomes? | | Yes | | Standard deviation reported |
| 8. | Have all important adverse events that may be a consequence of the intervention been reported? | | Yes | | Adverse health effects included in measures |
| 9. | Have the characteristics of the patients lost to follow-up been described? | No | | | The number of patients lost to follow-up is reported but their characteristics are not reported |
| 10. | Have actual probability values been reported (e.g. 0.035 rather than <0.05) for the main outcomes except where the probability value is less than 0.001? | | Yes | | |
| | | | | | **Total reporting score: 9/11** |
| **External Validity** | | | | | |
| 11. | Were the subjects asked to participate in the study representative of the entire population from which they were recruited? | Unable to determine | | | The selection procedure at each site is not clearly documented. It is stated that pilot sites were selecting or excluding specific groups of patients. |
| 12. | Were those subjects who were prepared to participate representative of the entire population from which they were recruited? | Unable to determine | | | The proportion of those asked who agreed to participate is not stated |

| | | | | | |
|---|---|---|---|---|---|
| 13. | Were the staff, places, and facilities where the patients were treated, representative of the treatment the majority of patients receive? | | Yes | | |
| **Total external validity score: 1/3** | | | | | |
| **Internal Validity – Bias** | | | | | |
| 14. | Was an attempt made to blind study participants to the intervention they have received? | No | | | |
| 15. | Was an attempt made to blind those measuring outcomes of the intervention? | No | | | |
| 16. | If any of the results of the study were based on “data dredging”, was this made clear? | | Yes | | Subgroup analysis is discussed at the outset |
| 17. | In trials and cohort studies, do the analyses adjust for different lengths of follow-up of patients, or in case control studies, is the time period between the intervention and outcomes the case for cases and controls? | | Yes | | Interviews and questionnaire were carried out at fixed intervals after enrolment |
| 18. | Were the statistical tests used to assess the main outcomes appropriate? | | Yes | | Difference-in-difference method |
| 19. | Was compliance with the intervention/s reliable? | | Yes | | |
| 20. | Were the main outcome measures used accurate (valid and reliable)? | | Yes | | ASCOT, GHQ |
| **Total bias score: 5/7** | | | | | |
| **Internal Validity – Confounding (Selection bias)** | | | | | |
| 21. | Were the patients in different intervention groups (trials and cohort studies) or were the cases and controls (case-control studies) recruited from the same population? | | Yes | | |
| 22. | Were study subjects in different intervention groups (trial and cohort studies) or were the cases and | | Yes | | |

| | | | | | |
|---|---|---|---|---|---|
| | controls (case-control studies) recruited over the same period of time? | | | | |
| 23. | Were study subjects randomised to intervention groups? | No | | | Randomisation took place on some sites and not on others |
| 24. | Was the randomised intervention assignment concealed from both patients and health care staff until recruitment was compete and irrevocable? | No | | | |
| 25. | Was there adequate adjustment for confounding in the analyses from which the main findings were drawn? | | Yes | | |
| 26. | Were losses of patients to follow-up taken into account? | | Yes | | Missing data was imputed |
| | | | | | **Total confounding score: 4/6** |
| **Power** | | | | | |
| 27. | *Did the study conduct a power analysis to determine the sample size needed to detect a significant difference in effect size for one or more outcome measures?* | | Yes | | |
| | | | | | **Total power score: 1/1** |
| | | | | | |
| | | | | | **Total quality score: 20/28** |

---------------------------------------------------------------------------

| **Author (year):** Glendinning (2008)<br>**Study Title:** Evaluation of the individual budgets pilot programme. Final report.<br>**Study Type:** randomised controlled trial | | | | | |
|---|---|---|---|---|---|
| | | Score | | | |
| | | 0 | 1 | 2 | Comment |

| | | | | | |
|---|---|---|---|---|---|
| **Reporting** | | | | | |
| 1. | Is the hypothesis/aim/objective of the study clearly described? | | Yes | | Aim of the evaluation clearly stated |
| 2. | Are the main outcomes to be measured clearly described in the Introduction or Methods sections? | | Yes | | Described in the Methods section of the Outcomes chapter |
| 3. | Are the characteristics of the patients included in the study clearly described? | | Yes | | Reports age, gender, ethnicity, type of disability, living conditions, etc |
| 4. | Are the interventions of interest clearly described? | | Yes | | |
| 5. | Are the distributions of principal confounders in each group of subjects to be compared clearly described? | | Partially | | It is stated that confounders in Table 7.6 are accounted for. So, I presume this is the list. But no distributions are given (means given in sample description) |
| 6. | Are the main findings of the study clearly described? | | Yes | | |
| 7. | Does the study provide estimates of the random variability in the data for the main outcomes? | | Yes | | Standard deviations reported |
| 8. | Have all important adverse events that may be a consequence of the intervention been reported? | | Yes | | Risk of psychological ill-health |
| 9. | Have the characteristics of the patients lost to follow-up been described? | No | | | Numbers are reported but not their characteristics |
| 10. | Have actual probability values been reported (e.g. 0.035 rather than <0.05) for the main outcomes except where the probability value is less than 0.001? | No | | | Actual value reported sometime in a footnote or in the text, but generally not reported |
| | | | | | **Total reporting score: 8/11** |
| **External Validity** | | | | | |
| 11. | Were the subjects asked to participate in the study representative of the entire population from which they were recruited? | Unable to determine | | | Different sites targeted different people, so it is not clear how representative it was of the wider social care population |

| | | | | | |
|---|---|---|---|---|---|
| 12. | Were those subjects who were prepared to participate representative of the entire population from which they were recruited? | Unable to determine | | | |
| 13. | Were the staff, places, and facilities where the patients were treated, representative of the treatment the majority of patients receive? | | Yes | | |
| | | | | | **Total external validity score: 1/3** |
| **Internal Validity – Bias** | | | | | |
| 14. | Was an attempt made to blind study participants to the intervention they have received? | No | | | |
| 15. | Was an attempt made to blind those measuring outcomes of the intervention? | No | | | |
| 16. | If any of the results of the study were based on "data dredging", was this made clear? | | Yes | | |
| 17. | In trials and cohort studies, do the analyses adjust for different lengths of follow-up of patients, or in case control studies, is the time period between the intervention and outcomes the case for cases and controls? | | Yes | | 6 month time period |
| 18. | Were the statistical tests used to assess the main outcomes appropriate? | | Yes | | |
| 19. | Was compliance with the intervention/s reliable? | | Yes | | Some people in the comparison group tried to pressure their care providers for an IB, but no specific non-compliance is reported |
| 20. | Were the main outcome measures used accurate (valid and reliable)? | | Yes | | Ascot, GHQ |
| | | | | | **Total bias score: 5/7** |
| **Internal Validity – Confounding (Selection bias)** | | | | | |

| | | | | | |
|---|---|---|---|---|---|
| 21. | Were the patients in different intervention groups (trials and cohort studies) or were the cases and controls (case-control studies) recruited from the same population? | | Yes | | |
| 22. | Were study subjects in different intervention groups (trial and cohort studies) or were the cases and controls (case-control studies) recruited over the same period of time? | | Yes | | |
| 23. | Were study subjects randomised to intervention groups? | | Yes | | Randomisation using a web-based tool |
| 24. | Was the randomised intervention assignment concealed from both patients and health care staff until recruitment was compete and irrevocable? | | Yes | | IB was offered after baseline data had been collected |
| 25. | Was there adequate adjustment for confounding in the analyses from which the main findings were drawn? | | Yes | | Various factors controlled for in analysis, e.g. previous direct payment, proxy responder, baseline characteristics |
| 26. | Were losses of patients to follow-up taken into account? | | Yes | | Numbers lost to follow-up are clearly details. Only people with baseline and follow-up data were included in the final evaluation sample |
| | | | | | **Total confounding score: 6/6** |
| **Power** | | | | | |
| 27. | *Did the study conduct a power analysis to determine the sample size needed to detect a significant difference in effect size for one or more outcome measures?* | | Yes | | |
| | | | | | **Total power score: 1/1** |
| | | | | | |
| | | | | | **Total quality score: 21/28** |

-----------------------------------------------------------------------

**Author (year):** Hagglund (2004)
**Study Title:** A comparison of consumer-directed ad agency-directed personal assistance services programmes
**Study Type:** Controlled cross-sectional study

| | | Score | | | |
|---|---|---|---|---|---|
| | | 0 | 1 | 2 | Comment |
| **Reporting** | | | | | |
| 1. | Is the hypothesis/aim/objective of the study clearly described? | | Yes | | Detailed in the Objectives of the Research section |
| 2. | Are the main outcomes to be measured clearly described in the Introduction or Methods sections? | | Yes | | Describe in Methods section |
| 3. | Are the characteristics of the patients included in the study clearly described? | | Yes | | Reports on age, gender, race, education, and employment |
| 4. | Are the interventions of interest clearly described? | | Yes | | |
| 5. | Are the distributions of principal confounders in each group of subjects to be compared clearly described? | No | | | No list of confounders given |
| 6. | Are the main findings of the study clearly described? | | Yes | | |
| 7. | Does the study provide estimates of the random variability in the data for the main outcomes? | | Yes | | Standard deviation reported |
| 8. | Have all important adverse events that may be a consequence of the intervention been reported? | | Yes | | Unmet needs reported |
| 9. | Have the characteristics of the patients lost to follow-up been described? | No | | | No follow-up as it is a cross-sectional survey |
| 10. | Have actual probability values been reported (e.g. 0.035 rather than <0.05) for the main outcomes | No | | | Values only reported for p>0.01 |

| | | | | | |
|---|---|---|---|---|---|
| | except where the probability value is less than 0.001? | | | | |
| **Total reporting score: 7/11** | | | | | |
| **External Validity** | | | | | |
| 11. | Were the subjects asked to participate in the study representative of the entire population from which they were recruited? | | Yes | | Source population described |
| 12. | Were those subjects who were prepared to participate representative of the entire population from which they were recruited? | | Yes | | Number responding reports, and reason for not responding is explained |
| 13. | Were the staff, places, and facilities where the patients were treated, representative of the treatment the majority of patients receive? | | Yes | | |
| **Total external validity score: 3/3** | | | | | |
| **Internal Validity – Bias** | | | | | |
| 14. | Was an attempt made to blind study participants to the intervention they have received? | No | | | |
| 15. | Was an attempt made to blind those measuring outcomes of the intervention? | No | | | |
| 16. | If any of the results of the study were based on “data dredging”, was this made clear? | | Yes | | |
| 17. | In trials and cohort studies, do the analyses adjust for different lengths of follow-up of patients, or in case control studies, is the time period between the intervention and outcomes the case for cases and controls? | No | | | Some people enrolment for only 1 month, and average of 5 years |
| 18. | Were the statistical tests used to assess the main outcomes appropriate? | | Yes | | |
| 19. | Was compliance with the intervention/s reliable? | | Yes | | |

| | | | | | |
|---|---|---|---|---|---|
| 20. | Were the main outcome measures used accurate (valid and reliable)? | | Yes | | Measurement scales cited from other sources |
| | | | | | **Total bias score: 4/7** |
| **Internal Validity – Confounding (Selection bias)** | | | | | |
| 21. | Were the patients in different intervention groups (trials and cohort studies) or were the cases and controls (case-control studies) recruited from the same population? | | Yes | | All from Missouri |
| 22. | Were study subjects in different intervention groups (trial and cohort studies) or were the cases and controls (case-control studies) recruited over the same period of time? | | Yes | | |
| 23. | Were study subjects randomised to intervention groups? | No | | | |
| 24. | Was the randomised intervention assignment concealed from both patients and health care staff until recruitment was compete and irrevocable? | No | | | |
| 25. | Was there adequate adjustment for confounding in the analyses from which the main findings were drawn? | | Yes | | Regression analysis controlled for various sociodemographic characteristics |
| 26. | Were losses of patients to follow-up taken into account? | No | | | |
| | | | | | **Total confounding score: 3/6** |
| **Power** | | | | | |
| 27. | *Did the study conduct a power analysis to determine the sample size needed to detect a significant difference in effect size for one or more outcome measures?* | No | | | |
| | | | | | **Total power score: 0/1** |
| | | | | | |

| Total quality score: 17/28 |
|---|

-------------------------------------------------------------------------

| **Author (year):** Johnson (2012)<br>**Study Title:** Evaluation of the extended individual budget pilot program for families with disabled children: the family journey 1 year on<br>**Study Type:** Uncontrolled before after study | | | | | |
|---|---|---|---|---|---|
| | | Score | | | |
| | | 0 | 1 | 2 | Comment |
| **Reporting** | | | | | |
| 1. | Is the hypothesis/aim/objective of the study clearly described? | | Yes | | Objectives of the programme outlined in the Introduction of Prabhakar (2011) (The IB Process) |
| 2. | Are the main outcomes to be measured clearly described in the Introduction or Methods sections? | No | | | The outcomes to be measured are not clearly identified until the results are presented |
| 3. | Are the characteristics of the patients included in the study clearly described? | | Yes | | Reports age, nature of disability |
| 4. | Are the interventions of interest clearly described? | | Yes | | |
| 5. | Are the distributions of principal confounders in each group of subjects to be compared clearly described? | No | | | No confounders listed or controlled for |
| 6. | Are the main findings of the study clearly described? | | Yes | | |

| | | | | | |
|---|---|---|---|---|---|
| 7. | Does the study provide estimates of the random variability in the data for the main outcomes? | No | | | |
| 8. | Have all important adverse events that may be a consequence of the intervention been reported? | No | | | No adverse effects measured |
| 9. | Have the characteristics of the patients lost to follow-up been described? | No | | | |
| 10. | Have actual probability values been reported (e.g. 0.035 rather than <0.05) for the main outcomes except where the probability value is less than 0.001? | No | | | |
| | | | | | **Total reporting score: 4/11** |
| **External Validity** | | | | | |
| 11. | Were the subjects asked to participate in the study representative of the entire population from which they were recruited? | No | | | Some were randomly selected, and some were targeted. |
| 12. | Were those subjects who were prepared to participate representative of the entire population from which they were recruited? | No | | | |
| 13. | Were the staff, places, and facilities where the patients were treated, representative of the treatment the majority of patients receive? | | Yes | | |
| | | | | | **Total external validity score: 1/3** |
| **Internal Validity – Bias** | | | | | |
| 14. | Was an attempt made to blind study participants to the intervention they have received? | No | | | |
| 15. | Was an attempt made to blind those measuring outcomes of the intervention? | No | | | |

| | | | | | |
|---|---|---|---|---|---|
| 16. | If any of the results of the study were based on "data dredging", was this made clear? | | Yes | | |
| 17. | In trials and cohort studies, do the analyses adjust for different lengths of follow-up of patients, or in case control studies, is the time period between the intervention and outcomes the same for cases and controls? | | Yes | | Same length, 2 or 3 (extended pilot) years |
| 18. | Were the statistical tests used to assess the main outcomes appropriate? | Unable to determine | | | Very little information in the report on statistical tests, apart from stating that results are statistically significant. Reference to the technical annex, which we could not locate |
| 19. | Was compliance with the intervention/s reliable? | | Yes | | |
| 20. | Were the main outcome measures used accurate (valid and reliable)? | Unable to determine | | | It appears that no formally validated tools are used. No information provided on validity of results |
| | | | | | **Total bias score: 3/7** |
| **Internal Validity – Confounding (Selection bias)** | | | | | |
| 21. | Were the patients in different intervention groups (trials and cohort studies) or were the cases and controls (case-control studies) recruited from the same population? | | Yes | | Families recruited from within pilot sites |
| 22. | Were study subjects in different intervention groups (trial and cohort studies) or were the cases and controls (case-control studies) recruited over the same period of time? | | Yes | | |

| | | | | | |
|---|---|---|---|---|---|
| 23. | Were study subjects randomised to intervention groups? | No | | | |
| 24. | Was the randomised intervention assignment concealed from both patients and health care staff until recruitment was compete and irrevocable? | No | | | |
| 25. | Was there adequate adjustment for confounding in the analyses from which the main findings were drawn? | No | | | No adjustment for confounding |
| 26. | Were losses of patients to follow-up taken into account? | No | | | Only through qualitative interviews |
| | | | | | **Total confounding score: 2/6** |
| **Power** | | | | | |
| 27. | *Did the study conduct a power analysis to determine the sample size needed to detect a significant difference in effect size for one or more outcome measures?* | No | | | |
| | | | | | **Total power score: 0/1** |
| | | | | | |
| | | | | | **Total quality score: 10/28** |

-----------------------------------------------------------------------

| **Author (year):** Kim (2006)<br>**Study Title:** Comparing outcomes of persons choosing consumer-directed or agency-directed personal assistance services<br>**Study Type:** Controlled cross-sectional survey | | | |
|---|---|---|---|
| | | Score | |

| | | 0 | 1 | 2 | Comment |
|---|---|---|---|---|---|
| **Reporting** | | | | | |
| 1. | Is the hypothesis/aim/objective of the study clearly described? | | Yes | | Research questions clearly stated |
| 2. | Are the main outcomes to be measured clearly described in the Introduction or Methods sections? | | Yes | | Described in Methods section |
| 3. | Are the characteristics of the patients included in the study clearly described? | | Yes | | Reports age, gender, race, region, severity |
| 4. | Are the interventions of interest clearly described? | | Yes | | |
| 5. | Are the distributions of principal confounders in each group of subjects to be compared clearly described? | No | | | No confounders listed but sociodemographic characteristics are controlled for in regression |
| 6. | Are the main findings of the study clearly described? | | Yes | | |
| 7. | Does the study provide estimates of the random variability in the data for the main outcomes? | | Yes | | Standard error reported |
| 8. | Have all important adverse events that may be a consequence of the intervention been reported? | No | | | No adverse effects reported |
| 9. | Have the characteristics of the patients lost to follow-up been described? | No | | | No follow-up as it is as cross-sectional survey |
| 10. | Have actual probability values been reported (e.g. 0.035 rather than <0.05) for the main outcomes except where the probability value is less than 0.001? | No | | | |
| | | | | | **Total reporting score: 6/11** |
| **External Validity** | | | | | |
| 11. | Were the subjects asked to participate in the study representative of the entire population from which they were recruited? | Unable to determine | | | The sampling frame is clearly defined but the response rate was only 55.3% and no analysis was done to see if those who participated were representative |

| | | | | | |
|---|---|---|---|---|---|
| 12. | Were those subjects who were prepared to participate representative of the entire population from which they were recruited? | Unable to determine | | | |
| 13. | Were the staff, places, and facilities where the patients were treated, representative of the treatment the majority of patients receive? | | Yes | | |
| | | | | | **Total external validity score: 1/3** |
| **Internal Validity – Bias** | | | | | |
| 14. | Was an attempt made to blind study participants to the intervention they have received? | No | | | |
| 15. | Was an attempt made to blind those measuring outcomes of the intervention? | No | | | |
| 16. | If any of the results of the study were based on "data dredging", was this made clear? | | Yes | | |
| 17. | In trials and cohort studies, do the analyses adjust for different lengths of follow-up of patients, or in case control studies, is the time period between the intervention and outcomes the case for cases and controls? | Unable to determine | | | No information on how long people have been receiving the treatment |
| 18. | Were the statistical tests used to assess the main outcomes appropriate? | | Yes | | |
| 19. | Was compliance with the intervention/s reliable? | | Yes | | No non-compliance reported |
| 20. | Were the main outcome measures used accurate (valid and reliable)? | Unable to determine | | | No details on validation or reliability given |
| | | | | | **Total bias score: 3/7** |
| **Internal Validity – Confounding (Selection bias)** | | | | | |
| 21. | Were the patients in different intervention groups (trials and cohort studies) or were the cases and controls (case-control studies) recruited from the same population? | | Yes | | |

| | | | | | |
|---|---|---|---|---|---|
| 22. | Were study subjects in different intervention groups (trial and cohort studies) or were the cases and controls (case-control studies) recruited over the same period of time? | | Yes | | |
| 23. | Were study subjects randomised to intervention groups? | No | | | Researchers did not assign people to groups |
| 24. | Was the randomised intervention assignment concealed from both patients and health care staff until recruitment was compete and irrevocable? | No | | | |
| 25. | Was there adequate adjustment for confounding in the analyses from which the main findings were drawn? | | Yes | | Various socio-demographic factors controlled for in regression |
| 26. | Were losses of patients to follow-up taken into account? | No | | | |
| | | | | | **Total confounding score: 3/6** |
| **Power** | | | | | |
| 27. | *Did the study conduct a power analysis to determine the sample size needed to detect a significant difference in effect size for one or more outcome measures?* | No | | | |
| | | | | | **Total power score: 0/1** |
| | | | | | |
| | | | | | **Total quality score: 13/28** |

**Author (year):** Leuci et al. 2021
**Study Title:** Personal health budget in patients with first episode psychosis: A new rehabilitation model based on a community care system in Italy
**Study Type:** controlled cross-sectional study

| | | | | | |
|---|---|---|---|---|---|
| | | | Score | | |
| | | 0 | 1 | 2 | Comment |
| **Reporting** | | | | | |

| | | | | | |
|---|---|---|---|---|---|
| 1. | Is the hypothesis/aim/objective of the study clearly described? | | Yes | | "Starting from this background, the aim of this observational study was to assess the applicability of the PHB model in patients with first episode psychosis (FEP). In particular, we wanted to compare functional and clinical outcomes between FEP patients with and without PHB interventions along a 2-year follow-up period." |
| 2. | Are the main outcomes to be measured clearly described in the Introduction or Methods sections? | | Yes | | There are 3 outcomes: BPRS, GAF and Honos. |
| 3. | Are the characteristics of the patients included in the study clearly described? | | Yes | | Table 1 shows Sociodemographic and clinical characteristics of the total sample and the two subgroups. |
| 4. | Are the interventions of interest clearly described? | | Yes | | |
| 5. | Are the distributions of principal confounders in each group of subjects to be compared clearly described? | | Yes | | In Table 1, broken down by PHB+ or – (accept vs refuse PHB). |
| 6. | Are the main findings of the study clearly described? | | Yes | | |
| 7. | Does the study provide estimates of the random variability in the data for the main outcomes? | | Yes | | SD provided. |
| 8. | Have all important adverse events that may be a consequence of the intervention been reported? | No | | | |
| 9. | Have the characteristics of the patients lost to follow-up been described? | No (limited) | | | 15 individuals dropped out before the end of the study. We only know whether they were provided PHB or not. |
| 10. | Have actual probability values been reported (e.g., 0.035 rather than <0.05) for the main outcomes except where the probability value is less than 0.001? | | Yes | | |
| | | | | | **Total reporting score: 8/11** |
| **External Validity** | | | | | |
| 11. | Were the subjects asked to participate in the study representative of the entire population from which they were recruited? | Unable to determine | | | "FEP participants were recruited through the 'Parma-Early Psychosis' (Pr-EP) program between January 2015 and |

| | | | | | |
|---|---|---|---|---|---|
| | | | | | December 2018", then inclusion and exclusion criteria. |
| 12. | Were those subjects who were prepared to participate representative of the entire population from which they were recruited? | Unable to determine | | | We do not know how many individuals were invited to participate first. |
| 13. | Were the staff, places, and facilities where the patients were treated, representative of the treatment the majority of patients receive? | Unable to determine | | | |
| | | | | | **Total external validity score: 0/3** |
| **Internal Validity – Bias** | | | | | |
| 14. | Was an attempt made to blind study participants to the intervention they have received? | No | | | |
| 15. | Was an attempt made to blind those measuring outcomes of the intervention? | No | | | |
| 16. | If any of the results of the study were based on "data dredging", was this made clear? | | Yes | | |
| 17. | In trials and cohort studies, do the analyses adjust for different lengths of follow-up of patients, or in case control studies, is the time period between the intervention and outcomes the same for cases and controls? | | Yes | | |
| 18. | Were the statistical tests used to assess the main outcomes appropriate? | | Yes | | |
| 19. | Was compliance with the intervention/s reliable? | | Yes | | |
| 20. | Were the main outcome measures used accurate (valid and reliable)? | | Yes | | Established scales. |
| | | | | | **Total bias score: 5/7** |
| **Internal Validity – Confounding (Selection bias)** | | | | | |
| 21. | Were the patients in different intervention groups (trials and cohort studies) or were the cases and controls (case-control studies) recruited from the same population? | | Yes | | |
| 22. | Were study subjects in different intervention groups (trial and cohort studies) or were the cases and controls | | Yes | | |

| | | | | | |
|---|---|---|---|---|---|
| | (case-control studies) recruited over the same period of time? | | | | |
| 23. | Were study subjects randomised to intervention groups? | No | | | |
| 24. | Was the randomised intervention assignment concealed from both patients and health care staff until recruitment was compete and irrevocable? | No | | | |
| 25. | Was there adequate adjustment for confounding in the analyses from which the main findings were drawn? | | Yes | | Demographic characteristics controlled for in regressions |
| 26. | Were losses of patients to follow-up taken into account? | | Yes | | "Moreover, a Kaplan–Meier survival analysis to take into account the participants who dropped out before the end of the study was also carried out." |
| | | | | | **Total confounding score: 4/6** |
| **Power** | | | | | |
| 27. | *Did the study conduct a power analysis to determine the sample size needed to detect a significant difference in effect size for one or more outcome measures?* | | | | |
| | | | | | **Total power score: 0/1** |
| | | | | | |
| | | | | | **Total quality score: 17/28** |

| | | | | | |
|---|---|---|---|---|---|
| **Author (year):** Pelizza et al. 2022<br>**Study Title:** Personal Health Budget as a new rehabilitation model for severe mental illness within a caring community: An Italian evaluation study of beneficial effects.<br>**Study Type:** descriptive cohort study | | | | | |
| | | Score | | | |
| | | 0 | 1 | 2 | Comment |
| **Reporting** | | | | | |
| 1. | Is the hypothesis/aim/objective of the study clearly described? | | Yes | | The two objectives of this paper are describe in the introduction: "(a) to offer a general description of the Italian PHB methodology implemented in the Parma |

| | | | | | |
|---|---|---|---|---|---|
| | | | | | Department of Mental Health since 2016 and (b) to provide preliminary data on the beneficial effects of such PHB intervention in adults with SMI. Specifically, we compared functional and clinical outcome indicators across a 2-year follow-up period.” |
| 2. | Are the main outcomes to be measured clearly described in the Introduction or Methods sections? | | Yes | | There are 3 functional and clinical outcome indicators (BPRS, GAF, HONOS), briefly described. |
| 3. | Are the characteristics of the patients included in the study clearly described? | | Yes | | Inclusion criteria and descriptive statistics provided. |
| 4. | Are the interventions of interest clearly described? | | Yes | | There is a brief section on Personal Health Budgets and then detailed section on PHB in Italy, describing what has been done so far. Then, there is a precise description of the procedure, including the different modalities of the intervention (three axis: social, health, employment). |
| 5. | Are the distributions of principal confounders in each group of subjects to be compared clearly described? | no | | | No control group |
| 6. | Are the main findings of the study clearly described? | | yes | | |
| 7. | Does the study provide estimates of the random variability in the data for the main outcomes? | | yes | | |
| 8. | Have all important adverse events that may be a consequence of the intervention been reported? | no | | | Unmet needs, burden etc. not mentioned |
| 9. | Have the characteristics of the patients lost to follow-up been described? | no | | | 10 were lost and among them, 5 because unreachable. No more information. |
| 10. | Have actual probability values been reported (e.g., 0.035 rather than <0.05) for the main outcomes except where the probability value is less than 0.001? | | yes | | |
| | | | | | **Total reporting score: 7/11** |

| | | | | | |
|---|---|---|---|---|---|
| **External Validity** | | | | | |
| 11. | Were the subjects asked to participate in the study representative of the entire population from which they were recruited? | Unable to determine | | | "Data were retrospectively collected at baseline and at the follow-up routine assessments of patients with SMI recruited in one of the six adult mental health services of the Parma Department of Mental Health from January 2016 to December 2018. Participants were recruited in community-based mental health settings" with then inclusion and exclusion criteria. Limited sample size. |
| 12. | Were those subjects who were prepared to participate representative of the entire population from which they were recruited? | | Yes | | "the PHB intervention was consecutively proposed to 150 SMI patients but only 137 agreed" : Response rate (137/150) |
| 13. | Were the staff, places, and facilities where the patients were treated, representative of the treatment the majority of patients receive? | | Yes | | |
| | | | | | **Total external validity score: 2/3** |
| **Internal Validity – Bias** | | | | | |
| 14. | Was an attempt made to blind study participants to the intervention they have received? | No | | | |
| 15. | Was an attempt made to blind those measuring outcomes of the intervention? | No | | | |
| 16. | If any of the results of the study were based on "data dredging", was this made clear? | | yes | | |
| 17. | In trials and cohort studies, do the analyses adjust for different lengths of follow-up of patients, or in case control studies, is the time period between the intervention and outcomes the same for cases and controls? | | Yes | | "Baseline assessments thus corresponded to PHB intervention entry" |
| 18. | Were the statistical tests used to assess the main outcomes appropriate? | | Yes | | Friedman test |
| 19. | Was compliance with the intervention/s reliable? | | Yes | | |

| | | | | | |
|---|---|---|---|---|---|
| 20. | Were the main outcome measures used accurate (valid and reliable)? | | Yes | | Established scales |
| | | | | | **Total bias score: 5/7** |
| **Internal Validity – Confounding (Selection bias)** | | | | | |
| 21. | Were the patients in different intervention groups (trials and cohort studies) or were the cases and controls (case-control studies) recruited from the same population? | | yes | | |
| 22. | Were study subjects in different intervention groups (trial and cohort studies) or were the cases and controls (case-control studies) recruited over the same period of time? | | Yes | | |
| 23. | Were study subjects randomised to intervention groups? | Unable to determine | | | There is a multiaxis PHB, provided to 55 individuals and then, others were provided with different PHB approaches but is unclear who has been provided to PHB housing and who has been assigned to PHB social. No mention of randomisation. |
| 24. | Was the randomised intervention assignment concealed from both patients and health care staff until recruitment was compete and irrevocable? | Unable to determine | | | |
| 25. | Was there adequate adjustment for confounding in the analyses from which the main findings were drawn? | | Yes | | Demographic characteristics controlled for in regressions |
| 26. | Were losses of patients to follow-up taken into account? | | Yes | | 10 were lost |
| | | | | | **Total confounding score: 4/6** |
| **Power** | | | | | |
| 27. | *Did the study conduct a power analysis to determine the sample size needed to detect a significant difference in effect size for one or more outcome measures?* | No | | | |
| | | | | | **Total power score: 0/1** |
| | | | | | |
| | | | | | **Total quality score: 18/28** |

| | | | | | |
|---|---|---|---|---|---|
| **Author (year):** Thom (2019)<br>**Study Title:** Evaluation of the Integrated Personal Commissioning (IPC) Programme: final evaluation report<br>**Study Type:** Uncontrolled before-after study | | | | | |
| | | Score | | | |
| | | 0 | 1 | 2 | Comment |
| **Reporting** | | | | | |
| 1. | Is the hypothesis/aim/objective of the study clearly described? | | Yes | | *"The key research question to answer would be: how far has IPC been a positive and different experience from 'normal' care and support planning?"* |
| 2. | Are the main outcomes to be measured clearly described in the Introduction or Methods sections? | | Yes | | A list of the tools used to measure outcomes is provide in the Impact Evaluation description |
| 3. | Are the characteristics of the patients included in the study clearly described? | | Yes | | Gender, age, health condition, ethnicity, employment all reported. |
| 4. | Are the interventions of interest clearly described? | | Yes | | The integration of health and social budgets is described |
| 5. | Are the distributions of principal confounders in each group of subjects to be compared clearly described? | No | | | No confounders listed |
| 6. | Are the main findings of the study clearly described? | | Yes | | All results presented in tables and discussed in the text |
| 7. | Does the study provide estimates of the random variability in the data for the main outcomes? | | Yes | | Standard deviations reported |
| 8. | Have all important adverse events that may be a consequence of the intervention been reported? | | Yes | | Deterioration in quality of life considered |

| | | | | | |
|---|---|---|---|---|---|
| 9. | Have the characteristics of the patients lost to follow-up been described? | No | | | |
| 10. | Have actual probability values been reported (e.g. 0.035 rather than <0.05) for the main outcomes except where the probability value is less than 0.001? | | Yes | | |
| | | | | | **Total reporting score: 8/11** |
| **External Validity** | | | | | |
| 11. | Were the subjects asked to participate in the study representative of the entire population from which they were recruited? | Unable to determine | | | The process of selecting the participants is poorly described. It was certainly not random |
| 12. | Were those subjects who were prepared to participate representative of the entire population from which they were recruited? | Unable to determine | | | No information provided on people who were offered the change to participate and those that accepted or refused |
| 13. | Were the staff, places, and facilities where the patients were treated, representative of the treatment the majority of patients receive? | | Yes | | No specialist treatments involved |
| | | | | | **Total external validity score: 1/3** |
| **Internal Validity – Bias** | | | | | |
| 14. | Was an attempt made to blind study participants to the intervention they have received? | No | | | |
| 15. | Was an attempt made to blind those measuring outcomes of the intervention? | No | | | |
| 16. | If any of the results of the study were based on “data dredging”, was this made clear? | | Yes | | Many unforeseen issues with the programme are reported and the need to change the methods of analysis are clearly explained |
| 17. | In trials and cohort studies, do the analyses adjust for different lengths of follow-up of patients, or in | No | | | Follow-up interviews were conducted between 3 and 16 months after |

| | | | | | |
|---|---|---|---|---|---|
| | case control studies, is the time period between the intervention and outcomes the case for cases and controls? | | | | baseline, no adjustment for this is reported |
| 18. | Were the statistical tests used to assess the main outcomes appropriate? | | Yes | | |
| 19. | Was compliance with the intervention/s reliable? | | Yes | | No specific non-compliance reported |
| 20. | Were the main outcome measures used accurate (valid and reliable)? | | Yes | | Validated tools used |
| | | | | | **Total bias score: 4/7** |
| **Internal Validity – Confounding (Selection bias)** | | | | | |
| 21. | Were the patients in different intervention groups (trials and cohort studies) or were the cases and controls (case-control studies) recruited from the same population? | Unable to determine | | | Identification of potential participants was done by the "practitioner", but no details are provide on the specific source population or on how potential participants were actually identified |
| 22. | Were study subjects in different intervention groups (trial and cohort studies) or were the cases and controls (case-control studies) recruited over the same period of time? | No | | | Some sites joined at later time, up to 1.5 years later |
| 23. | Were study subjects randomised to intervention groups? | No | | | |
| 24. | Was the randomised intervention assignment concealed from both patients and health care staff until recruitment was compete and irrevocable? | No | | | |
| 25. | Was there adequate adjustment for confounding in the analyses from which the main findings were drawn? | No | | | No adjustment for confounding |
| 26. | Were losses of patients to follow-up taken into account? | No | | | |
| | | | | | **Total confounding score: 0/6** |

| | | | | | |
|---|---|---|---|---|---|
| **Power** | | | | | |
| 27. | *Did the study conduct a power analysis to determine the sample size needed to detect a significant difference in effect size for one or more outcome measures?* | No | | | |
| | | | | | **Total power score: 0/1** |
| | | | | | |
| | | | | | **Total quality score: 13/28** |

-----------------------------------------------------------------------

| | | | | | |
|---|---|---|---|---|---|
| **Author (year):** Vinton (2010)<br>**Study Title:** Caregivers' perceptions of a consumer-directed care program for adults with development disabilities<br>**Study Type:** Uncontrolled before-after study | | | | | |
| | | Score | | | |
| | | 0 | 1 | 2 | Comment |
| **Reporting** | | | | | |
| 1. | Is the hypothesis/aim/objective of the study clearly described? | | Yes | | Evaluation aim stated in introduction |
| 2. | Are the main outcomes to be measured clearly described in the Introduction or Methods sections? | | Yes | | Stated in Methods section |
| 3. | Are the characteristics of the patients included in the study clearly described? | | Yes | | Reports age, gender, race, living arrangements, diagnosis, level of need |
| 4. | Are the interventions of interest clearly described? | | Yes | | Intervention described in the Methods section |
| 5. | Are the distributions of principal confounders in each group of subjects to be compared clearly described? | No | | | No confounders reported |
| 6. | Are the main findings of the study clearly described? | | Yes | | |

| | | | | | |
|---|---|---|---|---|---|
| 7. | Does the study provide estimates of the random variability in the data for the main outcomes? | | Yes | | Standard deviation and confidence intervals reported |
| 8. | Have all important adverse events that may be a consequence of the intervention been reported? | No | | | No adverse effects reported |
| 9. | Have the characteristics of the patients lost to follow-up been described? | No | | | Only characteristics of people with follow-up survey responses are reported |
| 10. | Have actual probability values been reported (e.g. 0.035 rather than <0.05) for the main outcomes except where the probability value is less than 0.001? | | Yes | | |
| | | | | | **Total reporting score: 7/11** |
| **External Validity** | | | | | |
| 11. | Were the subjects asked to participate in the study representative of the entire population from which they were recruited? | Unable to determine | | | No information provided on how they were selected other than being referred by staff |
| 12. | Were those subjects who were prepared to participate representative of the entire population from which they were recruited? | Unable to determine | | | |
| 13. | Were the staff, places, and facilities where the patients were treated, representative of the treatment the majority of patients receive? | | Yes | | |
| | | | | | **Total external validity score: 1/3** |
| **Internal Validity – Bias** | | | | | |
| 14. | Was an attempt made to blind study participants to the intervention they have received? | No | | | |
| 15. | Was an attempt made to blind those measuring outcomes of the intervention? | No | | | |
| 16. | If any of the results of the study were based on "data dredging", was this made clear? | | Yes | | No unplanned analysis or subgroup analysis reported |

| | | | | | |
|---|---|---|---|---|---|
| 17. | In trials and cohort studies, do the analyses adjust for different lengths of follow-up of patients, or in case control studies, is the time period between the intervention and outcomes the case for cases and controls? | | Yes | | All respondents had 6 months of data |
| 18. | Were the statistical tests used to assess the main outcomes appropriate? | | Yes | | Paired t-tests |
| 19. | Was compliance with the intervention/s reliable? | | Yes | | No non-compliance reported |
| 20. | Were the main outcome measures used accurate (valid and reliable)? | Unable to determine | | | No formal instruments used; no validity tests performed |
| | | | | | **Total bias score: 4/7** |
| **Internal Validity – Confounding (Selection bias)** | | | | | |
| 21. | Were the patients in different intervention groups (trials and cohort studies) or were the cases and controls (case-control studies) recruited from the same population? | Unable to determine | | | |
| 22. | Were study subjects in different intervention groups (trial and cohort studies) or were the cases and controls (case-control studies) recruited over the same period of time? | No | | | Recruitment was staggered |
| 23. | Were study subjects randomised to intervention groups? | No | | | |
| 24. | Was the randomised intervention assignment concealed from both patients and health care staff until recruitment was compete and irrevocable? | No | | | |
| 25. | Was there adequate adjustment for confounding in the analyses from which the main findings were drawn? | No | | | |
| 26. | Were losses of patients to follow-up taken into account? | | Yes | | Only people with 6 months of data were included |

| | | | | | |
|---|---|---|---|---|---|
| **Total confounding score: 1/6** | | | | | |
| **Power** | | | | | |
| 27. | *Did the study conduct a power analysis to determine the sample size needed to detect a significant difference in effect size for one or more outcome measures?* | No | | | |
| **Total power score: 0/1** | | | | | |
| | | | | | |
| **Total quality score: 13/28** | | | | | |

-----------------------------------------------------------------------

| | | | | | |
|---|---|---|---|---|---|
| **Author (year):** Wiener (2007)<br>**Study Title:** Are consumer-directed home care beneficiaries satisfied? Evidence from Washington State<br>**Study Type:** Controlled cross-sectional survey | | | | | |
| | | Score | | | |
| | | 0 | 1 | 2 | Comment |
| **Reporting** | | | | | |
| 1. | Is the hypothesis/aim/objective of the study clearly described? | | Yes | | Goal of the study is clearly stated |
| 2. | Are the main outcomes to be measured clearly described in the Introduction or Methods sections? | | Yes | | Reported in Methods section |
| 3. | Are the characteristics of the patients included in the study clearly described? | | Yes | | Reports age, race, gender, health and functioning status, residence |
| 4. | Are the interventions of interest clearly described? | | Yes | | Control and treatment described |
| 5. | Are the distributions of principal confounders in each group of subjects to be compared clearly described? | No | | | No list of confounders provided, but all participant characteristics controlled for in regression |
| 6. | Are the main findings of the study clearly described? | | Yes | | |

| | | | | | |
|---|---|---|---|---|---|
| 7. | Does the study provide estimates of the random variability in the data for the main outcomes? | No | | | |
| 8. | Have all important adverse events that may be a consequence of the intervention been reported? | | Yes | | Poor health, pain, unmet needs.... |
| 9. | Have the characteristics of the patients lost to follow-up been described? | No | | | No relevant for cross-sectional survey |
| 10. | Have actual probability values been reported (e.g. 0.035 rather than <0.05) for the main outcomes except where the probability value is less than 0.001? | No | | | |
| | | | | | **Total reporting score: 6/11** |
| **External Validity** | | | | | |
| 11. | Were the subjects asked to participate in the study representative of the entire population from which they were recruited? | Unable to determine | | | The population used to sample from was itself a sample from a previous study that I cannot locate. |
| 12. | Were those subjects who were prepared to participate representative of the entire population from which they were recruited? | Unable to determine | | | How the sample was taken is not clear, "the state drew the initial sample", not stated how it a drawn |
| 13. | Were the staff, places, and facilities where the patients were treated, representative of the treatment the majority of patients receive? | | Yes | | |
| | | | | | **Total external validity score: 1/3** |
| **Internal Validity – Bias** | | | | | |
| 14. | Was an attempt made to blind study participants to the intervention they have received? | No | | | |
| 15. | Was an attempt made to blind those measuring outcomes of the intervention? | No | | | |
| 16. | If any of the results of the study were based on "data dredging", was this made clear? | | Yes | | |

| | | | | | |
|---|---|---|---|---|---|
| 17. | In trials and cohort studies, do the analyses adjust for different lengths of follow-up of patients, or in case control studies, is the time period between the intervention and outcomes the case for cases and controls? | Unable to determine | | | It is not stated how long people have been in receipt of treatment option |
| 18. | Were the statistical tests used to assess the main outcomes appropriate? | | Yes | | |
| 19. | Was compliance with the intervention/s reliable? | | Yes | | No non-compliance reported |
| 20. | Were the main outcome measures used accurate (valid and reliable)? | | Yes | | Cronbach's alpha reported |
| | | | | | **Total bias score: 4/7** |
| **Internal Validity – Confounding (Selection bias)** | | | | | |
| 21. | Were the patients in different intervention groups (trials and cohort studies) or were the cases and controls (case-control studies) recruited from the same population? | | Yes | | |
| 22. | Were study subjects in different intervention groups (trial and cohort studies) or were the cases and controls (case-control studies) recruited over the same period of time? | | Yes | | |
| 23. | Were study subjects randomised to intervention groups? | No | | | Researchers did not assign people to groups |
| 24. | Was the randomised intervention assignment concealed from both patients and health care staff until recruitment was compete and irrevocable? | No | | | |
| 25. | Was there adequate adjustment for confounding in the analyses from which the main findings were drawn? | | Yes | | Demographic characteristics controlled for in regression |
| 26. | Were losses of patients to follow-up taken into account? | No | | | Not relevant |

| | | | | | |
|---|---|---|---|---|---|
| **Total confounding score: 3/6** | | | | | |
| **Power** | | | | | |
| 27. | *Did the study conduct a power analysis to determine the sample size needed to detect a significant difference in effect size for one or more outcome measures?* | No | | | |
| **Total power score: 0/1** | | | | | |
| | | | | | |
| **Total quality score: 14/28** | | | | | |

-----------------------------------------------------------------------

**Author (year):** Woolham (2013)
**Study Title:** The costs and benefits of personal budgets for older people: evidence from a single local authority
**Study Type:** Controlled cross-sectional survey

| | | Score | | | |
|---|---|---|---|---|---|
| | | 0 | 1 | 2 | Comment |
| **Reporting** | | | | | |
| 1. | Is the hypothesis/aim/objective of the study clearly described? | Yes | | | |
| 2. | Are the main outcomes to be measured clearly described in the Introduction or Methods sections? | | Yes | | Presented in Methods section |
| 3. | Are the characteristics of the patients included in the study clearly described? | | Yes | | Reports age, gender, ethnic group, disability type |
| 4. | Are the interventions of interest clearly described? | | Yes | | |
| 5. | Are the distributions of principal confounders in each group of subjects to be compared clearly described? | No | | | No list of confounders reported |

| | | | | | |
|---|---|---|---|---|---|
| 6. | Are the main findings of the study clearly described? | | Yes | | Provided through narrative text and scatter plots |
| 7. | Does the study provide estimates of the random variability in the data for the main outcomes? | | Yes | | Standard deviation for health outcomes reported |
| 8. | Have all important adverse events that may be a consequence of the intervention been reported? | | Yes | | Psychological well-being and mental distress |
| 9. | Have the characteristics of the patients lost to follow-up been described? | No | | | Not relevant for cross-sectional study |
| 10. | Have actual probability values been reported (e.g. 0.035 rather than <0.05) for the main outcomes except where the probability value is less than 0.001? | | Yes | | |
| | | | | | **Total reporting score: 8/11** |
| **External Validity** | | | | | |
| 11. | Were the subjects asked to participate in the study representative of the entire population from which they were recruited? | Unable to determine | | | There is no information provided on how the sample was selected |
| 12. | Were those subjects who were prepared to participate representative of the entire population from which they were recruited? | Unable to determine | | | Response rates reported but no validation on if these people are representative is provided |
| 13. | Were the staff, places, and facilities where the patients were treated, representative of the treatment the majority of patients receive? | | Yes | | |
| | | | | | **Total external validity score: 1/3** |
| **Internal Validity – Bias** | | | | | |
| 14. | Was an attempt made to blind study participants to the intervention they have received? | No | | | |
| 15. | Was an attempt made to blind those measuring outcomes of the intervention? | No | | | |

| | | | | | |
|---|---|---|---|---|---|
| 16. | If any of the results of the study were based on “data dredging”, was this made clear? | | Yes | | Subgroup analysis performed but the reason is clearly stated |
| 17. | In trials and cohort studies, do the analyses adjust for different lengths of follow-up of patients, or in case control studies, is the time period between the intervention and outcomes the case for cases and controls? | Unable to determine | | | No information on how long people have been receiving treatment |
| 18. | Were the statistical tests used to assess the main outcomes appropriate? | | Yes | | Paired t tests |
| 19. | Was compliance with the intervention/s reliable? | | Yes | | No non-compliance reported |
| 20. | Were the main outcome measures used accurate (valid and reliable)? | | Yes | | ADL and GHQ |
| | | | | | **Total bias score: 4/7** |
| **Internal Validity – Confounding (Selection bias)** | | | | | |
| 21. | Were the patients in different intervention groups (trials and cohort studies) or were the cases and controls (case-control studies) recruited from the same population? | | Yes | | All recruited from the same English local authority |
| 22. | Were study subjects in different intervention groups (trial and cohort studies) or were the cases and controls (case-control studies) recruited over the same period of time? | | Yes | | |
| 23. | Were study subjects randomised to intervention groups? | No | | | Researchers didn’t assign people to groups |
| 24. | Was the randomised intervention assignment concealed from both patients and health care staff until recruitment was compete and irrevocable? | No | | | |
| 25. | Was there adequate adjustment for confounding in the analyses from which the main findings were drawn? | No | | | |

| | | | | | |
|---|---|---|---|---|---|
| 26. | Were losses of patients to follow-up taken into account? | No | | | Not relevant for cross-sectional study |
| | | | | | **Total confounding score: 2/6** |
| **Power** | | | | | |
| 27. | *Did the study conduct a power analysis to determine the sample size needed to detect a significant difference in effect size for one or more outcome measures?* | No | | | |
| | | | | | **Total power score: 0/1** |
| | | | | | |
| | | | | | **Total quality score: 15/28** |

| | | | | | |
|---|---|---|---|---|---|
| **Author (year):** Dale et al., 2004<br>**Study Title:** Medicaid Costs Under Consumer Direction for Florida Children with Developmental Disabilities<br>**Study Type:** Randomised controlled trial | | | | | |
| | | Score | | | |
| | | 0 | 1 | 2 | Comment |
| **Reporting** | | | | | |
| 1. | Is the hypothesis/aim/objective of the study clearly described? | | Yes | | In the executive summary, p'V.' Section describing the hypotheses p.9 |
| 2. | Are the main outcomes to be measured clearly described in the Introduction or Methods sections? | | Yes | | Outcomes are drawn from Medicaid claim and described p.12 in a subsection. |
| 3. | Are the characteristics of the patients included in the study clearly described? | | Yes | | Control variables from the baseline survey include the consumers' demographic characteristics, measures of health and functioning. |

| | | | | | |
|---|---|---|---|---|---|
| | | | | | Table 1 provides summary stats, see p. 15/16 |
| 4. | Are the interventions of interest clearly described? | | Yes | | C&C clearly described in the background part including a specific subsection on Cash and Counseling for Florida Children |
| 5. | Are the distributions of principal confounders in each group of subjects to be compared clearly described? | | Partially | | Significant differences between treatment and control groups at baseline are drawn, see Table 1. Heterogeneity analysis is provided showing that effects on outcomes might vary depending on subgroups. Controls are included in regression models. |
| 6. | Are the main findings of the study clearly described? | | Yes | | |
| 7. | Does the study provide estimates of the random variability in the data for the main outcomes? | No | | | No SE or CI |
| 8. | Have all important adverse events that may be a consequence of the intervention been reported? | | Yes | | Including outcomes such as children's use of any inpatient services, the number of inpatient days, inpatient expenditures, or the likelihood of having an emergency room visit. See Table 5 for an example. |
| 9. | Have the characteristics of the patients lost to follow-up been described? | No | | | Not really mentioned. In a footnote authors briefly discuss |

| | | | | | |
|---|---|---|---|---|---|
| | | | | | ‘**disenrollment from the program** ’ about a quarter |
| 10. | Have actual probability values been reported (e.g., 0.035 rather than <0.05) for the main outcomes except where the probability value is less than 0.001? | | Yes | | |
| **Total reporting score: 8/11** | | | | | |
| | | | | | |
| 11. | Were the subjects asked to participate in the study representative of the entire population from which they were recruited? | No | | | Children with developmental disabilities in Florida between 3 and 17 |
| 12. | Were those subjects who were prepared to participate representative of the entire population from which they were recruited? | No | | | Same in Florida |
| 13. | Were the staff, places, and facilities where the patients were treated, representative of the treatment the majority of patients receive? | No | | | Specificities in the C&C implementation in Florida |
| **Total external validity score: 0/3** | | | | | |
| | | | | | |
| 14. | Was an attempt made to blind study participants to the intervention they have received? | No | | | See p.6. Parents know what would be the amount of the allowance for their children if assigned to the treatment group |
| 15. | Was an attempt made to blind those measuring outcomes of the intervention? | No | | | |
| 16. | If any of the results of the study were based on “data dredging”, was this made clear? | | Yes | | No unplanned analysis reported |

| | | | | | |
|---|---|---|---|---|---|
| 17. | In trials and cohort studies, do the analyses adjust for different lengths of follow-up of patients, or in case control studies, is the time period between the intervention and outcomes the same for cases and controls? | | Partially | | Enrollment between June 2000 and August 2001.<br>Medicaid claims data for the first 24 months are used: difference made between first postenrollment year and second postenrollement year. Time period of enrollment controlled for in analysis |
| 18. | Were the statistical tests used to assess the main outcomes appropriate? | | Yes | | OLS, Tobit and Logit regression models |
| 19. | Was compliance with the intervention/s reliable? | | Yes | | |
| 20. | Were the main outcome measures used accurate (valid and reliable)? | | Yes | | Clearly described |
| | | | | | **Total bias score: 5/7** |
| | | | | | |
| 21. | Were the patients in different intervention groups (trials and cohort studies) or were the cases and controls (case-control studies) recruited from the same population? | | Yes | | Children from Florida, same age and '**Before enrollment, the children in our sample were receiving a wide variety of benefits through the waiver program'** see p.5 |
| 22. | Were study subjects in different intervention groups (trial and cohort studies) or were the cases and controls (case-control studies) recruited over the same period of time? | | Yes | | Between June 2000 and August 2001. Then randomly assigned to a group. |
| 23. | Were study subjects randomised to intervention groups? | | Yes | | See p.7 |

| | | | | | |
|---|---|---|---|---|---|
| 24. | Was the randomised intervention assignment concealed from both patients and health care staff until recruitment was compete and irrevocable? | | Yes | | See p.7 '(…) **interview with parents who enrolled their child in the demonstration, then randomly assigned each child to the treatment or the control group'** |
| 25. | Was there adequate adjustment for confounding in the analyses from which the main findings were drawn? | | Yes | | Baseline differences between groups controlled for in analysis |
| 26. | Were losses of patients to follow-up taken into account? | | Yes | | Comparisons are made for people with all observations. Still 1002 children in the second year post enrollment. Sample size still the same. |
| | | | | | **Total confounding score: 6/6** |
| **Power** | | | | | |
| 27. | *Did the study conduct a power analysis to determine the sample size needed to detect a significant difference in effect size for one or more outcome measures?* | No | | | No statistical power conducted |
| | | | | | **Total power score: 0/1** |
| | | | | | |
| | | | | | **Total quality score: 19/28** |

------------------------------------------------------------------------

**Author (year):** Dale et al., 2005
**Study Title:** The Effect of Cash and Counseling on Medicaid and Medicare Costs: Findings for Adults in Three States
**Study Type:** Randomised controlled trial

| | | | | | |
|---|---|---|---|---|---|
| **Information:** 'The current report is the third in a set of three. These reports compare treatment and control group members, using Medicaid and Medicare data describing the cost of personal care and other covered services measured during the year after random assignment' | | | | | |
| | | Score | | | |
| | | 0 | 1 | 2 | Comment |
| **Reporting** | | | | | |
| 1. | Is the hypothesis/aim/objective of the study clearly described? | | Yes | | **'This report compares results from all three demonstration 3 programs to examine how consumer direction for adults affects Medicaid and Medicare service use and costs.'** P.2 and description of the expected effects p.11, kind of hypotheses |
| 2. | Are the main outcomes to be measured clearly described in the Introduction or Methods sections? | | Yes | | Described in a sub section p.15 |
| 3. | Are the characteristics of the patients included in the study clearly described? | | Yes | | treatment and control group members' baseline characteristics described (a table of summary stats for each state: 1a, 1b, 1c) |
| 4. | Are the interventions of interest clearly described? | | Yes | | With a clear description of the differences between the implementation of C&C in the three states. See appendix C also for summary tables. |
| 5. | Are the distributions of principal confounders in each group of subjects to be compared clearly described? | | Partially | | Significant differences between treatment and control groups at baseline are drawn (Tables 1a, 1b, |

| | | | | | |
|---|---|---|---|---|---|
| | | | | | 1c): '**As would be expected under random assignment, there were few significant differences between treatment and control group members' baseline characteristics**'. Heterogeneity analysis is provided showing that effects on outcomes may vary (elderly vs non elderly for instance). '**All the models controlled for the sample members' baseline measures of demographic characteristics** ' p. 18: controls are incuded in regression models |
| 6. | Are the main findings of the study clearly described? | | Yes | | From p.23 and further |
| 7. | Does the study provide estimates of the random variability in the data for the main outcomes? | No | | | No SE or CI |
| 8. | Have all important adverse events that may be a consequence of the intervention been reported? | | Partially | | Controls such as '**measures of health and functioning, and measures of unmet need for personal care** ' are included. Additional analysis on quality indicators of the program are done. Discussion on adverse effects p.77 |
| 9. | Have the characteristics of the patients lost to follow-up been described? | No | | | For some sites, and groups (elderly vs nonelderly), sample sizes are reported for first and second follow- |

| | | | | | |
|---|---|---|---|---|---|
| | | | | | up years or for follow-up years but no information on characteristics of patients lost. Attrition due to death discussed: '**First-year mortality rates for treatment and control group members were 14 and 12 percent, respectively, in Arkansas; 7 and 8 percent, respectively, in Florida; and 7 percent for both groups in New Jersey**.' P.17 |
| 10. | Have actual probability values been reported (e.g. 0.035 rather than <0.05) for the main outcomes except where the probability value is less than 0.001? | | Yes | | |
| | | | | | **Total reporting score: 8/11** |
| **External Validity** | | | | | |
| 11. | Were the subjects asked to participate in the study representative of the entire population from which they were recruited? | Unable | | | Within a site yes but not between sites: '**in Florida, to be eligible for the demonstration, beneficiaries had to already be receiving some costed-out waiver services. In New Jersey, beneficiaries had to have applied for agency PCS and been assessed as eligible to receive them. Only these people were invited to participate in the program.4 Arkansas, however, allowed** |

| | | | | | |
|---|---|---|---|---|---|
| | | | | | **anyone eligible for Medicaid personal care to enroll and used a letter from the governor to inform all Medicaid beneficiaries in the state about this option.**' And also '**None of the states screened eligible consumers for appropriateness**.' Results are also reported by sites. |
| 12. | Were those subjects who were prepared to participate representative of the entire population from which they were recruited? | Unable | | | No details on how participants are selected for this study |
| 13. | Were the staff, places, and facilities where the patients were treated, representative of the treatment the majority of patients receive? | | Yes | | Differences between sites but results are also reported by sites, see p.12 |
| | | | | | **Total external validity score: 1/3** |
| **Internal Validity – Bias** | | | | | |
| 14. | Was an attempt made to blind study participants to the intervention they have received? | No | | | |
| 15. | Was an attempt made to blind those measuring outcomes of the intervention? | No | | | |
| 16. | If any of the results of the study were based on "data dredging", was this made clear? | | Yes | | |
| 17. | In trials and cohort studies, do the analyses adjust for different lengths of follow-up of patients, or in case control studies, is the time | | Yes | | Results are also reported by year of postenrollement. Restricted analysis to a cohort of early enrollees is done, |

| | | | | | |
|---|---|---|---|---|---|
| | period between the intervention and outcomes the same for cases and controls? | | | | see p.18 and tables A2a to c in the appendix. |
| 18. | Were the statistical tests used to assess the main outcomes appropriate? | | Yes | | OLS, Tobit and Logit models |
| 19. | Was compliance with the intervention/s reliable? | | Yes | | No reported non-compliance |
| 20. | Were the main outcome measures used accurate (valid and reliable)? | | Yes | | Refers to established measures: '**Medicaid expenditure measures were drawn from Medicaid claims data supplied by each state, and Medicare expenditure measures were drawn from Medicare claims data** ' p.15 |
| | | | | | **Total bias score: 5/7** |
| **Internal Validity – Confounding (Selection bias)** | | | | | |
| 21. | Were the patients in different intervention groups (trials and cohort studies) or were the cases and controls (case-control studies) recruited from the same population? | | Yes | | From different population between sites because enrollement conditions are different but within a site, yes: '**Demonstration enrollment, which occurred between December 1998 and July 2002, was open to interested beneficiaries eligible for PCS under their state Medicaid plan (in Arkansas and New Jersey) or under a waiver (in Florida). After a baseline survey, enrollees were randomly assigned to (...).**' see Executive summary. |

| | | | | | |
|---|---|---|---|---|---|
| 22. | Were study subjects in different intervention groups (trial and cohort studies) or were the cases and controls (case-control studies) recruited over the same period of time? | | Yes | | Although this varied by site, but site results are reported separately: '**this cohort includes those who enrolled in Arkansas's IndependentChoices before May 2000, Florida's CDC before October 2001, or New Jersey's Personal Preference program before January 2002.**' P.14 |
| 23. | Were study subjects randomised to intervention groups? | | Yes | | |
| 24. | Was the randomised intervention assignment concealed from both patients and health care staff until recruitment was compete and irrevocable? | | Yes | | '**<u>After a baseline survey</u>, enrollees were randomly assigned to direct their own personal assistance as Cash and Counseling consumers (the treatment group) or to receive services as usual from agencies (the control group)**': baseline interviews were conducted before assignement. See executive summary |
| 25. | Was there adequate adjustment for confounding in the analyses from which the main findings were drawn? | | Yes | | '**All the models controlled for the sample members' baseline measures of demographic characteristics**', p.18 but result tables incuding controls are not provided. |
| 26. | Were losses of patients to follow-up taken into account? | | Yes | | Only subjects with complete data were included because sample sizes |

| | | | | | |
|---|---|---|---|---|---|
| | | | | | still the same from first to second year postenrollment |
| **Total confounding score: 6/6** | | | | | |
| **Power** | | | | | |
| 27. | *Did the study conduct a power analysis to determine the sample size needed to detect a significant difference in effect size for one or more outcome measures?* | | Yes | | **'the statistical power to detect impacts of X percent '** see appendix, table A3 |
| **Total power score: 1/1** | | | | | |
| | | | | | |
| **Total quality score: 21/28** | | | | | |

---------------------------------------------------------------------

| | | | | | |
|---|---|---|---|---|---|
| **Author (year):** Carlson et al., 2005<br>**Study Title:** EFFECT OF CONSUMER DIRECTION ON ADULTS' PERSONAL CARE AND WELL-BEING IN ARKANSAS, NEW JERSEY AND FLORIDA<br>**Study Type:** Randomised controlled trial | | | | | |
| | | Score | | | |
| | | 0 | 1 | 2 | Comment |
| **Reporting** | | | | | |
| 1. | Is the hypothesis/aim/objective of the study clearly described? | | Yes | | See executive summary p.vi and Introduction p.2. Expected effects from p.10. |
| 2. | Are the main outcomes to be measured clearly described in the Introduction or Methods sections? | | Yes | | Subsection p.13 |
| 3. | Are the characteristics of the patients included in the study clearly described? | | Yes | | p.12, p17 and appendix, see table A.3 |

| | | | | | |
|---|---|---|---|---|---|
| 4. | Are the interventions of interest clearly described? | | yes | | Clear description of C&C and the variations in program features by state. From p.3 and further. Also see Table 1. Summarising key features of C&C by state |
| 5. | Are the distributions of principal confounders in each group of subjects to be compared clearly described? | | Partially | | Differences between T and C at baseline, see Table A.2. in appendices but no clear mention of a list of confounders. Results reported separately for subgroups: elderly vs nonelderly |
| 6. | Are the main findings of the study clearly described? | | yes | | From p.23 |
| 7. | Does the study provide estimates of the random variability in the data for the main outcomes? | No | | | No SE or CI |
| 8. | Have all important adverse events that may be a consequence of the intervention been reported? | | yes | | The frequency of unmet needs, and the incidence of adverse health events are examined. '**We also asked factual questions about disability- or health-related adverse events and health problems the beneficiary might have experienced.**' See Table 18 and 29 for results. |
| 9. | Have the characteristics of the patients lost to follow-up been described? | | Partially | | Table 3 p.13 provides information on response rates by state for people with or without proxy at each step of |

| | | | | | |
|---|---|---|---|---|---|
| | | | | | the process: baseline and nine-month follow-up survey.<br><br>Also: '**We attempted nine month interviews with all sample members or their proxies, including those of deceased sample members and of consumers who disenrolled from Cash and Counseling (many of whom had returned to traditional agency-directed services).**' P.12. p14 and 15: brief discussion on disenrollment. |
| 10. | Have actual probability values been reported (e.g. 0.035 rather than <0.05) for the main outcomes except where the probability value is less than 0.001? | | yes | | Ex p.25 |
| | | | | | **Total reporting score: 9/11** |
| **External Validity** | | | | | |
| 11. | Were the subjects asked to participate in the study representative of the entire population from which they were recruited? | | Yes | | All eligible beneficiaries were invited to participate. Eligibility conditions differ across states but this is described in this review and results are reported by state separately |

| | | | | | |
|---|---|---|---|---|---|
| 12. | Were those subjects who were prepared to participate representative of the entire population from which they were recruited? | | Yes | | |
| 13. | Were the staff, places, and facilities where the patients were treated, representative of the treatment the majority of patients receive? | | Yes | | Differences between sites but results are also reported by sites |
| | | | | | **Total external validity score: 3/3** |
| **Internal Validity – Bias** | | | | | |
| 14. | Was an attempt made to blind study participants to the intervention they have received? | No | | | |
| 15. | Was an attempt made to blind those measuring outcomes of the intervention? | No | | | |
| 16. | If any of the results of the study were based on "data dredging", was this made clear? | | Yes | | |
| 17. | In trials and cohort studies, do the analyses adjust for different lengths of follow-up of patients, or in case control studies, is the time period between the intervention and outcomes the same for cases and controls? | | Yes | | year of enrollment is included as a control, see p.15 |
| 18. | Were the statistical tests used to assess the main outcomes appropriate? | | Yes | | **'We used binary logit models to obtain estimates of program impacts for categorical outcome measures. For continuous outcome measures (such as hours of care or Medicaid cost), we used ordinary least squares regression models.'** P.15 |
| 19. | Was compliance with the intervention/s reliable? | | Yes | | |

| | | | | | |
|---|---|---|---|---|---|
| 20. | Were the main outcome measures used accurate (valid and reliable)? | | Yes | | Subjective and objective measures used by other papers (Kunkel et al. 2002; Benjamin 2001; and Kane et al. 1994), p.14 |
| | | | | | **Total bias score: 5/7** |
| **Internal Validity – Confounding (Selection bias)** | | | | | |
| 21. | Were the patients in different intervention groups (trials and cohort studies) or were the cases and controls (case-control studies) recruited from the same population? | | Yes | | '**Interested consumers who met the eligibility criteria were given a baseline interview and then randomly assigned to the treatment group or the control group. Treatment group members had the option of disenrolling and returning to traditional agency-provided care any time they wished** '.p.12. from the same population within states |
| 22. | Were study subjects in different intervention groups (trial and cohort studies) or were the cases and controls (case-control studies) recruited over the same period of time? | | Yes | | See first category of Table 1, p.5 Differences across states but results are reported by states |
| 23. | Were study subjects randomised to intervention groups? | | Yes | | |
| 24. | Was the randomised intervention assignment concealed from both patients and health care staff until recruitment was compete and irrevocable? | | Yes | | '**Interested consumers who met the eligibility criteria were given a baseline interview and then randomly assigned to the treatment group or the control group**.' P12 |

| | | | | | |
|---|---|---|---|---|---|
| 25. | Was there adequate adjustment for confounding in the analyses from which the main findings were drawn? | | Yes | | **'controlled for the sample members' baseline measures of demographic characteristics, health and functioning, use of personal assistance, satisfaction with care and life, unmet needs, reasons for and year of enrollment, work and community activities, whether the sample member used a proxy respondent, and whether he or she appointed a representative. The analyses also controlled for baseline measures of several of the service use and quality outcomes used in this analysis**.' P.15 : various factors controlled for in regression models |
| 26. | Were losses of patients to follow-up taken into account? | | Yes | | |
| | | | | | **Total confounding score: 6/6** |
| **Power** | | | | | |
| 27. | *Did the study conduct a power analysis to determine the sample size needed to detect a significant difference in effect size for one or more outcome measures?* | | Yes | | See table A.4 in appendices p.D-14 |
| | | | | | **Total power score: 1/1** |
| | | | | | |
| | | | | | **Total quality score: 24/28** |

-----------------------------------------------------------------------

| Author (year): Foster et al., 2004<br>Study Title: DO CONSUMER-DIRECTED MEDICAID SUPPORTIVE SERVICES WORK FOR CHILDREN WITH DEVELOPMENTAL DISABILITIES?<br>Study Type: Randomised controlled trial | | | | | |
|---|---|---|---|---|---|
| | | Score | | | |
| | | 0 | 1 | 2 | Comment |
| **Reporting** | | | | | |
| 1. | Is the hypothesis/aim/objective of the study clearly described? | | Yes | | In executive summary and p.7 for hypotheses. |
| 2. | Are the main outcomes to be measured clearly described in the Introduction or Methods sections? | | Yes | | Yes, p.11 and 12, outcomes reported: total hours of assistance, paid and unpaid hours of assistance, measures based on scale, ex: satisfaction. See also table B.2 in appendix |
| 3. | Are the characteristics of the patients included in the study clearly described? | | Yes | | p.10, p16 (Table 1) and Table B.1, appendix p.A-4 |
| 4. | Are the interventions of interest clearly described? | | Yes | | C&C in Florida, with a focus on Children. From p.3 |
| 5. | Are the distributions of principal confounders in each group of subjects to be compared clearly described? | | Partially | | Significant differences between treatment and control groups at baseline are drawn, see Table B.1. in appendix. Also see table 1. Subgroup analysis is provided including the |

| | | | | | |
|---|---|---|---|---|---|
| | | | | | use of interaction terms but limited due to small sample sizes<br>p.14:' **we used regression models to control for the sample's baseline characteristics'** |
| 6. | Are the main findings of the study clearly described? | | Yes | | From p.17 and further. |
| 7. | Does the study provide estimates of the random variability in the data for the main outcomes? | No | | | No SE or CI |
| 8. | Have all important adverse events that may be a consequence of the intervention been reported? | | Yes | | Unmet needs or other outcomes included.<br>**'In past month, child: – Was injured while receiving paid help – Fell – Saw a doctor because of a fall – Saw a doctor because of cut, burn, or scale'**, see table B.2 |
| 9. | Have the characteristics of the patients lost to follow-up been described? | No | | | **'one parent of each child who enrolled in the demonstration (n = 1,002) completed a baseline interview, and then each child was randomly assigned to the treatment or control group.7 About nine months later, we attempted a follow-up interview with the same parents. (We interviewed a child's other parent, if necessary.) Nine-month interviews were completed by 859 parents, 441 (compared to 501 eligible) in the treatment group and 418 (compared to 501 eligible) in the control group, yielding response rates of 88 and 84 percent, respectively. To preserve the groups' comparability and obtain a complete** |

| | | | | | |
|---|---|---|---|---|---|
| | | | | | **picture of program experiences, we attempted to conduct nine-month interviews even if children were deceased or if those in the treatment group had disenrolled from CDC.**'<br><br>1002 id for baseline, 859 follow-up interviews.<br>In a footnote, '**Eleven percent of the eligible nonrespondents refused to be interviewed. The others could not be reached despite numerous attempts, at different times of day, over a one-month period. They also did not call MPR's toll-free telephone number to be interviewed at their convenience.**' reasons why there is a difference between eligible and interviewed people.<br><br>Also, Table B.3 describes samples used in the analysis, also mentionned deceased members.<br><br>However, nothing about characteristics. |
| 10. | Have actual probability values been reported (e.g. 0.035 rather than <0.05) for the main outcomes except where the probability value is less than 0.001? | | Yes | | Ex of table 3., p.23 |
| | | | | | **Total reporting score: 8/11** |
| **External Validity** | | | | | |

| | | | | | |
|---|---|---|---|---|---|
| 11. | Were the subjects asked to participate in the study representative of the entire population from which they were recruited? | No | | | Children with developmental disabilities in Florida between 3 and 17 |
| 12. | Were those subjects who were prepared to participate representative of the entire population from which they were recruited? | No | | | No information given |
| 13. | Were the staff, places, and facilities where the patients were treated, representative of the treatment the majority of patients receive? | No | | | Specificities in Florida |
| | | | | | **Total external validity score: 0/3** |
| **Internal Validity – Bias** | | | | | |
| 14. | Was an attempt made to blind study participants to the intervention they have received? | No | | | |
| 15. | Was an attempt made to blind those measuring outcomes of the intervention? | No | | | |
| 16. | If any of the results of the study were based on "data dredging", was this made clear? | | Yes | | No unplanned regression reported |
| 17. | In trials and cohort studies, do the analyses adjust for different lengths of follow-up of patients, or in case control studies, is the time period between the intervention and outcomes the same for cases and controls? | | Yes | | 9 months follow up interviews: same duration |
| 18. | Were the statistical tests used to assess the main outcomes appropriate? | | Yes | | OLS and Logit: 'For binary outcome measures, we assessed the impacts of CDC by using the estimated coefficients from logit ' and '. For the few continuous outcome measures |

| | | | | | |
|---|---|---|---|---|---|
| | | | | | **(hours of care received, of various types), we measured impacts by calculating the treatment-control difference in the means predicted by ordinary-least-squares regression models' p.14** |
| 19. | Was compliance with the intervention/s reliable? | | Yes | | |
| 20. | Were the main outcome measures used accurate (valid and reliable)? | | yes | | From other works, see Table b.2 in appendix, p.A-8 |
| | | | | | **Total bias score: 5/7** |
| **Internal Validity – Confounding (Selection bias)** | | | | | |
| 21. | Were the patients in different intervention groups (trials and cohort studies) or were the cases and controls (case-control studies) recruited from the same population? | | Yes | | |
| 22. | Were study subjects in different intervention groups (trial and cohort studies) or were the cases and controls (case-control studies) recruited over the same period of time? | | Yes | | Footnote in tables: '**MPR's baseline interview, conducted between June 2000 and August 2001, and nine-month evaluation interview, conducted between April 2001 and July 2002**.'. the assignement occurs after the baseline interview. |
| 23. | Were study subjects randomised to intervention groups? | | Yes | | |
| 24. | Was the randomised intervention assignment concealed from both patients and health care staff until recruitment was complete and irrevocable? | | Yes | | p.4: '**MPR conducted a baseline telephone interview with one parent of each enrolled child and then randomly assigned each child to the treatment or the control group**.' |
| 25. | Was there adequate adjustment for confounding in the analyses from which the main findings were drawn? | | Yes | | p.14:' **we used regression models to control for the sample's baseline characteristics'** but no more informations in |

| | | | | | |
|---|---|---|---|---|---|
| | | | | | footnote tables **and detailed results are not provided.** |
| 26. | Were losses of patients to follow-up taken into account? | | yes | | Discussed above, see question 9 |
| | | | | | **Total confounding score: 6/6** |
| **Power** | | | | | |
| 27. | *Did the study conduct a power analysis to determine the sample size needed to detect a significant difference in effect size for one or more outcome measures?* | | Yes | | p.14: statistical power analysis is conducted, see appendix table B.4. |
| | | | | | **Total power score: 1/1** |
| | | | | | |
| | | | | | **Total quality score: 20/28** |

------------------------------------------------------------------------

**Author (year): Glendinning et al., 2009**
**Study Title:** The Individual Budgets Pilot Projects: Impact and Outcomes for Carers
**Study Type:** randomised controlled trial

| | | Score | | | |
|---|---|---|---|---|---|
| | | 0 | 1 | 2 | Comment |
| **Reporting** | | | | | |
| 1. | Is the hypothesis/aim/objective of the study clearly described? | | Yes | | Impact of IBs on careers in section 1.5, p.9 |
| 2. | Are the main outcomes to be measured clearly described in the Introduction or Methods sections? | | Yes | | Section 2.3.3, description of outcomes used for careers. Outcomes already used in other (published) work |

| | | | | | |
|---|---|---|---|---|---|
| 3. | Are the characteristics of the patients included in the study clearly described? | | Yes | | Section 2.4.2 on demographic and household characteristics. Also see tables 2.3 and 2.4. |
| 4. | Are the interventions of interest clearly described? | | Yes | | IBs are clearly described in the first chapter of the report, from p.1 and further |
| 5. | Are the distributions of principal confounders in each group of subjects to be compared clearly described? | | Partially | | No confounder mentioned.<br>Table 2.3 provides differences btw the two groups, '**Within the structured interview carer sample, there were no significant differences between the IB and comparison groups on each of four demographic variables.**' P.10 combined with table 2.4.<br><br>Adding controls in regression models:' **We used statistical models to explore the implications of receipt of an IB and to explore other potential influences on outcomes. Potential influences included measures of baseline needs; carer and service user characteristics; circumstances (such as age, gender and whether the carer was living with the service user); and** |

| | | | | | |
|---|---|---|---|---|---|
| | | | | | **operational measures such as whether or not an IB holder had their support plan in place at the time of the structured interview with the carer.'** |
| 6. | Are the main findings of the study clearly described? | | Yes | | Chapters 4, 5 and 6 |
| 7. | Does the study provide estimates of the random variability in the data for the main outcomes? | | Yes | | SD are reported |
| 8. | Have all important adverse events that may be a consequence of the intervention been reported? | | partially | | The adverse effect of RAS has been reported |
| 9. | Have the characteristics of the patients lost to follow-up been described? | No | | | |
| 10. | Have actual probability values been reported (e.g. 0.035 rather than <0.05) for the main outcomes except where the probability value is less than 0.001? | | Yes | | In section 6 |
| | | | | | **Total reporting score: 9/11** |
| **External Validity** | | | | | |
| 11. | Were the subjects asked to participate in the study representative of the entire population from which they were recruited? | Unable | | | Different sites targeted and finally different population targeted (even if at the beginning the authors wanted to focus on specific relationship btw careers and care recipient, they had to change due to small ample sizes) |

| | | | | | |
|---|---|---|---|---|---|
| 12. | Were those subjects who were prepared to participate representative of the entire population from which they were recruited? | Unable | | | |
| 13. | Were the staff, places, and facilities where the patients were treated, representative of the treatment the majority of patients receive? | | Yes | | |
| | | | | | **Total external validity score: 1/3** |
| **Internal Validity – Bias** | | | | | |
| 14. | Was an attempt made to blind study participants to the intervention they have received? | No | | | |
| 15. | Was an attempt made to blind those measuring outcomes of the intervention? | No | | | |
| 16. | If any of the results of the study were based on "data dredging", was this made clear? | | Yes | | |
| 17. | In trials and cohort studies, do the analyses adjust for different lengths of follow-up of patients, or in case control studies, is the time period between the intervention and outcomes the same for cases and controls? | No | | | Interview with users: 6 months after registration (Glendinning, 2008)<br>Interview with careers :between one and ten months after interview with service users so not the same period of time for all careers. |
| 18. | Were the statistical tests used to assess the main outcomes appropriate? | | Yes | | Linear multivariate analysis is mentioned but not more detail on estimators |
| 19. | Was compliance with the intervention/s reliable? | | Yes | | |
| 20. | Were the main outcome measures used accurate (valid and reliable)? | | yes | | Also used in previous (published work). See section 2.3.3 for references |

| | | | | | |
|---|---|---|---|---|---|
| | | | | | **Total bias score: 4/7** |
| **Internal Validity – Confounding (Selection bias)** | | | | | |
| 21. | Were the patients in different intervention groups (trials and cohort studies) or were the cases and controls (case-control studies) recruited from the same population? | | yes | | |
| 22. | Were study subjects in different intervention groups (trial and cohort studies) or were the cases and controls (case-control studies) recruited over the same period of time? | | yes | | See table 2.1 |
| 23. | Were study subjects randomised to intervention groups? | | Yes | | |
| 24. | Was the randomised intervention assignment concealed from both patients and health care staff until recruitment was compete and irrevocable? | | Yes | | **'At the heart of the main IBSEN evaluation was a randomised controlled trial. Those eligible for the study (new social care referrals and/or existing service users undergoing review) were identified by IB pilot sites and registered with the IBSEN website; at this point the presence (or otherwise) of a carer was also recorded. Registered people were then randomised into two groups:'** p.11 |

| | | | | | |
|---|---|---|---|---|---|
| 25. | Was there adequate adjustment for confounding in the analyses from which the main findings were drawn? | | Yes | | controlled for in analysis, e.g. baseline characteristics, baseline needs |
| 26. | Were losses of patients to follow-up taken into account? | | Yes | | **'Problems in tracking down the carers of the original IBSEN study participants had an impact on sample sizes, which were smaller than had been planned. It also resulted in a more diverse sample of carers than originally intended'**. Only people with baseline and follow-up data were included |
| | | | | | **Total confounding score: 6/6** |
| **Power** | | | | | |
| 27. | *Did the study conduct a power analysis to determine the sample size needed to detect a significant difference in effect size for one or more outcome measures?* | No | | | |
| | | | | | **Total power score: 0/1** |
| | | | | | |
| | | | | | **Total quality score: 20/28** |

| **Author (year): Shen et al., 2008**<br>**Study Title:** Consumer-Directed Care for Beneficiaries With Mental Illness: Lessons From New Jersey's Cash and Counseling Program<br>**Study Type:** | | | | | |
|---|---|---|---|---|---|
| | | Score | | | |
| | | 0 | 1 | 2 | Comment |
| **Reporting** | | | | | |
| 1. | Is the hypothesis/aim/objective of the study clearly described? | | Yes | | In abstract and introduction parts |

| | | | | | |
|---|---|---|---|---|---|
| 2. | Are the main outcomes to be measured clearly described in the Introduction or Methods sections? | | Yes | | Subsection dedicated to the description of the outcomes |
| 3. | Are the characteristics of the patients included in the study clearly described? | | Yes | | See table 1 and the section about 'major characteristics of the sample' |
| 4. | Are the interventions of interest clearly described? | | Yes | | Description of C&C |
| 5. | Are the distributions of principal confounders in each group of subjects to be compared clearly described? | | partially | | Particpants were compared at baseline and statistical difference was shown for gender. Sociodemographic characteristics are controlled for in regressions. See table 1 |
| 6. | Are the main findings of the study clearly described? | | Yes | | |
| 7. | Does the study provide estimates of the random variability in the data for the main outcomes? | No | | | No SD or CI |
| 8. | Have all important adverse events that may be a consequence of the intervention been reported? | | Yes | | Yes, see abstract and p.1301, section 'chi square analysis'. Some outcomes used are related to adverse events that may occur with consumer-directed care |
| 9. | Have the characteristics of the patients lost to follow-up been described? | No | | | |
| 10. | Have actual probability values been reported (e.g. 0.035 rather than <0.05) for the main | No | | | Three levels of sig, 0.01; 0.05; 0.1 |

| | | | | | |
|---|---|---|---|---|---|
| | outcomes except where the probability value is less than 0.001? | | | | |
| **Total reporting score: 7/11** | | | | | |
| **External Validity** | | | | | |
| 11. | Were the subjects asked to participate in the study representative of the entire population from which they were recruited? | No | | | Focus on people with mental disabilities, nonelderly, not representative from C&C and Medicaid |
| 12. | Were those subjects who were prepared to participate representative of the entire population from which they were recruited? | No | | | |
| 13. | Were the staff, places, and facilities where the patients were treated, representative of the treatment the majority of patients receive? | | Yes | | |
| **Total external validity score: 1/3** | | | | | |
| **Internal Validity – Bias** | | | | | |
| 14. | Was an attempt made to blind study participants to the intervention they have received? | No | | | |
| 15. | Was an attempt made to blind those measuring outcomes of the intervention? | No | | | |
| 16. | If any of the results of the study were based on "data dredging", was this made clear? | | Yes | | No unplanned analysis or subgroup analysis reported |
| 17. | In trials and cohort studies, do the analyses adjust for different lengths of follow-up of patients, or in case control studies, is the time period between the intervention and outcomes the same for cases and controls? | | Yes | | 9 months follow-up for interviews |

| | | | | | |
|---|---|---|---|---|---|
| 18. | Were the statistical tests used to assess the main outcomes appropriate? | | Yes | | Multivariate logistic regression models |
| 19. | Was compliance with the intervention/s reliable? | | Yes | | |
| 20. | Were the main outcome measures used accurate (valid and reliable)? | | Yes | | Refers to established measures from other work |
| | | | | | **Total bias score: 5/7** |
| **Internal Validity – Confounding (Selection bias)** | | | | | |
| 21. | Were the patients in different intervention groups (trials and cohort studies) or were the cases and controls (case-control studies) recruited from the same population? | | Yes | | C&C participants for treatment group and participants who receive services provided by an agency for control group, based on Medicaid claim data |
| 22. | Were study subjects in different intervention groups (trial and cohort studies) or were the cases and controls (case-control studies) recruited over the same period of time? | | Yes | | Enrolment period for C&C from 1999 to 2002 so at different times |
| 23. | Were study subjects randomised to intervention groups? | | Yes | | |
| 24. | Was the randomised intervention assignment concealed from both patients and health care staff until recruitment was compete and irrevocable? | Unable to determine | | | |
| 25. | Was there adequate adjustment for confounding in the analyses from which the main findings were drawn? | | yes | | Relevant covariates were controlled for in regression analysis |
| 26. | Were losses of patients to follow-up taken into account? | | Yes | | Only people with baseline and follow-up data were included in the |

| | | | | | |
|---|---|---|---|---|---|
| | | | | | final evaluation sample but no more details |
| | | | | | **Total confounding score: 4/6** |
| **Power** | | | | | |
| 27. | *Did the study conduct a power analysis to determine the sample size needed to detect a significant difference in effect size for one or more outcome measures?* | No | | | |
| | | | | | **Total power score: 0/1** |
| | | | | | |
| | | | | | **Total quality score: 17/28** |

---------------------------------------------------------------------------

| **Author (year): Foster et al., 2003**<br>**Study Title:** EASING THE BURDEN OF CAREGIVING: THE IMPACT OF CONSUMER DIRECTION ON PRIMARY INFORMAL CAREGIVERS IN ARKANSAS<br>**Study Type:** RCT | | | | | |
|---|---|---|---|---|---|
| | | Score | | | |
| | | 0 | 1 | 2 | Comment |
| **Reporting** | | | | | |
| 1. | Is the hypothesis/aim/objective of the study clearly described? | | Yes | | See Executive summary. P.ii |
| 2. | Are the main outcomes to be measured clearly described in the Introduction or Methods sections? | | Yes | | See Executive summary. P.ii and p.6. More details are provided in Table A.1. |

| | | | | | |
|---|---|---|---|---|---|
| 3. | Are the characteristics of the patients included in the study clearly described? | | Yes | | Table 1: characteristics of baseline care recipient and Table 2 about the informal caregivers (age, race, marital status etc.) |
| 4. | Are the interventions of interest clearly described? | | Yes | | In The introduction and Background part |
| 5. | Are the distributions of principal confounders in each group of subjects to be compared clearly described? | No | | | Baseline characteristics differences provided and baseline CG/CR characteristics included as controls in regression models but no mention of confounding factors. |
| 6. | Are the main findings of the study clearly described? | | Yes | | |
| 7. | Does the study provide estimates of the random variability in the data for the main outcomes? | No | | | No SE or CI reported |
| 8. | Have all important adverse events that may be a consequence of the intervention been reported? | | Partially | | Some adverse events are mentioned, even discussed but results are not shown. P13 for an example |
| 9. | Have the characteristics of the patients lost to follow-up been described? | No | | | Number of disenrolled people is mentioned but their characteristics are not reported |
| 10. | Have actual probability values been reported (e.g. 0.035 rather than <0.05) for the main outcomes except where the probability value is less than 0.001? | | Yes | | |
| | | | | | **Total reporting score: 7/11** |
| **External Validity** | | | | | |

| | | | | | |
|---|---|---|---|---|---|
| 11. | Were the subjects asked to participate in the study representative of the entire population from which they were recruited? | No | | | Selection procedure is explained: care recipients participating in C&C were asked to name a CG but not all have informal caregivers. |
| 12. | Were those subjects who were prepared to participate representative of the entire population from which they were recruited? | | Yes | | The proportion of those asked who agreed to participate is clearly stated 82% of eligible in the C group and 84% of eligible in the T group |
| 13. | Were the staff, places, and facilities where the patients were treated, representative of the treatment the majority of patients receive? | No | | | Specificities in Arkansas |
| | | | | | **Total external validity score: 1/3** |
| **Internal Validity – Bias** | | | | | |
| 14. | Was an attempt made to blind study participants to the intervention they have received? | No | | | |
| 15. | Was an attempt made to blind those measuring outcomes of the intervention? | No | | | |
| 16. | If any of the results of the study were based on "data dredging", was this made clear? | | Yes | | |
| 17. | In trials and cohort studies, do the analyses adjust for different lengths of follow-up of patients, or in case control studies, is the time period between the intervention and outcomes the same for cases and controls? | | Yes | | Interviews and questionnaire were carried out at fixed intervals after enrolment: interviews were carried out 9 months after enrollment for care recipients and 10 for CGs |
| 18. | Were the statistical tests used to assess the main outcomes appropriate? | | Yes | | OLS, logit, ordered logit |
| 19. | Was compliance with the intervention/s reliable? | | Yes | | |

| | | | | | |
|---|---|---|---|---|---|
| 20. | Were the main outcome measures used accurate (valid and reliable)? | | Yes | | Derived from the survey |
| | | | | | **Total bias score: 5/7** |
| **Internal Validity – Confounding (Selection bias)** | | | | | |
| 21. | Were the patients in different intervention groups (trials and cohort studies) or were the cases and controls (case-control studies) recruited from the same population? | | Yes | | |
| 22. | Were study subjects in different intervention groups (trial and cohort studies) or were the cases and controls (case-control studies) recruited over the same period of time? | | Yes | | Interviewed between December 1998 and April 2001 |
| 23. | Were study subjects randomised to intervention groups? | | Yes | | |
| 24. | Was the randomised intervention assignment concealed from both patients and health care staff until recruitment was compete and irrevocable? | | Yes | | Participants completed the interview before being assigned to a group |
| 25. | Was there adequate adjustment for confounding in the analyses from which the main findings were drawn? | | Yes | | CR and CG baseline characteristics are included in regression models |
| 26. | Were losses of patients to follow-up taken into account? | | Yes | | '**to preserve the comparability of the two groups of caregivers and obtain a complete picture of their experiences, we conducted interviews with caregivers even if their care recipients were deceased or, in treatment group cases, disenrolled from IndependentChoices.**'p.6. |

| | | | | | |
|---|---|---|---|---|---|
| | | | | | Response rates are reported. |
| | | | | | **Total confounding score: 6/6** |
| **Power** | | | | | |
| 27. | *Did the study conduct a power analysis to determine the sample size needed to detect a significant difference in effect size for one or more outcome measures?* | | Yes | | A statistical power analysis is reported, p.29 |
| | | | | | **Total power score: 1/1** |
| | | | | | |
| | | | | | **Total quality score: 20/28** |

| | | | | | |
|---|---|---|---|---|---|
| **Author (year): P**rabhakar, Thom and Johnson (2011)<br>**Study Title:** Individual budgets for families with disabled children Final evaluation report: The IB process<br>**Study Type:** Uncontrolled before after study | | | | | |
| | | Score | | | |
| | | 0 | 1 | 2 | Comment |
| **Reporting** | | | | | |
| 1. | Is the hypothesis/aim/objective of the study clearly described? | | Yes | | Objectives of the programme outlined in the Introduction: The IB Programme |
| 2. | Are the main outcomes to be measured clearly described in the Introduction or Methods sections? | No | | | The Programme allows an initial discussion held with family to identify family defined outcomes. The outcomes are explained only in the Appendix, p.B-6. This is the fist volume out of three. |

| | | | | | |
|---|---|---|---|---|---|
| 3. | Are the characteristics of the patients included in the study clearly described? | No | | | This paper only described approaches adopted to implement the pilot and the lessons emerging, there is no method section, no quantitative analysis and no quantitative results presented. |
| 4. | Are the interventions of interest clearly described? | | Yes | | Main goal of this article. |
| 5. | Are the distributions of principal confounders in each group of subjects to be compared clearly described? | No | | | |
| 6. | Are the main findings of the study clearly described? | | Partially | | Summary findings are presented from Table 7, p. 24 and further. **'The Recommendations and Implications, which draws together the findings of the evaluation and presents recommendations for the future use of the IB approach'** are presented in the third volume of the report. |
| 7. | Does the study provide estimates of the random variability in the data for the main outcomes? | No | | | |
| 8. | Have all important adverse events that may be a consequence of the intervention been reported? | No | | | No adverse effects reported. |
| 9. | Have the characteristics of the patients lost to follow-up been described? | No | | | |

| | | | | | |
|---|---|---|---|---|---|
| 10. | Have actual probability values been reported (e.g. 0.035 rather than <0.05) for the main outcomes except where the probability value is less than 0.001? | No | | | |
| | | | | | **Total reporting score: 3/11** |
| **External Validity** | | | | | |
| 11. | Were the subjects asked to participate in the study representative of the entire population from which they were recruited? | No | | | p.40: '**the invitation to participate in the pilot was therefore facilitated in one of two ways: the first invited a random sample of the population to participate; and the second invited a targeted set of eligible families with disabled children to participate**.', some were targeted some were randomly selected. |
| 12. | Were those subjects who were prepared to participate representative of the entire population from which they were recruited? | No | | | |
| 13. | Were the staff, places, and facilities where the patients were treated, representative of the treatment the majority of patients receive? | | Yes | | |
| | | | | | **Total external validity score: 1/3** |
| **Internal Validity – Bias** | | | | | |
| 14. | Was an attempt made to blind study participants to the intervention they have received? | No | | | |

| | | | | | |
|---|---|---|---|---|---|
| 15. | Was an attempt made to blind those measuring outcomes of the intervention? | No | | | |
| 16. | If any of the results of the study were based on "data dredging", was this made clear? | | Yes | | |
| 17. | In trials and cohort studies, do the analyses adjust for different lengths of follow-up of patients, or in case control studies, is the time period between the intervention and outcomes the same for cases and controls? | No | | | This is a 2 year programme but no information provided on different lengths of follow-up of patients. Figure 15 sets out the engagement profile by site for the programme. |
| 18. | Were the statistical tests used to assess the main outcomes appropriate? | Unable to determine | | | Significant results are briefly summarize. |
| 19. | Was compliance with the intervention/s reliable? | | Yes | | |
| 20. | Were the main outcome measures used accurate (valid and reliable)? | No | | | Outcome measures used are unclear. |
| | | | | | **Total bias score: 2/7** |
| **Internal Validity – Confounding (Selection bias)** | | | | | |
| 21. | Were the patients in different intervention groups (trials and cohort studies) or were the cases and controls (case-control studies) recruited from the same population? | | Yes | | Yes, recruited from the same population within pilot sites. |
| 22. | Were study subjects in different intervention groups (trial and cohort studies) or were the cases and controls (case-control studies) recruited over the same period of time? | | Yes | | |
| 23. | Were study subjects randomised to intervention groups? | No | | | Each site uses a random sample approach for half of the participants. |

| | | | | | |
|---|---|---|---|---|---|
| 24. | Was the randomised intervention assignment concealed from both patients and health care staff until recruitment was compete and irrevocable? | No | | | |
| 25. | Was there adequate adjustment for confounding in the analyses from which the main findings were drawn? | No | | | No mention of confounding. |
| 26. | Were losses of patients to follow-up taken into account? | No | | | |
| | | | | | **Total confounding score:2 /6** |
| **Power** | | | | | |
| 27. | *Did the study conduct a power analysis to determine the sample size needed to detect a significant difference in effect size for one or more outcome measures?* | No | | | |
| | | | | | **Total power score: 0/1** |
| | | | | | |
| | | | | | **Total quality score: 8/28** |

**Author (year):** Prabhakar and Thom (2012)
**Study Title:** Evaluation of the extended individual budget pilot programme for families with disabled children: the extended packages
**Study Type:** Uncontrolled before after study

| | | | | | |
|---|---|---|---|---|---|
| | | Score | | | |
| | | 0 | 1 | 2 | Comment |
| **Reporting** | | | | | |

| | | | | | |
|---|---|---|---|---|---|
| 1. | Is the hypothesis/aim/objective of the study clearly described? | | Yes | | The IB programme was extended for one more year. This report is one of the two volumes reporting the findings from the extended year. '**Purpose of the report'**, p.1 |
| 2. | Are the main outcomes to be measured clearly described in the Introduction or Methods sections? | No | | | Not clearly stated, health outcomes, and educational child outcomes are mentioned but it appears that no clear definitions are given in the main text. In a citation p.40, it is 'guessable' that quality of life is include in the outcome list. |
| 3. | Are the characteristics of the patients included in the study clearly described? | No | | | |
| 4. | Are the interventions of interest clearly described? | | Yes | | 'Intentions of the extended pilot', p.iv |
| 5. | Are the distributions of principal confounders in each group of subjects to be compared clearly described? | No | | | No confounding factors, omitted variables, heterogeneity mentioned. |
| 6. | Are the main findings of the study clearly described? | | Partially | | Chapter 6 sets out the main evaluation findings, p.53 |
| 7. | Does the study provide estimates of the random variability in the data for the main outcomes? | No | | | |
| 8. | Have all important adverse events that may be a consequence of the intervention been reported? | No | | | |
| 9. | Have the characteristics of the patients lost to follow-up been described? | No | | | |

| | | | | | |
|---|---|---|---|---|---|
| 10. | Have actual probability values been reported (e.g. 0.035 rather than <0.05) for the main outcomes except where the probability value is less than 0.001? | No | | | Significant results are mentioned but no p-value reported |
| | | | | | **Total reporting score: 3/11** |
| **External Validity** | | | | | |
| 11. | Were the subjects asked to participate in the study representative of the entire population from which they were recruited? | No | | | **'All six pilots intended to recruit additional families to take part in the extended year of the pilot. The rationale for recruitment varied: from testing out the use of IBs with a different cohort (for instance a younger age group); to simply rolling (social care) IBs out further to a larger number; to targeting those that currently receive specific health or education funding that was in the scope of the pilot'** p.14 added to the same population than the original 2 year IB programme: some were targeted some were randomly selected. |
| 12. | Were those subjects who were prepared to participate representative of the entire population from which they were recruited? | No | | | |

| | | | | | |
|---|---|---|---|---|---|
| 13. | Were the staff, places, and facilities where the patients were treated, representative of the treatment the majority of patients receive? | | Yes | | |
| **Total external validity score: 1/3** | | | | | |
| **Internal Validity – Bias** | | | | | |
| 14. | Was an attempt made to blind study participants to the intervention they have received? | No | | | |
| 15. | Was an attempt made to blind those measuring outcomes of the intervention? | No | | | |
| 16. | If any of the results of the study were based on "data dredging", was this made clear? | | Yes | | |
| 17. | In trials and cohort studies, do the analyses adjust for different lengths of follow-up of patients, or in case control studies, is the time period between the intervention and outcomes the same for cases and controls? | Unable to determine | | | One more year: extended IB programme but differences between sites: '**It was apparent from the plans that the date (of review) varied by funding stream, by the speed at which families could be recruited to the pilot, and/or by the time it was anticipated it would take to develop and take families through the process (identification of budgets, support plans developed/approved and support commissioned**)',p.14 |
| 18. | Were the statistical tests used to assess the main outcomes appropriate? | No | | | No statistical test reported |
| 19. | Was compliance with the intervention/s reliable? | | Yes | | |

| | | | | | |
|---|---|---|---|---|---|
| 20. | Were the main outcome measures used accurate (valid and reliable)? | No | | | |
| | | | | | **Total bias score: 2/7** |
| **Internal Validity – Confounding (Selection bias)** | | | | | |
| 21. | Were the patients in different intervention groups (trials and cohort studies) or were the cases and controls (case-control studies) recruited from the same population? | | Yes | | |
| 22. | Were study subjects in different intervention groups (trial and cohort studies) or were the cases and controls (case-control studies) recruited over the same period of time? | | Yes | | Differences across sites however. |
| 23. | Were study subjects randomised to intervention groups? | No | | | |
| 24. | Was the randomised intervention assignment concealed from both patients and health care staff until recruitment was compete and irrevocable? | No | | | |
| 25. | Was there adequate adjustment for confounding in the analyses from which the main findings were drawn? | No | | | Confounding factor or omitted variables not mentioned. |
| 26. | Were losses of patients to follow-up taken into account? | No | | | |
| | | | | | **Total confounding score: 2/6** |
| **Power** | | | | | |
| 27. | *Did the study conduct a power analysis to determine the sample size needed to detect a significant* | No | | | |

| | | | | | |
|---|---|---|---|---|---|
| | *difference in effect size for one or more outcome measures?* | | | | |
| **Total power score: 0/1** | | | | | |
| | | | | | |
| **Total quality score: 8/28** | | | | | |

| **Author (year):** Thom and Prabhakar (2011)<br>**Study Title:** Individual budgets for families with disabled children Final evaluation report: Recommendations and implications<br>**Study Type:** Uncontrolled before after study | | | | | |
|---|---|---|---|---|---|
| | | Score | | | |
| | | 0 | 1 | 2 | Comment |
| **Reporting** | | | | | |
| 1. | Is the hypothesis/aim/objective of the study clearly described? | | Yes | | ‘**This report is one of three volumes containing the findings from the first two years of the Individual Budgets for disabled children pilot programme.**’p.1. Objectives of the programme is provided p.3 |
| 2. | Are the main outcomes to be measured clearly described in the Introduction or Methods sections? | | Partially | | See table 6 p.19 that reports change in outcomes for family. Also see Table 10, p.22 about change in impacts for families. A subsection p.28 details the impact of IBs on families |
| 3. | Are the characteristics of the patients included in the study clearly described? | No | | | |

| | | | | | |
|---|---|---|---|---|---|
| 4. | Are the interventions of interest clearly described? | | Yes | | Description of IB programme From 1.2 on p.1 and further |
| 5. | Are the distributions of principal confounders in each group of subjects to be compared clearly described? | No | | | No confounders reported |
| 6. | Are the main findings of the study clearly described? | | Yes | | Aim of this volume. See 4.10, p.28 for an example |
| 7. | Does the study provide estimates of the random variability in the data for the main outcomes? | No | | | |
| 8. | Have all important adverse events that may be a consequence of the intervention been reported? | No | | | No adverse even reported |
| 9. | Have the characteristics of the patients lost to follow-up been described? | No | | | |
| 10. | Have actual probability values been reported (e.g. 0.035 rather than <0.05) for the main outcomes except where the probability value is less than 0.001? | No | | | Significant results are mentioned but no p-value reported |
| | | | | | **Total reporting score: 4/11** |
| **External Validity** | | | | | |
| 11. | Were the subjects asked to participate in the study representative of the entire population from which they were recruited? | No | | | Population from the original IB programme: some were targeted some were randomly selected |
| 12. | Were those subjects who were prepared to participate representative of the entire population from which they were recruited? | No | | | |

| | | | | | |
|---|---|---|---|---|---|
| 13. | Were the staff, places, and facilities where the patients were treated, representative of the treatment the majority of patients receive? | | Yes | | |
| | | | | | **Total external validity score: 1/3** |
| **Internal Validity – Bias** | | | | | |
| 14. | Was an attempt made to blind study participants to the intervention they have received? | No | | | |
| 15. | Was an attempt made to blind those measuring outcomes of the intervention? | No | | | |
| 16. | If any of the results of the study were based on "data dredging", was this made clear? | | Yes | | |
| 17. | In trials and cohort studies, do the analyses adjust for different lengths of follow-up of patients, or in case control studies, is the time period between the intervention and outcomes the same for cases and controls? | Unable to determine | | | |
| 18. | Were the statistical tests used to assess the main outcomes appropriate? | Unable to determine | | | No information on statistical analysis provided. |
| 19. | Was compliance with the intervention/s reliable? | | Yes | | |
| 20. | Were the main outcome measures used accurate (valid and reliable)? | No | | | No formally tools used, no information provided. |
| | | | | | **Total bias score: 2/7** |
| **Internal Validity – Confounding (Selection bias)** | | | | | |
| 21. | Were the patients in different intervention groups (trials and cohort studies) or were the cases and controls (case-control studies) recruited from the same population? | | Yes | | |

| | | | | | |
|---|---|---|---|---|---|
| 22. | Were study subjects in different intervention groups (trial and cohort studies) or were the cases and controls (case-control studies) recruited over the same period of time? | | Yes | | |
| 23. | Were study subjects randomised to intervention groups? | No | | | |
| 24. | Was the randomised intervention assignment concealed from both patients and health care staff until recruitment was compete and irrevocable? | No | | | |
| 25. | Was there adequate adjustment for confounding in the analyses from which the main findings were drawn? | No | | | No confounding bias mentioned, potential confounders are not listed or included |
| 26. | Were losses of patients to follow-up taken into account? | No | | | |
| | | | | | **Total confounding score: 2/6** |
| **Power** | | | | | |
| 27. | *Did the study conduct a power analysis to determine the sample size needed to detect a significant difference in effect size for one or more outcome measures?* | No | | | |
| | | | | | **Total power score: 0/1** |
| | | | | | |
| | | | | | **Total quality score: 9/28** |

| |
|---|
| **Author (year):** Johnson, Graham and Prabhakar (2011) |

| **Study Title:** Individual budgets for families with disabled children Final evaluation report: The family journey<br>**Study Type:** Uncontrolled before after study | | | | | |
|---|---|---|---|---|---|
| | | Score | | | |
| | | 0 | 1 | 2 | Comment |
| **Reporting** | | | | | |
| 1. | Is the hypothesis/aim/objective of the study clearly described? | | Yes | | This is the second volume out of three about '**The Family Journey evaluation, which provides an assessment of the outcomes and distance travelled by participating families'** p.1 |
| 2. | Are the main outcomes to be measured clearly described in the Introduction or Methods sections? | | Yes | | Section 6 is about the outcomes achieved by participating families from p.37 and further. |
| 3. | Are the characteristics of the patients included in the study clearly described? | | Yes | | From p.23, a section entitled 'Nature of participants' described the characteristics of participating families and their children. |
| 4. | Are the interventions of interest clearly described? | | Yes | | Section 2 is an an introduction to the IB Approach and the pilot sites. |
| 5. | Are the distributions of principal confounders in each group of subjects to be compared clearly described? | No | | | No confounders listed or described |
| 6. | Are the main findings of the study clearly described? | | Yes | | For a summary, see Table 35, p.54. |
| 7. | Does the study provide estimates of the random variability in the data for the main outcomes? | No | | | |

| | | | | | |
|---|---|---|---|---|---|
| 8. | Have all important adverse events that may be a consequence of the intervention been reported? | No | | | |
| 9. | Have the characteristics of the patients lost to follow-up been described? | No | | | **‘33 of the families had left the pilot since the baseline**.’ P.30. Section 5 provided information on why these families had left the programme but their characteristics are not clearly described. |
| 10. | Have actual probability values been reported (e.g. 0.035 rather than <0.05) for the main outcomes except where the probability value is less than 0.001? | No | | | Significant results mentioned but no p-value reported. |
| | | | | | **Total reporting score: 5/11** |
| **External Validity** | | | | | |
| 11. | Were the subjects asked to participate in the study representative of the entire population from which they were recruited? | No | | | |
| 12. | Were those subjects who were prepared to participate representative of the entire population from which they were recruited? | No | | | |
| 13. | Were the staff, places, and facilities where the patients were treated, representative of the treatment the majority of patients receive? | | Yes | | |
| | | | | | **Total external validity score: 1/3** |
| **Internal Validity – Bias** | | | | | |
| 14. | Was an attempt made to blind study participants to the intervention they have received? | No | | | |

| | | | | | |
|---|---|---|---|---|---|
| 15. | Was an attempt made to blind those measuring outcomes of the intervention? | No | | | |
| 16. | If any of the results of the study were based on "data dredging", was this made clear? | | Yes | | |
| 17. | In trials and cohort studies, do the analyses adjust for different lengths of follow-up of patients, or in case control studies, is the time period between the intervention and outcomes the same for cases and controls? | | Yes | | Time period is supposed to be the same for all participating families: '**Family baseline and follow up surveys – families were interviewed as close to the point of recruitment on to the pilot as possible to capture their baseline position and subsequently were interviewed again as close to the end of the 71: Introduction 8 pilot activity as possible to enable the evaluation to measure distance travelled from the baseline position**.' P.7. |
| 18. | Were the statistical tests used to assess the main outcomes appropriate? | No | | | |
| 19. | Was compliance with the intervention/s reliable? | | Yes | | |
| 20. | Were the main outcome measures used accurate (valid and reliable)? | | partially | | It appears to be consistent with other programme evaluation studies, and table notes included '**Content that relates to outcomes is greyed out and was discussed in the previous chapter'.** |

| | | | | | |
|---|---|---|---|---|---|
| | | | | | **Total bias score: 3/7** |
| **Internal Validity – Confounding (Selection bias)** | | | | | |
| 21. | Were the patients in different intervention groups (trials and cohort studies) or were the cases and controls (case-control studies) recruited from the same population? | | Yes | | |
| 22. | Were study subjects in different intervention groups (trial and cohort studies) or were the cases and controls (case-control studies) recruited over the same period of time? | | Yes | | |
| 23. | Were study subjects randomised to intervention groups? | No | | | |
| 24. | Was the randomised intervention assignment concealed from both patients and health care staff until recruitment was compete and irrevocable? | No | | | |
| 25. | Was there adequate adjustment for confounding in the analyses from which the main findings were drawn? | No | | | No confounders mentioned or included in the analyses. |
| 26. | Were losses of patients to follow-up taken into account? | | Yes | | Section 5 is dedicated to families who had left the pilot, see p.30. They are excluded from the analyses. |
| | | | | | **Total confounding score: 3/6** |
| **Power** | | | | | |
| 27. | *Did the study conduct a power analysis to determine the sample size needed to detect a significant* | No | | | |

| | | | | | |
|---|---|---|---|---|---|
| | *difference in effect size for one or more outcome measures?* | | | | |
| **Total power score: 0/1** | | | | | |
| | | | | | |
| **Total quality score: 12/28** | | | | | |

Table S10. Article characteristics

| | **Article** | **Design** | **Location** | **Disability** | **Sample** | **Treatment group** | **Control or Comparison group** | **Outcomes** |
|---|---|---|---|---|---|---|---|---|
| | *Author (year)* | *Description of study design* | *Country or region* | *Nature of disabilities* | *Number of participants (total/treatment group/control group)*<br><br>*Male/Female breakdown*<br><br>*Age* | *Terminology used*<br><br>*Description of personal budget program*<br><br>*Duration of program or care* | *Description of comparison group, (if applicable)* | *List of Outcomes relevant for this review* |
| 1. | Beatty et al. (1998) | Quasi-experimental longitudinal comparative survey<br><br>(Controlled cross-sectional survey) | Virginia, U.S.A. | Physical disabilities | 92 participants Treatment:60, Control: 32<br><br>Female: Treatment 47%, control 42%. | Consumer directed personal assistance service.<br><br>Service users could hire, fire and train their attendants. Users participated in deciding the number of hours | On the waiting list for consumer-directed PAS | • Satisfaction with services |

| | | | | | | | | |
|---|---|---|---|---|---|---|---|---|
| | | | | | Average age: treatment 41.7, control 43.7 | and type of service required. | | |
| 2. | Benjamin et al. (2000) | Controlled cross-sectional survey | California, U.S.A. | Mixed disabilities, but people with severe cognitive impairment were excluded from the sampling frame. | Participants 1095. Treatment: 511, control 584.<br><br>Female: Treatment 69.9%, control 76.8%.<br><br>% 65 and over: Treatment 53.6%, control 50%. | Consumer directed in-home supportive services.<br><br>Service users could hire anyone they chose, even family, to provide services. | Receiving care under the professional home-care agency model. | • Unmet needs<br>• Service satisfaction |
| 3. | Brown et al. (2007) | Randomised controlled trial | 3 sites in U.S.A. Arkansas, Florida, New Jersey | Various<br><br>Arkansas: physical disabilities (may also have cognitive disabilities)<br><br>New Jersey: Physical disabilities (and perhaps cognitive disabilities) | Total participants (adults + children): 2825 (1966 + 859). Adults – treatment = 1007, Adults – control = 959. Children – treatment = 441, Children – control = 418. Arkansas (T) 243, (C) 230, | Cash and Counseling - consumer-directed care model.<br><br>Participants has the opportunity to receive a monthly allowance that they could use to hire workers of their own choosing and to purchase care related services and goods. | Received personal care services (PCS) or home- and community-based services (HCBS) as usual | • Satisfaction with services/care<br>• Unmet needs<br>• Annual Expenditures<br>• Cost per recipient per month<br><br>Caregiver outcomes<br>• Hours of care provided<br>• Caregiver satisfaction<br>• Caregiver well-being |

| | | | | | | | | |
|---|---|---|---|---|---|---|---|---|
| | | | | Florida: Adults with physical and developmental disabilities, children with developmental disabilities | New Jersey (T) 345, (C) 337, Florida adults (T) 419, (C) 392, Florida children (T) 441, (C) 418.<br><br>Arkansas 67.6% female, Florida children 37% female, Florida adults 45.4% female, New Jersey 66.1% female.<br><br>Arkansas 18-39 27.5%, 40-64 72.5%,<br>New Jersey 18-39 33.8%, 40-64 66.2%,<br>Florida children 3-12 71.2%, 13-17 28.8%, Florida adults 18-39 75.5%, 40-59 24.5%. | Duration: 9 months | | |

| | | | | | | | | |
|---|---|---|---|---|---|---|---|---|
| 4. | Caldwell et al. (2007) | Longitudinal comparative before-after study.<br><br>(Controlled before-after study) | Illinois, U.S.A. | Developmental disabilities | 87 participating families<br>Treatment: 38<br>Comparison: 49<br><br>50% female (Treatment)<br>49% female (control), total 49.4%<br><br>Mean age:<br>36.59 (treatment)<br>27.78 (control) | Consumer-directed care program.<br><br>Families were provided with an individualised budget. Individuals with disabilities and their families developed a plan with the assistance of a service facilitator and decided what services and supports to purchase. State serves as fiscal agent.<br><br>Duration: 9 years | Families on the waiting list for the program, who may have received some limited services and/or purchases services out-of-pocket | • Unmet needs<br><br>• Service satisfaction |
| 5. | Carlson et al. (2005) | Randomised controlled trial | 3 sites in U.S.A.<br>Arkansas, Florida, New Jersey | Various<br><br>Arkansas: physical disabilities (may also have cognitive disabilities)<br><br>New Jersey: Physical disabilities | Total participants (adults): 1,966: Arkansas (T) 243, (C) 230, New Jersey (T) 345, (C) 337, Florida adults (T) 419, (C) 392; | Cash and Counseling - consumer-directed care model.<br><br>Participants has the opportunity to receive a monthly allowance that they could use to hire workers of their own choosing and | Received personal care services or home-and community-based services as usual. | • Satisfaction with services/care<br><br>• Quality of life<br><br>• Unmet needs |

| | | | | | | | | |
|---|---|---|---|---|---|---|---|---|
| | | | | (and perhaps cognitive disabilities)<br><br>Florida: Adults with physical and developmental disabilities | Arkansas 67.6% female, New Jersey 65.1% female, Florida adults 45.4% female;<br><br>Mean age not reported, Arkansas 18-39yrs 27.1%, 40-64yrs 72.9%,<br>New Jersey 18-39yrs 34.9%, 40-64yrs 65.1%, Florida adults 18-39yrs 75%, 40-59yrs 25%; | to purchase care related services and goods.<br><br>Duration: 9 months | | |
| 6. | Conroy et al. (2002) | Controlled before-after study | California, U.S.A.<br>Three sites: Eastern Los Angeles (ELARC), Redwood Coast(RCRC), and Tri-Counties (TCRC) | Primarily intellectual disabilities (mental retardation), with secondary major disabilities including physical disabilities and mental illness. | Participants 77.<br>Treatment group 63, control group 14.<br><br>% female: Treatment group 28.6%, control group 35.7% | Self-determination<br><br>Each participant received an individual budgets and a person-centred plan was developed<br><br>Duration: 2 years | A group of people and families who wanted to participate but had to wait (from site ELARC) – traditional service system | • Perception of quality of life<br>• Choice<br>• Adaptive and challenging behaviours |

| | | | | | | | | |
|---|---|---|---|---|---|---|---|---|
| | | | | | Age: Treatment group mean 25.4, control group 27.9 | | | |
| 7. | Cook (2019) et al. | Randomised controlled trial | Texas, U.S.A. | Mental illness | 216 participants, Treatment: 114 Control: 102<br><br>Female: 62%<br><br>Age: ≥18, mean 41.6, SD 9.7 | Self-directed care by means of an individual budget.<br><br>Participants developed person-centered plans for recovery and created individual budgets for the purchase of services and goods corresponding to plan goals.<br><br>Duration: 24 months | Services as usual | • Self-perceived recovery<br>• Psychosocial status<br>• Psychiatric and somatic symptoms<br>• Satisfaction with services<br>• Cost of services |
| 8. | Croft et al. (2019) | Uncontrolled before-after study | Pennsylvania, U.S.A. | Mental health conditions | Participants: 45<br><br>71.1% female<br><br>Average age 51.5 | Self-direction<br><br>Participants “banked” funds by intentionally reducing their use of some mental health services and | n/a | • Service use<br>• Standardised monthly costs |

| | | | | | | | | |
|---|---|---|---|---|---|---|---|---|
| | | | | | | applied the cost savings towards flexible spending of approved nonclinical good and services.<br><br>Duration: study used 3 years of administrative data. Participants were enrolled on average 3.46 years. | | |
| 9. | Croft et al. (2020) | Quasi-experimental before-after comparative study.<br><br>(Controlled before-after study) | Utah, U.S.A. | Mental health conditions | After matching: 623. Treatment: 94, control 529.<br><br>Female: treatment 38.3%, control 38.37%.<br><br>Mean age: treatment 42.38, control 42.85. | Self-direction.<br><br>Participants created a person-centred plan and allocated funds from a flexible budget to meet recovery goals.<br><br>Participants enrolled in the program an average of 199 days | Received traditional Medicaid-funded and state-funded services. | • Service use |
| 10. | Dale et al. (2004) | Randomised controlled trial | Florida, U.S.A. | Children with developmental disabilities | Total participants: 1002, Treatment 501, Control: 501; | Cash and Counseling - consumer-directed care model. | Received traditional waiver services. | • Costs |

| | | | | | | | | |
|---|---|---|---|---|---|---|---|---|
| | | | | | % female Treatment: 38,1%, Control 35,9%;<br><br>< 12yrs Treatment : 63,5%, Control: 64,1%. | Parents of treatment group members were given the opportunity to receive a monthly allowance they could use to hire their choice of caregivers or to buy other services or goods to meet their child's care needs.<br><br>Duration: 9 months | | |
| 11. | Dale & Brown (2005) | Randomised controlled trial | 3 sites in U.S.A. Arkansas, Florida, New Jersey | Various<br><br>Arkansas: physical disabilities (may also have cognitive disabilities)<br><br>New Jersey: Physical disabilities (and perhaps cognitive disabilities) | Total participants (adults): 2,282.<br><br>Arkansas: 556, New Jersey: 813, Florida adults: 913;<br><br>% of female: Arkansas 67.6%, New Jersey 66.1%, Florida adults 45.3%; | Cash and Counseling - consumer-directed care model.<br><br>Consumer-directed care where participants receive a monthly allowance to hire workers of their own choosing and to purchase care related services and goods.<br><br>Duration: 9 months | Received personal care services or home- and community-based services as usual. | • Costs<br><br>• Satisfaction with services/care<br><br>• Unmet needs. |

| | | | | | | | | |
|---|---|---|---|---|---|---|---|---|
| | | | | Florida: Adults with physical and developmental disabilities | Mean age not reported, Arkansas 18-39yrs 27.5%, 40-64yrs 72.5%, New Jersey 18-39yrs 33.7%, 40-64yrs 66.31%, Florida adults 18-39yrs 75.5%, 40-59yrs 24.5%; | Follow-up : 1 to 2 years | | |
| 12. | Fontecedro (2020) | Observational comparative cross-sectional study | Trieste, Italy | Mental health conditions | 128 participants: 67 treatment, 61 control<br><br>Female: treatment 37.3%, control 45.9%<br><br>Age: 20+, 20-59: (T) 74.6% (C) 65.4% | Individual health budget.<br><br>Participants received an individual health budget<br><br>Duration: not stated how long participants had been in receipt of budget | Care maintained as usual | • Health of the Nation Outcome Scale |
| 13. | Forder et al. (2012) | Non-randomised controlled trial | 20 in-depth pilot sites in England – 8 choose | Mental health – other chronic conditions considered but | 197 participants with mental health | Personal health budgets. | Continuing conventional support arrangement | • Care-related quality of life |

| | | | | | | | | |
|---|---|---|---|---|---|---|---|---|
| | | | mental health conditions – Yorkshire and Humber, West Midlands, South East Coast, South East Coast, East Midlands, East of England, South West, North West | not reported in this review | problem completed the 12 month follow-up questionnaire. Treatment: 105 Control: 92<br><br>Treatment: 49% female. Control: 50% female.<br><br>Treatment: mean 45yrs, 11% over 75. Control: mean 53yrs, 10% over 75. | Five different implementation models used, varying according to: whether the budget is known before support planning; what flexibility there is in terms of what help can be purchased; and the choice of deployment.<br><br>Duration: main follow-up at 12 months | for their condition | • Health-related quality of life<br><br>• Psychological well-being<br><br>• Subjective well-being<br><br>• Changes in costs<br><br>• Cost effectiveness |
| 14. | Foster et al. (2003) | Randomised controlled trial | Arkansas, U.S.A. | Physical disabilities (may also have cognitive disabilities) | Survey of 1,433 carers;<br><br>39 or younger 22.5%, 40-64 yrs 64.1%, 65 or older 13,5%. | Cash and Counseling - consumer-directed care model.<br><br>Consumer-directed care where participants receive a monthly allowance to hire workers of their own choosing and to purchase care | Received personal care services or home- and community-based services as usual. | • Hours of care provided<br><br>• Satisfaction<br><br>• Well-being |

| | | | | | | | | |
|---|---|---|---|---|---|---|---|---|
| | | | | | | related services and goods.<br><br>Duration: 9 months | | |
| 15. | Foster et al. (2004) | Randomised controlled trial | Florida, U.S.A. | Children with developmental disabilities | Total participants (children) :859, Treatment : 441, Control 418 ;<br><br>Treatment : 38,5% female, Control : 35,2% female;<br><br>< 12yrs Treatment : 63,3%, Control : 63,4%. | Cash and Counseling - consumer-directed care model.<br><br>Parents of treatment group members were given the opportunity to receive a monthly allowance they could use to hire their choice of caregivers or to buy other services or goods to meet their child's care needs. | Received traditional waiver services. | • Satisfaction with child's care<br><br>• Child's unmet needs<br><br>• Child's quality of life. |
| 16. | Glendinning et al. (2008) | Randomised controlled trial | 13 pilot sites in England: Sussex, Barking and Dagenham, Barnsley, Bath and North East Somerset, Coventry, | Mixed and pilot site-dependent: physical disability, learning disability, mental health conditions | 959 participants, Treatment: 510 Control: 449<br><br>Female: 56%<br><br>Age: adults, mean 57 | Individual budget<br><br>Participants received an individual budget in addition to traditional social care services. | Continued to receive traditional social care support | • Perceived quality of life<br><br>• Psychological well-being<br><br>• Social care outcomes (ASCOT)<br><br>• Self-perceived health |

| | | | | | | | | |
|---|---|---|---|---|---|---|---|---|
| | | | Essex, Gateshead, Kensington and Chelsea, Leicester city, Lincolnshire, Manchester, Norfolk, Oldham. | The study also includes older people | | Duration: 6 months between baseline and follow-up | | • Satisfaction with services<br>• Costs<br>• Cost-effectiveness |
| 17. | Glendinning et al. (2009) | Randomised controlled trial | 9 pilot sites in England: Sussex, Bath and North East Somerset, Essex, Gateshead, Kensington and Chelsea, Lincolnshire, Norfolk, Oldham.<br><br>One is not mentioned | Mixed and pilot site-dependent: physical disability, learning disability, mental health conditions<br><br>The study also includes older people | Total careers : 129, Treatment : 69, Control : 60,<br><br>Physical disabilities (T) 8, (C) 11, Older people (T) 16, (C) 17, Learning disabilities (T) 32, (C) 38, Mental health conditions (T) 4, (C) 3<br><br>All ⩾25yrs, 45-59yrs, (T) 57%, (C) 58%, ≥60yrs, (T) 32%, (C) 36%. | Individual budget<br><br>Participants received an individual budget in addition to traditional social care services.<br><br>Duration: 6 months between baseline and follow-up | Continued to receive traditional social care support | For careers<br>• Quality of life<br>• Well-being<br>• Social care outcomes (ASCOT)<br>• Impact<br>• Self-perceived health<br>• Satisfaction with services |

| | | | | | | | | |
|---|---|---|---|---|---|---|---|---|
| 18. | Hagglund et al. (2004) | Controlled cross-sectional study | Missouri, U.S.A. | Physical disabilities | 114 participants. Treatment: 61, Control 53<br><br>32% female<br><br>Average age: 48 years | Consumer-directed personal assistance services.<br><br>Consumers hired and managed their own personal assistants.<br><br>Duration: minimum 1 month enrolment | Received services through an agency-directed model. | • Unmet need<br>• Satisfaction<br>• Quality of Life |
| 19. | Leuci et al. (2021 | Longitudinal comparative before-after study | Parma, Italy | Mental health conditions | 104 participants Treatment : 49 Control : 55<br><br>Average age year : 28 | Personal Health Budget within a specific programme.<br><br>Duration: 2 years between baseline and follow-up | Continued to receive traditional social care support within this specific programme | • Brief Psychiatric Rating Scale<br>• Global Assessment of Functioning<br>• Health of the Nation Outcome Scale |
| 20. | Pelizza et al. (2022) | Uncontrolled before-after study | Parma, Italy | Mental health conditions | 137 participants<br><br>Average age year : 33 | Personal Health Budget<br><br>Duration: 2 years between baseline and follow-up | n/a | • Brief Psychiatric Rating Scale<br>• Global Assessment of Functioning<br>• Health of the Nation Outcome Scale |
| 21. | Shen et al. (2008) | Randomised controlled trial | New Jersey, U.S.A. | Mental health conditions | Total participants : 228 Treatment: 109, | Cash and Counseling - consumer-directed care model. | Received personal care services or home-and | • Satisfaction with services<br>• Satisfaction with quality of life |

| | | | | | | | | |
|---|---|---|---|---|---|---|---|---|
| | | | | | Control 119;<br><br>Treatment: 77% female, Control: 64% female;<br><br>(T) 18-39yrs 31%, 40-64yrs 69%, (C) 18-39yrs 29%, 40-64yrs 71%; | Consumer-directed care where participants receive a monthly allowance to hire workers of their own choosing and to purchase care related services and goods.<br><br>Duration: 9 months | community-based services as usual. | |
| 22. | Wiener et al. (2007) | Controlled cross-sectional survey | Washington State, U.S.A. | Mixed (physical disabilities, mental retardation and developmental disabilities) | Participants 229, Treatment group 124, control 105.<br><br>Female 62.9%<br><br>Age: 28% younger than 44, 72% age 45-64. | Consumer-directed personal assistance services.<br><br>Consumer were responsible for hiring, orienting, supervising, and finding replacements for the caregiver. | Receiving agency-directed care | • Satisfaction with services (Paid Personal Assistance) |
| 23. | Woolham & Benton (2013) | Controlled cross-sectional survey | England, Single local authority | Mixed disabilities: mental health problems, learning disabilities, physical disabilities | Participants 402 (excluding over 65s). Treatment: 126, control 276. | Personal budget<br><br>Participants developed a support plan, with or without support from others, detailing the services and forms | Receiving traditional services. | • Psychological well-being and mental distress (GHQ)<br><br>• Activities of daily living (ability to carry out everyday activities)<br><br>• Mean package costs |

| | | | | | | | | |
|---|---|---|---|---|---|---|---|---|
| | | | | | Total sample including over 65s:<br>Female: treatment 66.1%, control 35.8%.<br><br>Total sample including over 65s:<br>Mean age: treatment 51.5, control 54.9. | of support to be purchased with the funding. | | • Cost effectiveness |

**Table S11.** Article outcomes reported

| Article | Quality of life | Health | Satisfaction with services | Service uses | Unmet needs | Costs |
|---|---|---|---|---|---|---|
| *Physical disabilities* | | | | | | |
| Beatty et al. (1998) | - | - | ✓ | - | - | - |
| Hagglund et al. (2004) | ✓ | - | ✓ | - | ✓ | - |
| *Intellectual and developmental disabilities* | | | | | | |
| Caldwell et al. (2007) | - | - | ✓ | - | ✓ | - |
| Conroy et al. (2002) | ✓ | - | - | - | - | - |
| Dale et al. (2004) | - | - | - | - | - | ✓ |
| Foster et al. (2004) | ✓ | - | ✓ | - | ✓ | - |
| Caldwell et al. (2007) | - | - | ✓ | - | ✓ | - |
| *Mental health disabilities* | | | | | | |
| Cook et al. (2019) | ✓ | - | ✓ | - | - | ✓ |
| Croft et al. (2019) | - | - | - | ✓ | - | ✓ |
| Croft et al. (2020) | - | - | - | ✓ | - | - |
| Fontecedro et al. (2020) | ✓ | - | - | - | - | - |
| Forder et al. (2012) | ✓ | - | - | - | - | ✓ |
| Leuci et al. (2021) | ✓ | - | - | - | - | - |
| Pelizza et al. (2022) | ✓ | - | - | - | - | - |
| Shen et al. (2008) | ✓ | - | ✓ | - | - | - |
| *Mixed disabilities* | | | | | | |
| Benjamin et al. (2000) | - | - | ✓ | - | ✓ | - |
| Brown et al. (2007) | - | - | ✓ | - | ✓ | ✓ |
| Carlson et al. (2005) | ✓ | - | ✓ | - | ✓ | |
| Dale & Brown (2005) | - | - | ✓ | - | ✓ | ✓ |

| | | | | | | |
|---|---|---|---|---|---|---|
| Glendinning et al. (2008) | ✓ | ✓ | ✓ | - | - | ✓ |
| Wiener et al. (2007) | - | - | ✓ | - | - | - |
| Woolham & Benton (2013) | ✓ | ✓ | - | - | - | ✓ |

Table S12**.** Article outcome measures and findings

| | **Articles** | **Outcomes** | |
|---|---|---|---|
| | | **Measures** | **Findings** |
| 1. | Beatty (1998) | • Satisfaction with attendant services<br>*(Calculated using a 16-item Personal Assistance Satisfaction Index covering a range of issues regarding satisfaction with the delivery of PAS, including cost of services, control over assistants' schedule, availability of assistants, safety, and consumer-assistant interactions. Each item was ranked on a 5-point Likert scale, with 1 representing "not at all satisfied" and 5 representing "extremely satisfied." An overall satisfaction score (range of 16 to 80) was computed by adding the responses of all 16 items. Chronbach's alpha of 0.88.)* | • People receiving consumer-directed PAS had a significantly higher average total satisfaction score (61.4, SD 9.7) than those receiving non-consumer-directed services (52.1, SD 10.9), t=4.17, p<0.001. Those in the consumer-directed group were significantly more likely to be "extremely" or "very" satisfied than their waiting list counterparts on 6 individual items: (1) costs of personal assistance (85%, 59%, p=0.006), (2) choice and control over personal assistant (88.3%, 46.9%, p=0.000), (3) control over assistant's work schedule (81.7%, 53.1%, p=0.004), (4) authority to direct personal assistant (93.3%, 59.4%, p=0.001), (5) availability of personal assistant regardless of time/day (46.7%, 21.9%, p=0.02), (6) availability of assistant in an emergency (53.3%, 31.3%, p=0.043).<br>The difference in satisfaction with cost is largely because people on the waiting list had to pay out of pocket for their services. To control for this possibility, the overall score was re-computed after subtracting the item for satisfaction with cost. The difference in overall satisfaction remained significant, with an average score of 57.1 for those in the consumer-directed program, and 48.6 for those on the waiting list (t=3.97, p<0.001). |
| 2. | Benjamin (2000) | • Unmet need<br>*(1. unmet ADL needs: range 0-6, number of ADL needs unmet due to not having help, 2. unmet* | • Higher score means fewer needs unmet. |

| | | | |
|---|---|---|---|
| | | *IADL needs: range 0-5, number of IADL needs unmet due to not having help)*<br><br>• Service satisfaction<br>*(Five domains: (1) Technical quality, range 5-25, P competent, well-trained, services perfect, P appreciates direction, P makes home orderly, (2) Provider shortcomings, range 3-15, P needs to respect C, P needs to listen, hurries too much, P frequently late, (3) Service impact, range 2-10, P services make it easier to do things inside the home and outside the home, (4) General satisfaction, range 2-10, C satisfied with how P meeting personal care needs and housekeeping needs, (5) Interpersonal manner, range 2-9, Closeness of relationship with P; C can share feelings with P.)*<br>P=provider, C=Client | Unmet ADL needs: agency-directed participants scored higher, meaning they reported fewer unmet needs (consumer = 5.07 (sd 1.54), agency = 5.38 (sd 1.21), p= 0.000) and the difference was statistically significant.<br>Unmet IADL needs: consumer-directed participants scored higher 4.37 (sd 1.24), had fewer unmet needs than agency-directed 4.28 (sd 1.18), but the difference was not statistically significant, p=0.199.<br><br>• Higher mean score indicates more satisfaction<br>The difference between groups was significant for 4 of the 5 outcome domains, and in all 4 domains the consumer-directed participants rated services more positively than the agency-directed users:<br>- Technical quality: consumer= 20.90 (sd 3.31), agency = 20.07 (3.82), p=0.000<br>- Service impact: consumer= 8.09 (sd 1.98), agency = 7.63 (sd 1.96), p=0.000<br>- General satisfaction: consumer= 9.06 (sd 1.65), agency = 8.66 (sd 2.07), p=0.000<br>- Interpersonal manner: consumer= 7.45 (1.80), agency = 6.43 (sd 1.92), p=0.000<br>For the domain of Provider Shortcomings, ratings were similar and not significant: consumer= 10.64 (3.47), agency = 10.65 (2.91), p=0.984. |
| 3. | Brown (2007) Final report - Cash and Counseling Demonstration (includes multiple reports): Carlson(2005), | **User** outcomes<br><br>• Satisfaction with services or care<br>*(measured from interview responses, most were binary or 4-point scales collapsed into binary measures. Means were predicted using logit models. Topics covered include (1) satisfaction with caregiver's schedule (2) satisfaction with relationship, (3) satisfied with the way caregiver* | <br><br>• (1) For adults in each state, the proportion reporting that they were very satisfied with their caregiver's schedule was significantly higher for the treatment group: Arkansas (T=85.2, C=66.9, D=18.3, p=0.000), New Jersey (T=73.4, C=56.8, D=16.6, p=0.000), adults in Florida (T=83.4, C=70.9, D=12.5, p=0.002), (Carlson, 2005). The proportion of children in Florida reporting that they were very satisfied with the caregiver's schedule was significantly higher for the treatment group (T = 85.3, C = 63.9, D = 21.4, p=0.000), (Foster, 2004). |

| | | | |
|---|---|---|---|
| | Dale (2005),<br>Dale (2004),<br>Foster (2003)<br>Foster (2004),<br>Shen (2008). | *helped with Daily Living Activities, (4) satisfied with the way caregiver helped around the house/community, (5) satisfied with the way caregiver helped with routine health care, (6) satisfied with overall care arrangements* | (2) For adults in each state, the proportion reporting that they were very satisfied with the caregiver relationship was significantly higher for the treatment group: Arkansas (T=95.0, C=78.5, D=16.5, p=0.000), New Jersey (T=89.7, C=78.4, D=11.4, p=0.001), adults in Florida (T=94.4, C=83.2, D=11.1, p=0.002). (Carlson, 2005). The proportion of children in Florida reporting that they were very satisfied with the caregiver relationship was significantly higher for the treatment group (T = 96.0, C = 82.4, D = 13.5, p=0.000), (Foster, 2004).<br>(3) For adults in each state, the proportion reporting that they were very satisfied with the way caregivers helped with Daily Living Activities was significantly higher for the treatment group: Arkansas (T=95.9, C=75.7, D=20.2, p=0.000), New Jersey (T=82.8, C=69.6, D=13.2, p=0.001), adults in Florida (T=92.0, C=65.4, D=26.6, p=0.000). (Carlson, 2005).<br>(4) For both adults and children, the proportion reporting that they were very satisfied with the way caregivers helped around the house/community was significantly higher for the treatment group in each state: Arkansas (T=90.4, C=64.0, D=26.4, p<0.001), New Jersey (T=84.4, C=66.0, D=18.4, p<0.001), adults in Florida (T=85.4, C=70.9, D=14.5, p=0.001), children in Florida (T=85.3, C=73.1, D=12.3, p<0.001).<br>(5) For adults in Arkansas and Florida, the proportion reporting that they were very satisfied with the way caregiver helped with routine health care was significantly higher for the treatment group: Arkansas (T=92.2, C=74.7, D=17.5, p=0.000), , adults in Florida (T=91.8, C=79.3, D=12.5, p=0.007). The proportion of the treatment group in New Jersey reporting being very satisfied was also higher, but not significantly so (T=86.5, C=80.9, D=5.6, p=0.153). (Carlson, 2005).<br>(6) For both adults and children, the proportion reporting that they were satisfied with overall care arrangements was significantly higher for the treatment group in each state: Arkansas (T=71.0, C=49.1, D=29.2, p=0.000), New Jersey (T=51.9, C=35.0, D=16.9, p=0.000), adults in Florida (T=68.2, C=48.0, D=20.2, p=0.000), children in Florida (T=56.4, C=26.8, D=29.7, p<0.001).<br><br>• (1) For both adults and children, significantly fewer people in the treatment group had unmet needs for help with Daily Living Activity in each state: |

| | | | |
|---|---|---|---|
| | | • Unmet needs for help with (1) Daily Living Activities, (2) household activities, (3) transportation, (4) routine health care<br><br>-----------------------------------<br>**Caregiver** outcomes | Arkansas (T=25.8, C=41.0, D=-15.2, p=0.001), New Jersey (T=46.1, C=54.5, D=-8.4, p=0.028), adults in Florida (T=26.7, C=33.8, D=-7.1, p=0.014), children in Florida (T=32.8, C=44.6, D=-11.8, p<0.001).<br>(2) For adults in each state, people in the treatment group were significantly less likely to report unmet needs of household activities (Carlson, 2005): Arkansas (T=41.3, C=56.0, D=-14.7, p=0.002), New Jersey (T=55.7, C=62.2, D=-6.5, p=0.084), adults in Florida (T=35.5, C=43.8, D=-8.2, p=0.014). For children in Florida, those in the treatment group were significantly less likely to report unmet needs for help doing things around the house (T = 38.0, C = 54.9, D= 17.0, p=0.000), (Foster, 2004).<br>(3) For adults in each state, people in the treatment group were significantly less likely to report unmet needs with transportation: Arkansas (T=27.0, C=47.2, D=-20.2, p=0.000), New Jersey (T=46.2, C=54.1, D=-7.9, p=0.037), adults in Florida (T=32.2, C=38.5, D=-6.3, p=0.057). (Carlson, 2005). For children in Florida, those in the treatment group were significantly less likely to report unmet needs for help with transportation (T = 28.1, C = 37.2, D= -9.2, p=0.004), (Foster, 2004).<br>(4) Adults in New Jersey and Florida in the treatment group were significantly less likely to report unmet needs with routine health care: New Jersey (T=37.0, C=50.5, D=-13.6, p=0.000), adults in Florida (T=16.8, C=23.9, D=-7.1, p=0.011). People in the treatment group in Arkansas reported lower unmet needs with routine health care, but the difference was not significant (T=26.6, C=32.2, D=-5.7, p=0.189), (Carlson, 2005). For children in Florida, those in the treatment group were significantly less likely to report unmet needs for help with routine health care (T = 22.1, C = 32.1, D= -10.0, p=0.001), (Foster, 2004).<br><br>-----------------------------------<br><br>• Arkansas is the only site for which outcomes were provided for caregivers of young adults. Live-in caregivers provided significantly fewer hours of care |

| | | | |
|---|---|---|---|
| | | • Hours of care provided<br>*(calculated from the total hours of care provided in 2 week reference period, for (1) live-in caregivers, and (2) visiting caregivers)*<br><br>• Caregiver satisfaction<br>*(percent satisfied with care recipient's overall care arrangement)*<br><br>• Caregiver wellbeing<br>*(based on indicators of (1) emotional strain, (2) financial strain, (3) physical strain)*<br><br>----------------------------------<br><br>**Mental Health** user outcomes<br><br>• Satisfaction with caregiver | for those in the treatment group compared with the control group (T = 139.6, C = 34.9, D = -23.8, p=0.095). For visiting caregivers, they provided fewer hours of care but the difference was not significant ( T = 34.9, C = 41.5, D = -6.6, p = 0.409). (Foster, 2003)<br><br>• Arkansas is the only site for which outcomes were provided for caregivers of young adults. A significantly higher percent of caregivers in the treatment group reported being very satisfied with care (T = 60.1, C = 39.2, D = 20.9, p=0.000). (Foster, 2003)<br><br>• Arkansas is the only site for which outcomes were provided for caregivers of young adults. On all three indicators, caregivers of the treatment group reported significantly lower levels of strain (%). Emotional ( T= 22.2, C = 34.2, D = -12.0, p=0.012), Financial (T = 19.8, C = 35.7, D = -15.9, p=0.001), Physical (T = 20.9, C = 38.4, D = -17.5, p= 0.009). (Foster, 2003)<br><br>---------------------------------<br><br>• Consumers in the treatment group had a significantly higher likelihood of reporting that they were satisfied with the caregiver's schedule compared with those in the control group (Odds Ratio: 3.25, p<0.01).<br>Consumers in the treatment group had a significantly higher likelihood of reporting that they were satisfied with caregiver's help around the house and community (Odds Ratio: 3.21, p<0.05).<br>Consumers in the treatment group were more significantly more likely to report being very satisfied with overall care arrangement (OR: 4.13, p<0.05). (Shen,2008) |

| | | | |
|---|---|---|---|
| | | • Unmet needs<br>*(needs help with household activities, needs help with routine health care)* | • Consumers in the treatment group were significantly less likely than the control group to claim unmet needs for routine health care (Odds ratio: 0.43, p<0.01). (Shen,2008)<br>------------------------------------ |
| | | ------------------------------------<br>**Costs**<br>• Annual expenditure for Medicaid and Medicare services<br>(Medicaid expenditure broken down personal and non-personal care) | • For nonelderly adults in Arkansas, Medicaid personal care expenditure was significantly higher for the treatment group (T=$5,435, C=$2,430, D=$3,005, p=0.000), but this was offset by significantly lower non-personal care (T=$8,689, C=$10,432, D=$-1,743, p=0.035), mainly due to significantly lower expenditures on Medicaid nursing facility and inpatient care. The total combined Medicaid and Medicare expenditure was higher for the treatment group but the difference was not significant (T=$20,111, C=$18,747, D=$1,365, p=0.418)<br>For nonelderly adults in New Jersey, Medicaid personal care expenditure was significantly higher for the treatment group (T=$11,166, C=$9,220, D=$1,946, p=0.000). Non-personal care expenditure was lower for the treatment group but not significantly so. Medicaid Home health care was the only significantly lower expenditure for the treatment group, compared with the control group, in non-personal care. The total combined Medicaid and Medicare expenditure was higher for the treatment group but the difference was not significant (T=$37,749, C=$36,394, D=$1,355, p=0.591).<br>For nonelderly adults in Florida, Medicaid waiver expenditures was significantly higher for the treatment group (T=$22,017, C=$18,321, D=$3,696, p=0.000), Non-waiver expenditure was lower, but not significantly. The only service expenditure that was significantly lower for the treatment group was Medicare nursing facilities. The total combined Medicaid and Medicare |

| | | | |
|---|---|---|---|
| | | | expenditure was significantly higher for the treatment group (T=$29,867, C=$26,849, D=$3,018, p=0.02). (Dale,2005) |
| | | • Medicaid expenditures for children in Florida | • For the first year of enrolment, the difference in waiver expenditures for the treatment group ($15,966) was significantly higher than the control group ($12,647), D=$3,319, p=0.000. However, most of this increase was offset by a significantly lower expenditure in home health services by the treatment group (T=$6,393, C=$7,968, D=-$1,574, p=0.055). The difference in total annual average Medicaid expenditures for the treatment group ($29,974) and the control group ($29,095) was small (880) and not statistically significant (p=0.476). In the second year of enrolment, the difference in total annual average Medicaid expenditures for the treatment group ($33,458) and the control group ($30,877) was larger ($2,581) and the difference was statistically significant (p=0.082). There were significantly larger savings in home health expenditures (T=$6,361, C=$8,402, D=$-$2,041, p=0.050) but also a significantly larger difference in waiver service expenditures (T=$18,859, C=$14,046, D=$4,812, p=0.000). (Dale, 2004). |
| | | • Costs per recipient per month | • Costs per recipient per month were significantly higher for the treatment group in each state and for both adults and children. Arkansas (T = $513, C = $422, D = $91, p<0.001), New Jersey (T = $1,153, C = $1,106, D = $47, p=0.43), adults in Florida (T = $1,884, C = $1,593, D = $291, p<0.001), children in Florida (T = $1,378, C = $1,099, D = $279, p<0.001). |

| | | | |
|---|---|---|---|
| 4. | Caldwell (2007) | **Family** outcomes<br><br>• Unmet service needs<br>*(measured using a modified version of the Family Support Index) which includes a list of 28 common types of services used. Families were asked if they used each service, if they were not using a service they were asked if they needed it.)*<br><br>• Service satisfaction<br>*(measured using a 5 item questionnaire (e.g. to what degree do you get the service you need?), each item marked on a 5-point scale from 1 (not at all) to 5 (vey much). Alpha reliability at Time 3 was 0.96)* | • At time 3 (9 year point), families in the program had significantly fewer unmet needs (mean 3.11, sd 3.30) compared with families on the waiting list (mean 7.00, sd 5.31). There was a significant decrease in unmet needs between Times 1 and 3 for program participants, t(1,37)=4.02, p<0.01 [Time 1: 6.16 (sd 4.41), Time 2: 3.37 (sd 4.06), Time 3: 3.11 (sd 3.30)]. For families in the program, unmet service needs significantly decreased over time for five services: occupational therapy, social/recreational activities, educational/vocational training, assistance obtaining benefits, and assistance obtain vocational services. At Time 3, compared with families on the waiting list, families in the program had significantly fewer unmet needs on 15 of the 28 services considered (Table 4, for more details).<br><br>• At time 3, families in the program were significantly more satisfied with services (mean 3.89, sd 0.85) compared with families on the waiting list (mean 2.82, 1.25). There was a significant increase in service satisfaction between Times 1 and 3 for program participants, t(1,36)=-5.40, p<0.01, [Time 1: 2.94 (sd 1.10), Time 2: 4.10 (sd 0.53), Time 3: 3.89 (sd 0.85)]. |
| 5. | Conroy (2002) | • Choice making<br>*(measured using the Decision Control Inventory, composed of 35 ratings of the extent to which life decisions are made by paid staff versus user and/or friends and relatives. Scored from 0-10, where 0 means the choice is made entirely by paid staff and 10 means the choice is made entirely by the user. Interrater reliability of the Inventory is 0.86. The 35 scores are combined into a single* | • Participants in all 3 pilot site significantly increase the power held by themselves or their allies. Members of the control group did not.<br>TCRC: Before 78.3, Now 83.5, Change 5.2, p<0.05<br>RCRC: Before 85.9, Now 95.6, Change 9.7, p<0.05<br>ELARC: Before 83.8, Now 85.0, Change 1.2, p<0.05<br>All SD sites: Before 82.9, Now 88.2, Change 5.3, p<0.01<br>ELARC comparison: Before 77.3, Now 77.5, Change 0.2, p<0.05 |

| | | | |
|---|---|---|---|
| | | *scale ranging from o to 100, with higher score meaning more individual control.)*<br><br>• Perception of Quality of life<br>*(Quality of Life Changes Scale, rated on a 5-point Likert scale, covering 14 dimensions of quality including health, friendships, safety and comfort. The interrater reliability was 0.76.)*<br><br>• Adaptive/challenging behaviours<br>*(measured using the Adaptive Behaviour Scale and the Challenging Behaviour, composed of 14 items detailing various maladaptive behaviours. The table is based on a 100-point scale, with higher scores indicating less challenging behaviour.)* | • Participants in all 3 pilot sites and the comparison group showed statistically significantly increases.<br>TCRC: Before 70.0, Now 80.3, Change 10.3, p<0.05<br>RCRC: Before 71.3, Now 86.0, Change 14.7, p<0.05<br>ELARC: Before 66.7, Now 77.8, Change 11.1, p<0.05<br>All SD sites: Before 69.6, Now 81.3, Change 12.1, p<0.05<br>ELARC comparison: Before 69.6, Now 78.0, Change 8.4, p<0.05<br><br>• Participants in only 1 site (TCRC) showed a statistically significant increase in the Adaptive Behaviour Scale score. This was not replicated when all SD sites were combined.<br>TCRC: Before 62.0, Now 65.8, Change 3.8, p<0.05<br>RCRC: Before 81.5, Now 74.2, Change -7.3, p>0.05<br>ELARC: Before 56.4, Now 57.3, Change 0.9, p>0.05<br>All SD sites: Before 66.1, Now 65.2, Change -0.8, p>0.05<br>ELARC comparison: Before 64.6, Now 67.8, Change 3.1, p>0.05<br>There were no statistically significant changes in the Challenging Behaviour Scale scores, although each group improved slightly.<br>TCRC: Before 84.4, Now 88.1, Change 3.6, p>0.05<br>RCRC: Before 92.6, Now 93.0, Change 0.4, p>0.05<br>ELARC: Before 82.8, Now 84.4, Change 1.6, p>0.05<br>All SD sites: Before 86.3, Now 88.2, Change 1.9, p>0.05<br>ELARC comparison: Before 84.2, Now 89.6, Change 5.4, p>0.05 |
| 6. | Cook (2019) | • Perceived level of recovery | • Compared with the control group, self-directed care participants improved significantly over time in Recovery Assessment Scale total scores (estimate = |

| | | | |
|---|---|---|---|
| | | *(measured by the 41-item Recovery Assessment Scale with scores on subscales measuring goal and success orientation, personal confidence and hope, reliance on others, willingness to ask for help and not feeling dominated by symptoms.)*<br>*Cronbach's α: baseline total score was 0.93, personal confidence and hope 0.80, goal and success orientation 0.79, willingness to ask for help 0.76, reliance on others 0.73, and not feeling dominated by symptoms 0.60.* | 4.27, p=0.009), and on two of its subscales: goal orientation (estimate = 0.71, p=0.007) and personal confidence (estimate = 1.10, p=0.27). No statistical significance on other subscales. |
| | | • Changes in psychosocial status<br>*(Measured in three areas: (1) self-esteem assessed by a subscale of the Empowerment Scale which measures feelings of self-worth and confidence in general abilities, α=0.97 (2) Participants sense of personal control over important life outcomes assessed by the Coping Mastery Scale, α=0.66 (3) the extent to which participants felt they were being served in autonomy-supportive environments measured by the Perceived Autonomy Support Scale, α=0.92)* | • Compared with the control group, the intervention participants improved significantly over time in level of self-esteem (estimate = 0.90, p=0.031), showed significantly greater improvement over time in coping mastery (estimate = 0.12, p=0.007) and improved significantly over time in the extent to which they perceived their service delivery environment as supportive of their personal autonomy (estimate = 0.29, p=0.03). |
| | | • Reduction in psychiatric and somatic symptoms<br>*(Measured with the Brief Symptom Inventory's Global Severity Index which quantifies an individual's severity of illness, the somatic subscale assess physical manifestations of* | • No significant difference in global severity (estimate = -0.06. p=0.931). Intervention participants had significantly lower somatic symptom severity over time than the control participants (estimate = -2.16, p=0.003 ) |

| | | | |
|---|---|---|---|
| | | *emotional distress such as pain weakness, or shortness of breath.)* | |
| | | • Cost of services<br>*(Service utilization and costs data obtained from the electronic administrative records maintained by the state of Texas's Data Warehouse. Differences in total costs associated with study condition were analysed by using generalized linear models with negative binomial distribution. )* | • The intervention group had lower total mean costs (mean ± sd) per person than control participants in the first year ($2,998 ± 3,128 versus $3,189 ± 4,608) and second year ($2,241 ± 2,960 versus $2,303 ± 4,266) of study participation, and in both years combined ($5,240 ± 5,500 versus $5,493 ± 8,268). Over the 2 years of the program combined: the intervention group spent less on average per person, compared with the control group, on skills training, psychosocial rehabilitation, case management, inpatient hospitalization, psychiatric crisis services, substance abuse treatment, medication management, and medications. The intervention group spent more on average on psychotherapy, peer services and diagnostic services. Only the intervention group could make non-traditional expenditures. Self-directed care participation significantly lowered costs for skills training in year 1 (estimate -0.797, p=0.011), case management in year 2 (estimate -1.07, p=0.033), inpatient services in year 2 (estimate -0.692, p=0.045), and medication management in years 1 (estimate -0.475, p<0.001) and 2 (estimate -0.443, p<0.001) and both years combined (estimate -0.439, p<0.001). Conversely, self-directed care participation significantly increased costs for psychotherapy in year 1 (estimate 1.094, p<0.001) and in both years combined (estimate 1.145, p<0.001). No other service category costs, including total service costs, were statistically significant - being in the treatment group reduced total services costs (estimate: -0.47) but not significantly (p=0.789). |
| | | • Satisfaction with services | • Compared with control group participants, self-directed care participants had significantly higher satisfaction with their mental health services. Total |

| | | | |
|---|---|---|---|
| | | *(Using the Client Satisfaction Questionnaire administered at the 12- and 24-month follow-up points, α=0.95)* | satisfaction scores were significantly higher at both 12 months (t=-2.97, df=175, p=0.003) and 24 months after study baseline (t=-3.75, df=173, p,0.001). At 24 months, 60% (N=56) of intervention participants rated themselves as very satisfied with the mental health services currently received, compared with 38% (N=31) of control group participants, whereas only 10% (N=9) of self-directed care participants reported being very or somewhat dissatisfied with their mental health services, compared with 26% (N=21) of control group participants ($\chi^2$=11.08, df=3, p=0.011). |
| 7. | Croft (2019) | • Service Use (*percent of people with any service use in four service categories: 1. Crisis and inpatient, 2. Mental health clinical outpatient, 3. Mental health community support and coordination, 4. Alcohol and other drug outpatient and community-based services*)<br><br>• Monthly costs (*standardized by the number of months for which data were available, for the four services areas listed above*) | • There were no significant differences in the percentage of individuals who used at least one service in each service category before and after program participation. There were decreases in the percentage using *Mental health clinical outpatient* (97.8% to 91.1%, p=0.375) and *Alcohol and other drug outpatient and community-based services* (20.0% to 13.3%, p=0.375), but it was not significant. The other two service categories showed no change at all.<br><br>• Individuals used significantly fewer *Mental health clinical outpatient* services after program participation (cost: $80.28, SD $129.83, median $47.10) compared to before (cost: $38.45, SD $61.74, median $17.29), mean standardized monthly mental health clinical outpatient costs were $41.83 lower after participation compared to before participation (p<0.001). Standardized monthly costs decreased in all other service categories but none were significant. Note: monthly standardised costs were substantially skewed so both mean and medians were examined using Wilcoxon test. |
| 8. | Croft (2020) | • Service Use (*four service utilization categories: rehabilitation hours, outpatient treatment hours, residential days, and emergency room usage hours.*) | • Self-direction program participants used on average 62.58 more rehabilitation service hours than non-participants (p=0.001).<br>Self-direction program participants used an average of 22.40 more outpatient treatment hours than non-participants (p<0.001). |

| | | | |
|---|---|---|---|
| | | | There were no significant difference between the groups in terms of differences in residential days (8.01 more days for self-direction, p=0.319) or emergency service hours (0.21 less hours for self-direction, p=0.784). |
| 9. | Fontecedro (2020) | • Health of the Nation Outcome Scale (HoNOS), which detects both clinical and psychosocial problems.<br>*It is made up of 12 items that evaluate the extent of problems in the previous 2 weeks.*<br>*1. Hyperactive, aggressive, destructive, or agitated behaviours,*<br>*2. Deliberately self-harming*<br>*3. Problems related to alcohol or drug use*<br>*4. Cognitive problems,*<br>*5. Problems arising from somatic disease or physical disability*<br>*6. Problems related to hallucinations and delusions*<br>*7. Problems related to depressed mood*<br>*8. Other mental and behavioural problems*<br>*9. Relational problems*<br>*10, Problems in the activities of everyday life*<br>*11. Problems in living conditions*<br>*12. Problems in the availability of resources for work and recreation activities*<br>*Each item evaluation on a score scales from 0 (no problem)= to 4 (maximum severity of the problem). A total score can be obtained from the sum of each item's score s and a 4-level severity index by combining responses to the various items (subclinical, mild, moderately severe and, very severity).* | • Four of the 12 items differed significantly between the IHB and comparator groups:<br>- Cognitive problems: IHB (mean 1.58, sd 1.18), C (1.02, 1.24), p=0.01. The IHB group were significantly more likely to have severe to very severe cognitive problems (Odds ratio 1.66, p<0.05)<br>- Problems related to hallucinations and delusions: IHB (mean 1.58, sd 1.36), C (0.91, 1.16), p<0.01. The IHB groups were significantly more like to be at higher risk for severe to very severe problems related to hallucinations and delusions (odds ratio 1.52, p<0.05)<br>- Problems in the activities of everyday life: IHB (mean 2.39, sd 1.09), C (1.53, 1.44), p<0.01. The IHB group were significantly more likely to be at higher risk for moderately severe problems in the activities of everyday life (odds ratio 2.1, p<0.05)<br>- Problems in the availability of resources for work and recreation activities: IHB (mean 0.94, sd 1.09), C (1.69, 1.28), p<0.01. The IHB groups were significantly less likely to be at risk for problems in the availability of resource for work and recreation activities. (odd ratio: 0.51, p<0.05).<br>-Neither the mean scores used to identify clinical severity nor the mean total score differed significantly between the two groups.<br>Total score: IHB (mean 14.13, sd 7.51), C (12.49, 7.91), p=0.23. |

| | | | |
|---|---|---|---|
| 10. | Forder (2012) | • Care-related quality of life<br>*(ASCOT, measures people's achievements of everyday activities including basic capabilities such as dressing and feed, and more complex capabilities such as feeling safe, being occupied and having a sense of control)*<br><br>• Health-related quality of life<br>*(EQ-5D – Euro-QoL, participants rate their health status, how their health has changes, and difficulty carrying out tasks.)*<br><br>• Psychological well-being<br>*(GHQ-12)*<br><br>• Subjective wellbeing<br>*(used a subjective global measure used by ONS in the Integrated Household Survey. The scale considers satisfaction with life, happiness and satisfaction/worries about health.)*<br><br>• Changes in indirect costs<br><br>• Changes in direct costs | • PHB group reported better ASCOT scores, but there was no significant difference associated with the personal heath budget group relative to the control group (0.045, p=0.171), using difference-in-difference method.<br><br>• PHB did not report a greater change in health-related quality of life but there was no significant difference associated with the personal health budget group relative to the control group (-0.04, p=0.105), using difference-in-difference method.<br><br>• PHB group reported poorer GHQ score, but there was no significant difference associated with the personal health budget group relative to the control group (0.0597, p=0.533), using difference-in-difference method.<br><br>• Improvements in subjective well-being reported by PHB group, but no significant difference associated with the personal health budget group relative to the control group (1.255, p=0.289), using difference-in-difference method.<br><br>• The difference in cost changes through time was significant for the mental health group. PHB =-£2980, Control = £70, Change =-£3050, p=0.008. |

| | | | |
|---|---|---|---|
| | | • Changes in total cost<br><br>• Cost-effectiveness – net monetary benefit diff-in-diff (using ASCOT and EQ-5D) | • There was no significant difference between the personal health budget and control group in these cost changes. PHB = £980, Control =£800, Change = £180, p=0.921.<br><br>• There was no significant difference between the personal health budget and control group in total costs changes. PHB = -£2010, Control =£870, Change = -£2880, p=0.199.<br><br>• For the mental health cohort, the average net benefit, using ASCOT, was £4880 greater for people in the personal health budget group compared to people in the control group (p=0.096). Thus, using the ASCOT scale, personal health budgets were cost-effective for the mental health cohort. Using EQ-5D the net benefit was also greater, £1810, but the difference was not significant (p=0.489). |
| 11. | Glendinning (2008)<br>And<br>Glendinning (2009) | **User** outcomes (N.B. randomisation is between IB and comparison, not between user groups so outcomes difference between these groups should not be compared!)<br><br>• Perceived Quality of life<br>*(used a 7-point scale from "so good it could not be better" to "so bad it could not be worse")* | • Perceived quality of life<br>(Total sample = 504 treatment, 439 control). 45% of IB group and 49% of control group report positive outcomes (top 3 scale-points). 17% of IB group and 21% of control group report negative outcome (bottom 3 scale points). (Table 6.3)<br>For mental health users (N = 65 treatment, 64 control), self-reported quality of life was significantly higher for those in the IB group than those in the comparison group (mean = IB 3.78, comparison 4.31, p<0.05, higher score reflect poorer levels of quality of life), but this difference ceases to be significant when proxy responses are removed (total sample size reduces to |

| | | | |
|---|---|---|---|
| | | • Psychological well-being (GHQ-12) *(measured with the 12-item General Health Questionnaire GHQ-12, Cronbach's alpha 0.92)*<br><br>• Social care outcomes *(used the Adult Social Care Outcome Toolkit (ASCOT) which reflects needs for help and outcome gain from services across 7 domains of control, safety, personal care, accommodation, food + nutrition, social participation, occupation. Cronbach's alpha 0.74)*<br><br>• Self-perceived health *(used a 5-point scale from "very bad" to "very good")*<br><br>• Satisfaction with and quality of services | 102). No significant difference between other disability groups (physical = IB 3.93, C 3.83, learning = IB 2.99, C 2.92). (Table 6.4)<br><br>• No statistically significant differences found between IB and control groups, across all disability types. (physical (N=164,134) = IB 14.73, C 15.01, learning (N=96,82) = IB 10.25, C 9.59, mental health (N=56,57) = IB 15.68, C 18.05, all (N=448,380) = IB 13.83 (sd 6.74), C 13.8(sd 6.85)). Higher GHQ scores indicate poorer well-being. (Table 6.4)<br><br>• No statistically significant differences between IB and control groups, across all disability types. (physical (N=169,138) = IB 3.53, C 3.39, learning (N=106.93) = IB 3.80, C 3.81, mental health (N=54,57) = IB 3.16, C 2.97, all (N=457,385) = IB 3.55 (sd 0.79), C 3.48(sd 0.89)). Higher ASCOT scores indicate lower levels of need. (Table 6.4). When comparing the individual domains within ASCOT between the IB and comparison groups, it was found that the IB group was significantly more likely to report feeling in control of their daily lives (48%) compared with the comparison group (41%). No other domains reached statistical significance, even when analysing by disability subgroup.<br><br>• (Total sample = 507 treatment, 446 control). 35% of IB group and 40% of control group report positive outcomes (top 2 scale-points). 28% of IB group and 26% of control group report negative outcome (bottom 2 scale-points). (Table 6.3)<br>No significant difference found between IB and control groups for all disability types. (physical (N=179,146) = IB 3.15, C 3.15, learning (N=118,115) = IB 2.14, C 1.97, mental health (N=66,65) = IB 2.89, C 3.03). Higher scores indicate worse self-perceived health. (Table 6.4) |

| | | | |
|---|---|---|---|
| | | *(based on quality indicators derived from the national User Experience Survey for older home care service users and younger adults. Cronbach's alpha 0.80)* | • Sample: IB 478, C 431. 49% of the IB group and 43% of the comparison group were either extremely or very satisfied with the help they received and this was found to be statistically significant. The result became non-significant when proxies were excluded (N = IB:268, C:288) but the direction of difference remained the same. Differences between IB and comparison groups were not significant for disability subgroups, except for younger physically disabled people in the IB group who were significantly more likely to report higher quality of care (mean 4.91, p<0.05) than those in the comparison group ( 4.14). |
| | | ------------------------------ | ------------------------------ |
| | | **Costs**<br><br>• Social care costs (cost of funding for IB) | • Mean weekly social care cost (N=IB,C):<br>physical (N=90,88) = IB £310, C £334,<br>learning (N=70,63) = IB £359, C £390,<br>mental health (35,33) = IB £149, C £152,<br>all (N=268,250) = IB £279, C £296 … includes older people.<br>Difference between costs were small and not statistically significant. Breakdown of costs by service (same sample numbers as above) showed significantly higher costs for the comparison group (includes older people) in home care (£70) and Independent Living Fund (£30) compared with the IB group (£37 and £8 respectively), p<0.001. The weekly cost of employing a PA was significantly higher in the IB group (£100) compared with the comparison group (£52), p<0.001. The same trend was seen for the physical disability subgroup:<br>home care: IB=£24, C=£82, p<0.001,<br>Indep. Living: IB=£14, C=£39, p<0.01,<br>PA: IB=£144, C=£72, p<0.01 |

| | | | |
|---|---|---|---|
| | | | and in addition, the weekly costs of a social worker or care manager for this subgroup was significantly higher for the IB group (£16) than the comparison group (£8), p<0.001. For learning disabilities the cost of the PA was significantly higher for IB group (£112) than comparison (£58), p<0.05, and the cost of the ILF was significantly lower (IB £11, C £66, p<0.001). There were no significant difference in costs of services between IB and control groups for the mental health subgroup |
| | | • Health care costs | • The mean health cost per week for the IB group was significantly higher (£83) than for people in the comparison group (£59), p<0.05….includes older people! When broken down by health resource, the only significance difference between the two groups was the cost of in-patient stays, IB £33 and C £19, p<0.05. No break down of costs by disability subgroup (subgroups are compared, but not the IB-C breakdown within each group). "*On average, there was a significantly higher health cost per week among older people (mean £107; p< 0.001) compared with people with a physical disability (mean £76), people with a learning disability (mean £23), and people with a mental health problem (mean £76).*" |
| | | • Care and support planning and management | • The average weekly care management cost for the IB group was significantly higher (£18) compared with the comparison group (£11). |
| | | • Cost effectiveness<br>*(examined the mean difference in outcomes (total ASCOT score, GHQ-12 score) over the six-month follow-up period between people in both groups and compared them with the mean difference in costs. The ratio of cost difference to out difference was then computer – the incremental cost-effectiveness ratio (ICER))* | • Using the overall ASCOT score (includes all disability groups), on average, individual budgets look to be cost-effective although the mean value is small and not statistically significant.<br>Using the GHQ score (reversed so that lower scores indicate better psychological well-being), it was costing £250 to achieve an additional one-point gain in psychological well-being. So IBs could be used and would save |

| | | | |
|---|---|---|---|
| | | ------------------------------ | money but would leave users with slightly poorer outcomes. When using scatterplots to look at the variability it was found that, using ASCOT, IBs appear to be marginally more cost-effective than conventionally arranged support. For the GHQ score, results were bunched around the origin (no cost difference, no outcome difference). Table 7.9. "*across all user groups combined there is some evidence that IBs are more cost-effective in achieving overall social care outcomes, but no advantage in relation to psychological well-being*"<br>"*Cost-effectiveness evidence in support of IBs is strongest for mental health service users, on both the outcome measures examined here*". Both mean ratios were negative, although small in size relative to the estimated standard errors and the scatter plots suggest better outcomes at roughly an equivalent cost.<br>"*There appear to be a small cost-effectiveness advantage for IB over standard support arrangements for younger physically disabled people using either of the outcome measures*".<br>"*For people with learning disabilities, there is a cost-effectiveness advantage in terms of social care outcomes but only really when we exclude people without support plans in place from the analysis. In other words, the potential is there to achieve cost-effectiveness, but implementation delays in the pilot sites meant that we did not observe this during the evaluation period. When looking at the psychological well-being outcome, standard care arrangements look slightly more cost-effective than IBs*".<br>------------------------------ |
| | | **Carer** outcomes<br>• Quality of life *(measured on a 7-item scale from "so bad it could not be worse" to "so good it could not be better")* | • Total sample = 60 IB, 69 C). 62% of IB group carers and 38% of control group carers reported positive outcomes (top 3 scale-points). 10% of IB group carers and 11% of control group carers reported negative outcomes (bottom 3 scale points). (Table 6.1) |

| | | | |
|---|---|---|---|
| | | • Well-Being<br>*(used GHQ-12)*<br><br>• Social care outcomes<br>*(five domains of ASCOT are relevant for carers: social participation and involvement, control over daily life, safety. Occupation and employment, caring role)*<br><br>• Self-perceived health<br>*(5-point scale from "very bad" to "very good")*<br><br>• Impact<br>*(measured using the Carers of Older People in Europe Scale - COPE index. The index has 3 components: negative impact o caregiving, positive aspects of caregiving, quality of support, which had internal validity scores of 0.84, 0.62 and 0.73 respectively.)* | Carers who provided assistance to the IB group were significantly more likely to report higher quality of life (mean 4.72, p<0.05) compared with those in the comparison group (mean 4.25)<br><br>• Total sample = 59 IB, 69 C). No statistical difference between IB (mean 12.59, sd 5.42) and comparison groups (mean 14.17, sd 6.45). Higher GHQ score indicates poorer outcomes, so outcomes appear better for the IB group carers.<br><br>• Total sample = 58 IB, 66 C). No statistical difference between IB (mean 1.90 sd 0.65) and comparison groups (mean 1.66, sd 0.76). Higher scores indicate lower levels of need, outcomes appear better for IB group carers. When broken down by the five ASCOT domains, only one was significant. Carers in the IB group were significantly more likely to report that they were fully occupied in activities of their choice (IB 38% with "no needs", C 20% with "no needs", p<0.05).<br><br>• Total sample = 58 IB, 69 C). 59% of IB group carers and 55% of control group carers report positive outcomes (top 2 scale-points). 13% of IB group carers and 6% of control group carers report negative outcome (bottom 2 scale-points).<br><br>• Total sample = 55 IB, 62 C). The difference for each item within the three components did not reach statistical significance, but there was a trend to support the view that carers in the IB group were more likely to appraise the caregiving role positively compared with those in the comparison group. |

| | | | |
|---|---|---|---|
| | | • Satisfaction with services<br>*(7-point scale from "very dissatisfied" to "very satisfied")* | Negative impact, mean(sd): IB 21.2(4.33), C 20.26(4.78)<br>Positive impact: IB 13.38(2.52), C 12.84(2.13)<br>Quality of Service: IB 9.96(3.13), C 10.02(3.09)<br>• No statistically significant difference in satisfaction. 22% of carers in the IB group and 18% of carers in the comparison group were either extremely or very satisfied with the help that the service users received. (note people interviewed by telephone were significantly more likely to report being satisfied than those interviewed face-to-face) |
| 12. | Hagglund (2004) | • Satisfaction<br>*(measured from two instruments: the Patient Satisfaction Questionnaire PSQ-III and the Group Health Association of America GHAA, and reflects the person's opinion about the quality of services. Three satisfaction factors were derived: 1. Service Quality, 2. Daily Living Satisfaction, and 3. Community Living Satisfaction)*<br>• Unmet needs<br>*(an adapted version of the Client Questionnaire, which defines unmet service needs as the number of times in the past month when the participant was not able to do ADLs or IADLs because help was not available)*<br>• Quality of Life | • Participants in the consumer-directed programme reported greater satisfaction compared to the agency-directed group, with difference between groups compared using the Wilcoxon Rank Sum Test. Specifically, participants had significantly higher ratings of Daily Living Satisfaction (consumer = 5.49 (sd 2.22), agency = 6.41 (sd 2.43), p=0.02) and Community Living (consumer = 1.31 (sd 0.74), agency = 2.56, p<0.01). The rating for Service Quality was greater in the consumer-directed group (14.29) compared to the agency-direct group (15.90), but not significantly (p=0.18). Lower scores mean improved satisfaction.<br>• There were no significant unmet need difference between the two groups. Participants in both groups reported high levels of unmet needs, including unmet bowel and bladder needs, and being unable to eat when hungry. Unmet ADL needs (consumer = 0.55 (0.76), agency = 0.87 (1.29), p=0.51) and Unmet IADL needs (consumer = 0.85 (1.06), agency = 0.87, p=0.78), where lower score means fewer unmet needs. |

| | | | |
|---|---|---|---|
| | | *(measured using questions from the SF-36, defined as the perceptions of emotional, social and physical well-being. Two QoL factors were derived: 1. Emotional and Social Well-being, and 2. Physical well-being)* | • There were no significant difference in quality of life variables between the two groups. Consumer-directed participants reported a greater improvement in quality of life in both factors: (1) Emotional and Social Well-being: consumer = 9.42 (sd 3.87), agency = 10.33 (sd 4.22), p=0.25, and (2) Physical Well-being: consumer = 10.31 (3.44), agency = 11.0 (sd 3.44), p= 0.29; but they were not significant. Lower scores mean improved quality of life. |
| 13. | Leuci et al. (2021) | • Quality of life (measured using three scales : BPRS, GAF and HoNOS) | • FEP/PHB- subgroup T0 versus T1<br>HoNOS Behavioural Problems Mean Difference (MD) 1.63 (SE 0.36) p=0.0001<br>HoNOS Psychiatric Symptoms MD 3.08 (SE 0.52) p=0.001<br>GAF score MD -12.47 (SE 2.05) p=0.0001<br>• FEP/PHB+ subgroup T0 versus T1<br>HoNOS Behavioural Problems MD 1.72 (SE 0.27) p=0.0001)<br>HoNOS Psychiatric Symptoms MD 4.19 (SE 0.36)<br>GAF score MD -14.37 (SE2.12) p=0.0001<br><br>• FEP/PHB- subgroup T0 versus T2<br>HoNOS Behavioural Problems Mean Difference (MD) 1.92 (SE 0.45) p=0.0001<br>HoNOS Psychiatric Symptoms MD 3.50 (SE 0.70) p=0.001<br>GAF score MD -12.39 (SE 2.65) p=0.0001<br>• FEP/PHB+ subgroup T0 versus T2<br>HoNOS Behavioural Problems MD 2.66 (SE 0.36) p=0.0001)<br>HoNOS Psychiatric Symptoms MD 5.08 (SE 5.98)<br>GAF score MD -20.46 (SE 2.06) p=0.0001 |
| 14. | Pelizza et al. (2022) | • Quality of life (measured using three scales : BPRS, GAF and HoNOS) | • Linear regression results on functioning and psychopathological characteristics (2 year follow up) on the total group (n=127)<br>• T2−T0 |

| | | | |
|---|---|---|---|
| | | | Delta BPRS 'Affective' factor subscore<br>Multiaxis PHB intervention −1.967 (SE 1.902) p=0.305<br>Delta BPRS 'Positive' factor subscore<br>Multiaxis PHB intervention −1.124 (SE 1.719) p=0.516<br>Delta BPRS 'Activation' factor subscore<br>Multiaxis PHB intervention −1.022 (SE 1.697) p=0.549<br>Delta BPRS 'Negative' factor subscore<br>Multiaxis PHB intervention −2.924 (SE 1.103) p=0.010<br>Delta BPRS 'Disorganization' factor subscore<br>Multiaxis PHB intervention −1.284 (SE 1.216) p=0.295<br>Delta HoNOS 'Behavioral Problems' subscale subscore<br>Multiaxis PHB intervention −1.807 (SE 0.928) p=0.056<br>HoNOS 'Impairment' subscale subscore<br>Multiaxis PHB intervention 0.259 (SE 0.714) p=0.718<br>Delta HoNOS 'Psychiatric Symptoms' subscale subscore<br>Multiaxis PHB intervention 0.293 (SE 1.190) p= 0.806<br>Delta HoNOS 'Social Problems' subscale subscore<br>Multiaxis PHB intervention −2.838 (SE 1.306) p=0.034<br>Delta GAF score<br>Multiaxis PHB intervention 7.521 (SE 4.265) p=0.083 |
| 15. | Wiener (2007) | • Satisfaction with services<br>*(Constructed an 8-item Satisfaction with PAID Personal Assistance Scale (SPPAS) ranging from 0-100. Cronbach's alpha 0.7.)* | • Average SPPAS ratings were higher for consumer-directed participants (91.65) than for those with agency-directed care (88.68) but the difference was not significant for adults younger than 65, p>0.05. |
| 16. | Woolham (2013) | • General Health Questionnaire (GHQ)<br>*(measures both psychological well-being and mental distress)* | • For the younger group (<65), the budget holder group had significantly better scores (mean 10.12, SD=6.93) compared with traditional users (13.28, SD=7.37), p<0.001. Note Woolham only reports differences in scores between older and younger people, Fleming compared scores between intervention and control. |

| | | | |
|---|---|---|---|
| | | • Activities of Daily Living Scale (ADL) (assesses the ability to carry out everyday activities of daily living) | • For the younger group (<65), there was no significant difference between the budget holder group (11.77, SD=3.59) compared with traditional users (11.93, SD=3.72), p=0.69. Note Woolham only reports differences in scores between older and younger people, Fleming compared scores between intervention and control. |
| | | • Mean package costs | • Costs per week<br>Learning disabilities: PB £412.06 (n=53), Trad: £337.30 (n=96)<br>Mental health: PB £383.51 (n=4), Trad: £116.57 (n=4)<br>Physical disabilities: PB £298.84 (n=61), Trad: £202.59 (n=91) |
| | | • Cost effectiveness (scatterplots only) | • Only presented for the entire group including older people.<br>Using ADL scores, scatterplots show little difference in respect of benefit between the two groups, and a significant increase in the distribution of costs for the PB group.<br>Using GHQ scores, the traditional service group were more likely than average to be experiencing some degree of ill-being. However, costs were lower than average. Amongst budget holders, GHQ scores suggested higher well-being but costs were also great than average.<br>There was evidence of benefit to PB users on the GHQ scale, but for both outcome measures, these benefits came at a greater financial cost. |

Table S13. Annual expenditures for children with intellectual and developmental disabilities in the Cash and Counseling Demonstration (Dale et al., 2004)

| Enrolment year | Expenditure | Treatment group | Control group | Difference |
|---|---|---|---|---|
| First | Medicaid waiver | $15,966 | $12,647 | $3319* |
| | Total Medicaid | $29,974 | $29,095 | $880 |
| Second | Medicaid waiver | $18,859 | $14,046 | $4812* |
| | Total Medicaid | $33,458 | $30,877 | $2581* |

*indicates statistical significance

Table S14. Annual expenditures in the Cash and Counseling Demonstration (Dale & Brown, 2005)

| Demonstration site | Expenditure | Treatment group | Control group | Difference |
|---|---|---|---|---|
| Arkansas | Medicaid personal care | $5435 | $2430 | $3005* |
| | Medicaid non-personal care | $8689 | $10,432 | -$1743* |
| | Combined Medicare and Medicaid | $20,111 | $18,747 | $1365 |
| New Jersey | Medicaid personal care | $11,166 | $9220 | $1946* |
| | Medicaid non-personal care | $15,697 | $16,829 | -$1132 |
| | Combined Medicare and Medicaid | $37,749 | $36,394 | $1355 |
| Florida | Medicaid waiver | $22,017 | $18,321 | $3696* |
| | Medicaid non-waiver | $5416 | $5785 | -$369 |
| | Combined Medicare and Medicaid | $29,867 | $26,849 | $3018* |

*indicates statistical significance

Table S14: in Arkansas, Medicaid personal care expenditures were significantly higher for the treatment group, but this was offset by significantly lower non-personal care expenditures, primarily due to lower expenditures for Medicaid nursing facilities and inpatient care. In New Jersey, Medicaid personal care expenditures were significantly higher for the treatment group. Non-personal care expenditures were lower for the treatment group but not significantly so, with Medicaid home health care being the only significantly lower expenditure. For adults in Florida, Medicaid waiver expenditures were significantly higher for the

treatment group. Non-waiver expenditures were lower, but not significantly. The only service expenditure that was significantly lower for the treatment group was Medicare nursing facilities.

Table S15. Cost per recipient per month in the Cash and Counseling Demonstration (Brown et al., 2007)

| Demonstration site | Treatment group | Control group | Difference |
|---|---|---|---|
| Arkansas | $513 | $422 | $91* |
| New Jersey | $1,153 | $1,106 | $47* |
| Florida – adults | $1,884 | $1,593 | $291* |

*indicates statistical significance

Table S16. Mean weekly package costs (Glendinning et al., 2008)

| Disability (n) | Individual budget holders | Traditional service users |
|---|---|---|
| Learning disabilities (133) | £359 | £390 |
| Mental health conditions (68) | £149 | £152 |
| Physical disabilities (178) | £310 | £334 |
| All – including older people (518) | £279 | £296 |

Note: no statistically significant difference was found between budget and control groups

Table S17. Mean weekly package costs (Glendinning et al., 2008)

| Disability (n) | Service | Individual budget holders | Traditional service users |
|---|---|---|---|
| Learning disability (133) | Home care | £48 | £65 |
| | Personal assistant | £112 | £58* |
| | Independent living fund | £11 | £66* |
| | Social worker/care manager | £20 | £10 |
| Mental health conditions (68) | Home care | £5 | £7 |
| | Personal assistant | £39 | £24 |
| | Independent living fund | £0 | £7 |
| | Social worker/care manager | £24 | £25 |
| Physical disability (178) | Home care | £24 | £82* |
| | Personal assistant | £144 | £73* |
| | Independent living fund | £14 | £39* |

| | | | |
|---|---|---|---|
| | Social worker/care manager | £16 | £8* |
| All – including older people (518) | Home care | £37 | £70* |
| | Personal assistant | £100 | £52* |
| | Independent living fund | £8 | £30* |
| | Social worker/care manager | £18 | £11 |

*indicates statistical significance

Table S17: both the physical disability and learning disability treatment groups recorded significantly higher mean weekly costs for personal assistants and the Independent Living Fund compared with the control group. In addition, the physical disability treatment group recorded significantly higher costs compared with the control group for home care and care manager.

Table S18**.** Mean cost per week by health service

| Health resource | Individual budget holders | Traditional services users |
|---|---|---|
| Day hospital in last month | £10 | £9 |
| Nurse in last month | £32 | £24 |
| Therapist in last 3 months | £3 | £3 |
| GP in last 3 months | £5 | £4 |
| A&E in last 3 months | <£1 | <£1 |
| Chiropodist in last 3 months | £1 | £1 |
| In-patient service in last 6 months | £33 | £19* |

*indicates statistical significance

Table S18: No breakdown of cost difference by disability subgroup was reported, however, the extent to which health service use and cost differed between the disability groups was stated and it was found that there was a significantly higher health cost per week amongst older people (£107) compared with people with a physical disability (£76), learning disability (£23) and mental health condition (£76).

Table S19**.** Mean weekly package costs (Woolham & Benton, 2013)

| Disability (n) | Personal budget holders | Traditional service users |
|---|---|---|
| Learning disabilities (149) | £412.06 | £337.30 |
| Mental health conditions (8) | £383.51 | £116.57 |
| Physical disabilities (152) | £298.84 | £202.59 |
